# Cardiomyopathy: pathogenesis and therapeutic interventions

**DOI:** 10.1002/mco2.772

**Published:** 2024-10-25

**Authors:** Shitong Huang, Jiaxin Li, Qiuying Li, Qiuyu Wang, Xianwu Zhou, Jimei Chen, Xuanhui Chen, Abdelouahab Bellou, Jian Zhuang, Liming Lei

**Affiliations:** ^1^ Department of Cardiac Surgical Intensive Care Unit Guangdong Cardiovascular Institute Guangdong Provincial People's Hospital (Guangdong Academy of Medical Sciences) Southern Medical University Guangzhou China; ^2^ Department of Cardiovascular Surgery Zhongnan Hospital of Wuhan University Wuhan China; ^3^ Department of Cardiovascular Surgery Guangdong Cardiovascular Institute Guangdong Provincial People's Hospital (Guangdong Academy of Medical Sciences) Southern Medical University Guangzhou China; ^4^ Department of Cardiovascular Surgery Guangdong Provincial Key Laboratory of South China Structural Heart Disease Guangzhou China; ^5^ Department of Medical Big Data Center Guangdong Provincial People's Hospital (Guangdong Academy of Medical Sciences) Southern Medical University Guangzhou China; ^6^ Department of Emergency Medicine, Institute of Sciences in Emergency Medicine Guangdong Provincial People's Hospital (Guangdong Academy of Medical Sciences) Southern Medical University Guangzhou China; ^7^ Department of Emergency Medicine Wayne State University School of Medicine Detroit Michigan USA

**Keywords:** cardiomyopathy, disease‐causing gene, gene therapy, pathogenesis, personalized medicine, therapeutic interventions

## Abstract

Cardiomyopathy is a group of disease characterized by structural and functional damage to the myocardium. The etiologies of cardiomyopathies are diverse, spanning from genetic mutations impacting fundamental myocardial functions to systemic disorders that result in widespread cardiac damage. Many specific gene mutations cause primary cardiomyopathy. Environmental factors and metabolic disorders may also lead to the occurrence of cardiomyopathy. This review provides an in‐depth analysis of the current understanding of the pathogenesis of various cardiomyopathies, highlighting the molecular and cellular mechanisms that contribute to their development and progression. The current therapeutic interventions for cardiomyopathies range from pharmacological interventions to mechanical support and heart transplantation. Gene therapy and cell therapy, propelled by ongoing advancements in overarching strategies and methodologies, has also emerged as a pivotal clinical intervention for a variety of diseases. The increasing number of causal gene of cardiomyopathies have been identified in recent studies. Therefore, gene therapy targeting causal genes holds promise in offering therapeutic advantages to individuals diagnosed with cardiomyopathies. Acting as a more precise approach to gene therapy, they are gradually emerging as a substitute for traditional gene therapy. This article reviews pathogenesis and therapeutic interventions for different cardiomyopathies.

## Graphical abstract

Cardiomyopathy is a group of diseases characterized by structural and functional damage to the myocardium. Many specific gene mutations, environmental factors, and metabolic disorders may cause cardiomyopathy. Traditional therapeutic includes drug and surgery. With the growing comprehension of the molecular mechanisms underlying cardiomyopathy. Gene therapy and cell therapy has become a tool for the clinical treatment of disease.

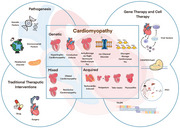

## 1 INTRODUCTION

Cardiomyopathies are a group of heterogeneous diseases characterized by morphological and functional abnormalities of the heart, leading to a wide spectrum of clinical manifestations, from asymptomatic left ventricular (LV) dysfunction to severe heart failure and sudden cardiac death (SCD). Cardiomyopathy is the predominant indication for pediatric heart transplantation, especially in children over the age of one.^1^ With increasing recognition of their impact on public health, cardiomyopathies have become a major focus in the field of cardiology.

**FIGURE 1:**
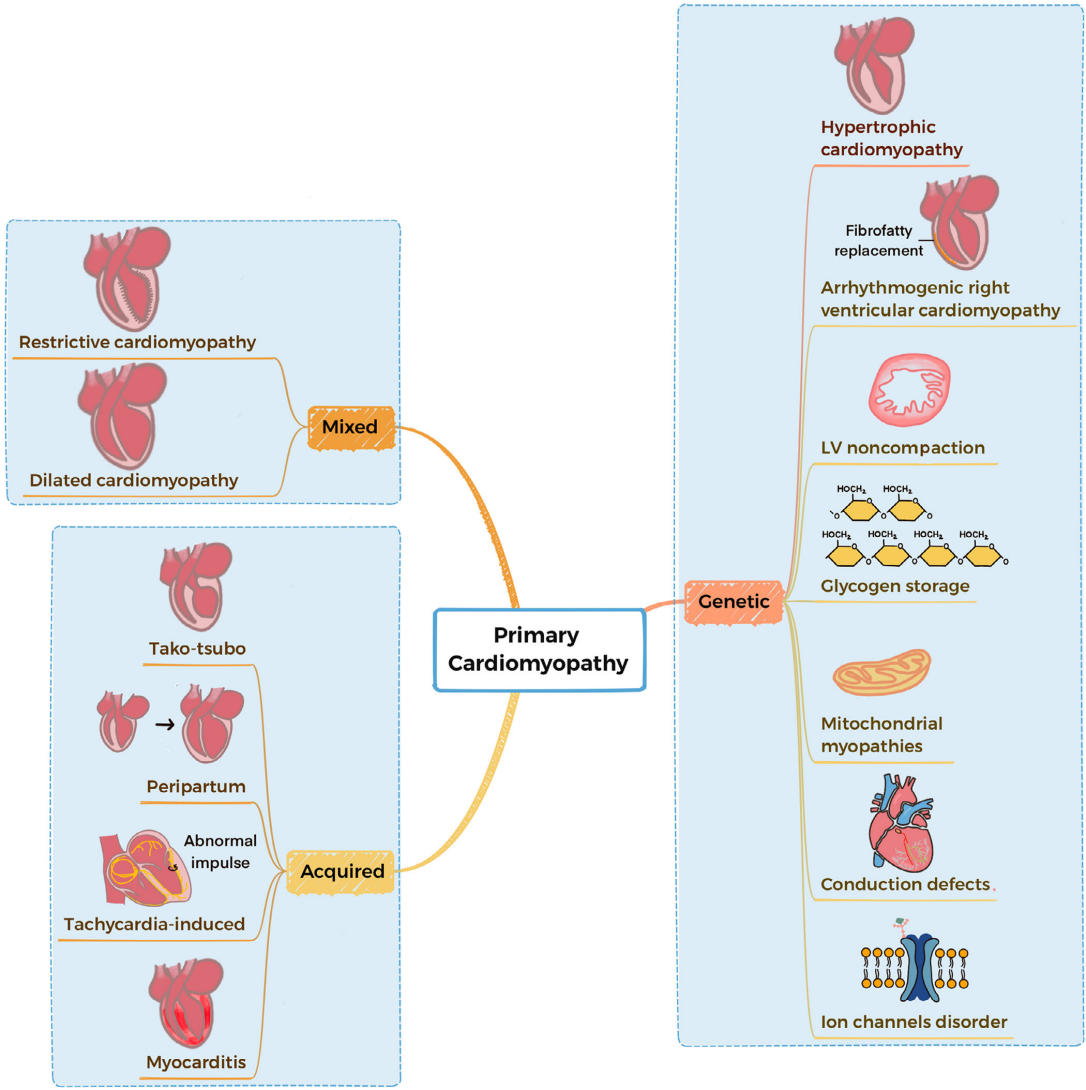
Classification of primary cardiomyopathies according to the AHA 2006 classification system. Primary cardiomyopathies can be divided into three categories: genetic, mixed, and acquired. Mixed cardiomyopathies include DCM and RCM. Acquired cardiomyopathy is the disease due to inflammatory stimulation and other factors, which mainly include four cardiomyopathies. Notably, recent evidence has emerged suggesting that a significant number of peripartum cardiomyopathy cases have genetic foundations. Genetic cardiomyopathy encompasses a collection of diseases resulting from gene mutations that induce abnormalities in both the structure and function of the cardiac muscles. There are many different types of genetic cardiomyopathies.

According to the American Heart Association (AHA) 2006 classification system, primary cardiomyopathies are diseases characterized by having direct, targeted impacts on the heart muscles.^2^, ^3^ The most effective categorization of cardiomyopathies is through their classification as primary or secondary. Primary cardiomyopathies can be classified as genetic, mixed (genetic and nongenetic), or acquired (Figure 1).^3^ Breakthroughs in the fields of genomics and proteomics have illuminated the underlying molecular mechanisms behind hereditary, mixed, and acquired cardiomyopathies.

In recent years, with the growing comprehension of the molecular and cellular mechanisms underlying cardiomyopathy, the molecular mechanism of disease‐causing mutations is increasingly being discovered. Genetic factors have been identified as a significant component in the etiology of many cardiomyopathies, with hundreds of genes linked to disease development, we can facilitate early diagnosis and therapy by periodic screening. The advent of gene therapy, cell therapy, and precision medicine offers promising avenues for disease modification and personalized care. Ongoing investigations involve the exploration of genetic manipulation using specific viral vectors and genome editing strategies including antisense oligonucleotides (AONs), transcription activator‐like effector nucleases (TALENs), AAV, iPSCs, and CRISPR systems. In the future, they have the potential to emerge as a promising therapeutic approach for primary cardiomyopathies, offering in vivo genome editing capabilities.^4^

Despite these advances, challenges remain in the early diagnosis, risk stratification, and management of cardiomyopathies. There is a pressing need for continued research to elucidate the complex interplay between genetic, environmental, and lifestyle factors that contribute to the development and progression of these diseases. This review will delve into the intricate details of the pathogenesis of different types of cardiomyopathies, explore the current landscape of therapeutic interventions, and highlight the emerging strategies that hold the potential to transform the future of cardiomyopathy.

## 2 GENETICS AND CARDIOMYOPATHY

### 2.1 Genetics in the pathogenesis of cardiomyopathy

Cardiomyopathies are a diverse array of conditions, each with a unique set of genetic underpinnings, that manifest as structural and functional anomalies within the myocardium.^5^ In recent decades, the advent of next‐generation sequencing (NGS) has revolutionized genetic analysis, rendering it more accessible than ever before. Utilizing NGS, high‐throughput genetic research has yielded significant insights into the genetic architecture of cardiomyopathies, leading to the identification of numerous associated gene mutations.^6^ These genetic variations can impair the heart's ability to systole and dilate, thereby influencing the initiation and progression of the disease. Furthermore, as the correlation between clinical phenotypes and their genetic determinants becomes more defined, there is a growing understanding of the underlying mechanisms and the potential to develop innovative therapeutic strategies. Recent findings suggest that the development of cardiomyopathy is influenced not only by rare genetic variants but also by common genetic variations.

Genes are pivotal in the pathogenesis of cardiomyopathies, with myocardial structural and functional abnormalities often stemming from genetic influences or environmental triggers. The goal of gene therapy is to ameliorate disease symptoms by introducing new genetic material or by genetically modifying existing genes and their regulatory sequences. This is achieved through strategies such as gene replacement and gene editing. Consequently, uncovering the genetic mechanisms underlying cardiomyopathies and their progression is essential for establishing a foundation for effective gene therapy.^7^

The molecular mechanisms through which specific mutations precipitate cardiomyopathy can be categorized into three types: (1) loss of function, typically arising from nonsense or frameshift mutations that result in the production of partially or entirely nonfunctional proteins, or even in the complete absence of the protein due to nonsense‐mediated decay; (2) gain of function, often due to missense mutations that lead to the creation of a protein with enhanced or altered activity compared with its wild‐type counterpart; and (3) dominant negative effects, usually caused by missense mutations that impede the normal biological function of the wild‐type protein, a phenomenon most frequently observed in proteins that form homomeric complexes, where the mutant subunits can disrupt the complex assembly.^8^

Hypertrophic cardiomyopathy (HCM) is one of the most common hereditary cardiomyopathies, usually caused by mutations in genes encoding sarcomere proteins. These genes include *beta‐myosin heavy chain* (*MYH7*), *myosin‐binding protein C* (*MYBPC3*), and *troponin T2* (*TNNT2*). These mutations can lead to abnormal contraction of cardiac muscle cells, causing myocardial hypertrophy and heart dysfunction.^9^, ^10^, ^11^, ^12^

The hereditary form of dilated cardiomyopathy (DCM) is usually associated with mutations in various genes encoding structural proteins of cardiac muscle cells, such as *titin (TTN), actin (ACTC1)*, and *MYBPC3*.^13^ Mutations in these genes can lead to structural and functional abnormalities of cardiac muscle cells, ultimately resulting in ventricular dilation and reduced heart pumping function.

Arrhythmogenic right ventricular cardiomyopathy (ARVC) is a cardiomyopathy characterized by the gradual replacement of myocardium with fat and fibrous tissue. Its hereditary form is associated with mutations in genes encoding cardiac cell junction proteins, such as *desmoplakin (DSP), plakophilin‐2 (PKP2)*, and *desmoglein‐2 (DSG2)*.^14^, ^15^, ^16^ These mutations affect the intercellular connections of cardiac muscle cells, leading to ventricular dysfunction and arrhythmias.

The hereditary form of restrictive cardiomyopathy (RCM) is rare, but mutations in certain genes, such as the gene for *TTN*, are known to be associated with it.^17^ Left ventricular noncompaction (LVNC) is a cardiomyopathy characterized by an excessive and abnormal trabeculation in the LV cavity. This condition may be associated with mutations in various genes, including those encoding sarcomere proteins.^18^ These cardiomyopathies involve mutations in genes of metabolic pathways, such as glycogen metabolism disorders, fatty acid oxidation disorders, lysosomal storage diseases, and mitochondrial diseases. These diseases affect the energy metabolism of cardiac muscle cells, leading to heart dysfunction.

### 2.2 Genetics in the diagnosis of cardiomyopathy

Genetic testing is crucial for the early diagnosis and differential diagnosis of patients with cardiomyopathies and their family members. For example, for patients with DCM, LMNA, and sodium voltage‐gated channel alpha subunit 5 (SCN5A) genetic testing is recommended if they have associated cardiac conduction diseases or a family history of sudden death at an early age.^19^ In patients with HCM, if a pathogenic gene mutation has been identified in the family, family members should undergo genetic testing to help reclassify the mutation level and intervene early in high‐risk members.^20^ In addition, genetic counseling is an essential component of the genetic testing and family screening process, helping patients and family members understand genetic risks and providing advice on prevention, management, and family planning.

It is important to note that genetic test results are usually probabilistic rather than deterministic, so they need to be interpreted in conjunction with the patient's medical and family history. For example, family history information and the distribution of presumed disease‐related mutations in the family may be important for guiding clinical interpretation, especially when identifying new genetic variants. In addition, family studies have noted that up to 10% of ARVC families have multiple pathogenic variants.^21^

When conducting genetic testing, it is important to consider using large gene panels, as these panels can increase the likelihood of identifying the molecular cause, especially when patients exhibit mixed phenotypes or lack typical features. However, as the number of genes tested increases, the possibility of identifying variants of uncertain significance (VUS) also increases, adding complexity to interpretation and genetic counseling.^22^

In summary, genetic testing and family screening for cardiomyopathies are key components in managing these diseases, helping with early diagnosis, risk assessment, and the formulation of personalized treatment strategies.^23^ As our understanding of the genetic basis of cardiomyopathies deepens, our understanding and treatment of cardiomyopathies will become more precise and effective.

## 3 GENERAL THERAPEUTIC INTERVENTIONS OF CARDIOMYOPATHIES

The management of cardiomyopathies is multifaceted, requiring a tailored approach that considers the specific type of cardiomyopathy, the severity of symptoms, and the presence of complications. This section outlines the general therapeutic interventions applicable to various forms of cardiomyopathies.

### 3.1 Pharmacological therapy

Pharmacological interventions are the cornerstone of cardiomyopathy treatment, aimed at alleviating symptoms, improving cardiac function, and reducing the risk of complications. Common pharmacological agents include beta‐blockers, calcium channel blockers, diuretics, inotropic agents, and antiarrhythmic medications.^24^

### 3.2 Surgical interventions

The surgical treatment of cardiomyopathy includes a variety of different surgical procedures, which specifically depend on the type of cardiomyopathy and the patient's condition.

Cardiac resynchronization therapy (CRT) is a therapeutic option for patients with DCM who have intraventricular conduction delays and for patients with HCM who have reduced left ventricular ejection fraction (LVEF), improving cardiac synchronization and function.^25^ Sidhu et al.^26^ observed that systolic function is enhanced in patients with LMNA‐related cardiomyopathy following CRT. It involves the use of a specialized pacemaker that stimulates both ventricles of the heart to contract in a coordinated manner.

Current guidelines advocate the use of implantable cardioverter‐defibrillators (ICDs) for primary prevention of SCD in patients with symptomatic nonischemic cardiomyopathy who have LVEFs below 35%.^27^ These devices monitor heart rhythm and deliver a shock to restore normal rhythm if a life‐threatening arrhythmia is detected.

Surgical treatment for cardiomyopathy encompasses a variety of procedures, contingent upon the type of cardiomyopathy and the specifics of the patient's condition. For instance, in the case of HCM, particularly for patients with left ventricular outflow tract obstruction (LVOTO), septal myectomy (Morrow surgery) is a commonly employed surgical approach.^28^

For end‐stage cardiomyopathy, heart transplantation remains the ultimate therapeutic option, providing a potential cure and significant improvement in quality of life. Candidates for transplantation are carefully selected based on medical criteria and the severity of their condition.^29^

### 3.3 Gene therapy

The concept of gene therapy emerged during the 1970s. Despite the initially high expectations, early development in this field encountered setbacks. Genetic analysis has advanced significantly with the development of NGS. Recent studies showed that the variation of the disease‐causing genes plays a critical role in the morbidity and development of cardiomyopathy. An increasing number of cardiomyopathies have been recognized as monogenic diseases with a genetic component.^7^, ^30^, ^31^ Gacita et al.^32^ proved that promoters and enhancers modify the expression of cardiomyopathy genes, so genetic variations in promoters and enhancers within the human genome could potentially contribute to the development of cardiomyopathy. The mutations in the noncoding parts of the genome are also involved in the pathogenesis of cardiomyopathy, namely, microRNA, promoter elements, enhancer/silencer elements, and long noncoding RNAs (lncRNAs), so that therapeutic genes are not limited only to protein‐coding complementary DNAs, thus broadening the spectrum of possible applications.^33^, ^34^

**FIGURE 2:**
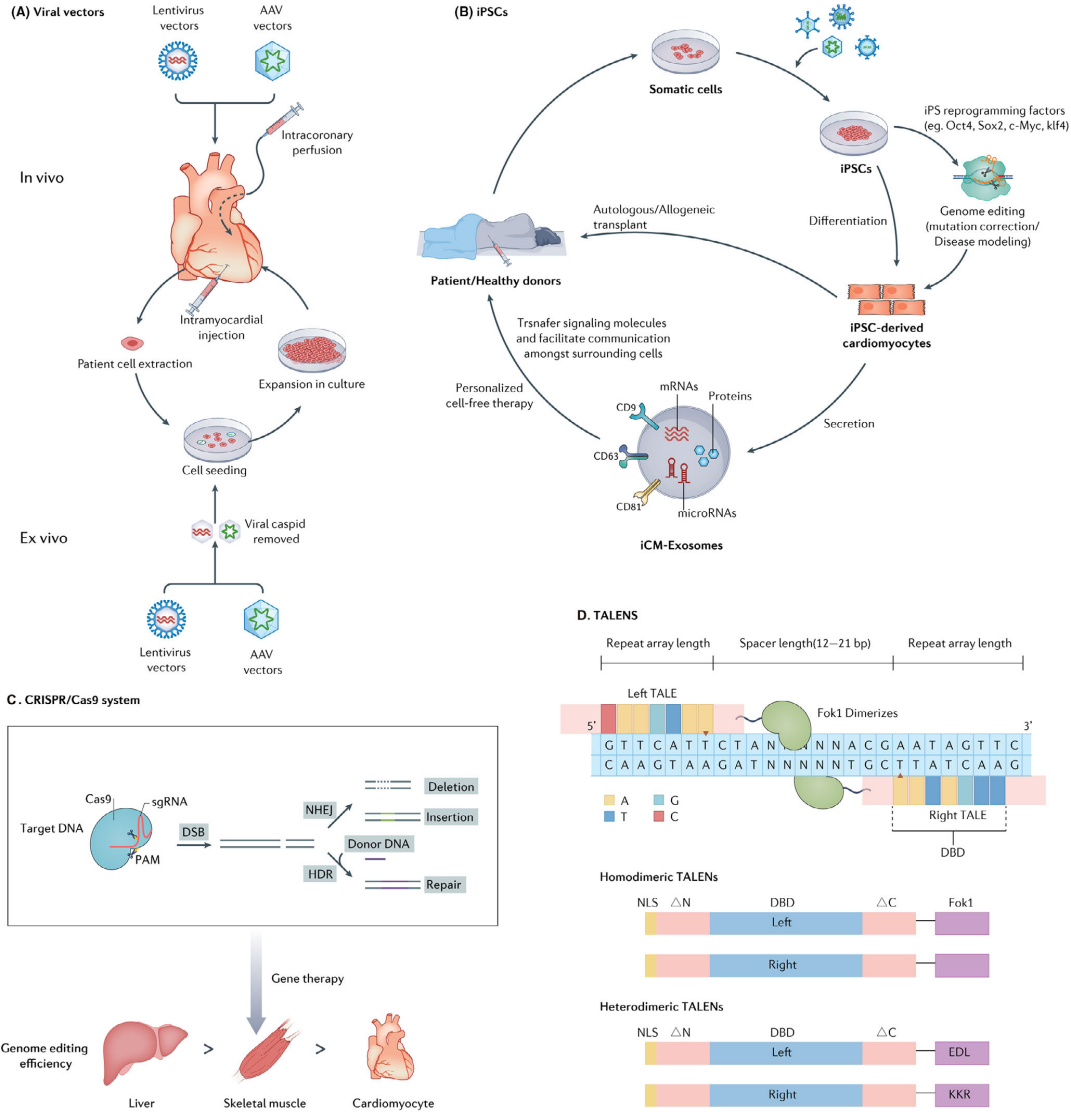
Mechanisms of general strategies and techniques in therapy of cardiomyopathy. (A) Viral vectors. In vivo, the viral vectors are delivered into the cardiac via IM injection and IC perfusion. In ex vivo, cells are extracted from the blood vessels of patients and seeded in a culture dish; after the viral capsid is removed, viruses are transduced in the culture containing patient cells, and they expand in the culture dish together. (B) iPSCs. iPSC cells can be modified with iPSC reprogramming factors or genome editing and differentiated into cardiomyocytes to be transplanted back into the patients. The iPSC‐derived cells can secrete exosomes, which mediate intercellular communication in heart exosomes. (C) CRISPR/Cas9 system. The Cas9 enzyme is used to cut two strands of DNA on a specific area as a pair of molecular scissors to remove or add DNA. gRNA can specifically identify the piece of DNA that needs to be cut. After the double‐strand DNA is broken, the broken DNA can be joined through NHEJ or HDR. (D) TALENs. The TALEN is composed of TALE protein, which contains customizable DNA‐binding domains (DBD) and nuclease domains of FokI dimerizes. Residues 12 and 13 (repeat variable diresidues (RVDs)) in TALE are responsible for the recognition of a specific base. The FokI nuclease bonds together to the protein through a wild‐type TALE sequence. TALEN‐ELD and TALEN‐KKR for heterodimeric TALENs include mutated Fok I dimerizes named ELD and KKR. ΔN and ΔC represent truncated N‐terminal and C‐terminal domains of TALEN. Left DBD and right DBD were customized to bind closely. All of the TALENs contain an SV40 nuclear localization signal (NLS).

Therefore, defining the associations between gene variation, gene expression, and disease as well as novel potential therapeutic targets will accelerate progress in the study of cardiomyopathies.^7^, ^35^ Cardiac gene‐targeted therapies include identifying pathogenic genes, selecting appropriate vectors, constructing the gene of interest, and transporting organ‐targeted vectors. There are some standard techniques that introduce exogenous genes in cardiac gene therapies (Figure 2).

#### 3.3.1 Viral vectors

Lentivirus and adeno‐associated viruses (AAVs) are the most commonly used viral vectors. With strong cardiomyocyte transduction ability and low immunogenic response, the improved lentivirus has been tested to treat various human diseases.^36^, ^37^, ^38^, ^39^

Lentiviral vectors are frequently employed for gene transfer within the nervous system. They have a large capacity of 10 kb. Thus, adequate genes could be transferred in vivo via lentiviral vectors, and they could stabilize long‐term transgene expression by integrating into the chromosomes of transduced cells.^40^ Although lentiviral vector gene therapy has many advantages, it also has some disadvantages: lentiviral vectors may cause immune responses in the host, which could not only reduce the therapeutic effect but also cause adverse immune‐related side effects. In addition, there are still potential biosafety issues with the viruses. Future research needs to further improve the lentiviral vector system to enhance its safety and application efficiency.^41^

AAV, a nonenveloped virus, can be employed as a vehicle to transport DNA into specific target cells.^42^ The AAVs vector is genetically modified to avoid integration into the host cell genome, thereby ensuring its stable expression within the host cells over an extended period, lasting long time.^43^, ^44^ Even though AAVs, with their single administration, can provide stable long‐term expression and high efficiency in cardiomyocytes, and despite their lower inflammatory profile compared with other viral vectors, the host's immune responses to the vector components and the products of the transgene still pose challenges to the efficacy and safety of gene therapy.^45^ AAVs vector has already been widely used for phenotypic assessment and heart failure prevention in animal models of cardiomyopathy.^46^, ^47^ AAV vector technology is mature and rapidly progressing in various practical and therapeutic applications. In mice models, specific targeting of the heart can be achieved after systemic administration by utilizing a combination of cardiac AAV serotype and a promoter that is specific to cardiomyocytes, as demonstrated by various studies.^33^, ^48^ Moreover, isolating novel AAV variants with increased tropism for cardiomyocytes can refine AAV delivery methods, thereby increasing cardiomyocyte transduction.^49^ The integration of AAV vector‐derived DNA into the host genome may have potential carcinogenicity and may also interfere with the function of normal genes, causing genomic instability.^50^ Therefore, it is necessary to conduct long‐term monitoring for patients who have received AAV gene therapy.^51^

The most widely used gene delivery techniques via viral vectors are intramyocardial (IM) injection and intracoronary (IC) perfusion. IM injection is a catheter‐based minimally invasive procedure that directly injects a virus carrying a gene or a cytokine into the tissue to increase the local concentration of the vectors. It has the highest local retention.^52^ However, uniform diffusion of the carrier tissue is difficult to achieve with direct IM injection, and the proportion of cells transfected is insufficient for ideal therapeutic effects. Furthermore, the injection efficiency of IC injection may be reduced due to the rapid coronary circulation.^53^, ^54^, ^55^, ^56^ Based on these limitations, coronary venous retro‐infusion has garnered significant attention for therapeutic strategies aimed at precisely delivering drugs, genes, or cells to the ischemic myocardium. It can not only integrate homogenous intravascular delivery, but also increase the retention of angiogenic substrates.^57^, ^58^

In addition to IM injection and IC perfusion, intravenous (IV) administration and local delivery techniques are also pivotal in the arsenal of gene delivery methods utilizing viral vectors. Salami et al.^59^ discovered that a single IV injection of 10^11 genome copies of AAVrh.10hFXN, an AAV serotype rh10 vector engineered to deliver the human *FXN* gene, effectively corrected the stress‐induced ejection fraction and fractional shortening phenotypes. Kevany et al.^60^ identified a novel AAV capsid that enhances tissue specificity and expression within the target tissues, namely, the heart, muscle, and central nervous system, following IV administration. Vassalli et al.^61^ successfully instilled AAV vectors into the pericardial space, achieving transduction of epicardial myocytes in mice, achieving transduction of epicardial myocytes in mice.

#### 3.3.2 RNA editing

Recent studies have suggested that the mutant pre‐mRNA could be repaired by trans‐splicing to treat autosomal‐dominant diseases. Mearini et al.^62^ demonstrated that Mybpc3 mRNA could be repaired by 5′‐trans‐splicing in cardiac myocytes of homozygous Mybpc3‐KI mice.

Another prevalent RNA editing technique is spliceosome‐mediated RNA trans‐splicing, which entails the fusion of two distinct RNA molecules to generate a complete, repaired mRNA through splicing.^63^

The third strategy of RNA editing is mRNA silencing. Jiang et al.^64^ demonstrated that AAV‐mediated RNAi delivery allele‐specific silenced the mutant Myh6 mRNA and the manifestation of the disease phenotype was postponed in heterozygous Myh6‐KI mice. In another study, Bongianino et al.^65^ demonstrated the efficacy of allele‐specific silencing by RNA interference (RNAi) to prevent catecholaminergic polymorphic ventricular tachycardia phenotypic manifestations in a mouse model.

#### 3.3.3 Antisense oligonucleotides

Antisense oligonucleotides (AONs/TASOs) is an approach that internally deletes a small part of the targeted protein but maintains its function through in‐frame skipping of mutated exons. Thus, AONs are expected for the therapeutic correction of many genetic diseases. Gedicke‐Hornung et al.^66^ successfully transduced AONs into cardiomyocytes of neonatal mice to prevent the MYBPC3 gene mutation. Hahn et al.^67^ offered the pioneering program represents the initial systematic effort to design and evaluate AONs specifically targeting mutated TTN target exons, improving the future therapeutic potential in titin‐based DCM. Gramlich et al.^68^ demonstrated that AONs could restore disruption of the titin reading through exon skipping, as observed in both patient cardiomyocytes and mouse hearts.

#### 3.3.4 CRISPR/Cas9 systems

The CRISPR/Cas9 system used an approximately 100‐nucleotide RNA molecule derived from Streptococcus pyogenes and other bacterial species to facilitate the precise targeting of the Cas9 protein and a specific genomic site for cleavage. Consequently, the host cell activates endogenous DNA repair pathways to repair the damage by either nonhomologous end‐joining (NHEJ) or homology‐directed repair (HDR).^69^ Therefore, gene editing therapy often favors the use of HDR. However, the efficiency of HDR compared with NHEJ is typically low, primarily due to the relative rarity of HDR events in cardiomyocytes. Furthermore, even in mitotic cells, HDR is constrained to the S and G2 periods in the cell cycle.^70^ CRISPR HDR genome editing corrected MYBPC3 mutations and restored MYBPC3 protein expression, offering a promising therapeutic approach for treating HCM associated with these mutations. This underscores the immense potential of CRISPR technology in addressing hereditary heart diseases.^71^

In addition, CRISPR/Cas9 system can not only correct disease‐related mutations but also introduce protective mutations and targeted viral genome.^72^, ^73^ Thus, CRISPR/Cas9 gene editing has the potential hope for correct genetic diseases in the future of personalized medicine because of its ability to make precise gene‐specific corrections.

Despite the remarkable advancements in genome editing technology brought about by the CRISPR/Cas9 system, challenges persist. The full therapeutic potential of genome editing for cardiovascular diseases is still hindered by a range of biological and technical obstacles. It is crucial to acknowledge the variations in the efficiency of CRISPR–Cas9 genome editing in different organs within an organism. Specific attention should be given to the notably low efficiency observed in skeletal muscle and, particularly, in cardiomyocytes when compared with the liver. This distinction could be attributed to the postmitotic nature of cardiomyocytes, resulting in differences in CRISPR–Cas9 accessibility for delivery, or it may be due to inherent variations in the activity of CRISPR–Cas9 among assorted cell types.^74^

One other significant concern regarding the application of the CRISPR/Cas9 system to cardiomyocyte mutagenesis is the high frequency of off‐target effects. Although off‐target events may be infrequent, their potential impact should not be downplayed, as mutations in other genes could lead to detrimental effects. This is particularly crucial for clinical applications of genome‐edited cells or tissues, where it is imperative to entirely prevent the occurrence of off‐target effects.^75^ Another issue is immunogenicity of Cas9 protein. CRISPR–Cas9 evokes a host cellular and humoral immune response with distinct cellular and molecular signatures, indicating that Cas9 can serve as an antigen in mammals.^76^ The recent finding that routine prior exposure to Staphylococcus aureus and Streptococcus pyogenes can lead to preexisting immunity against Cas9 presents a significant challenge for its clinical advancement. Nonetheless, pioneering studies employing immune‐privileged sites, immunosuppressive strategies, or novel Cas9 sources not previously encountered offer promising avenues to navigate around these initial hurdles in gene‐editing‐based therapeutics.

#### 3.3.5 Transcription activator‐like effector nucleases

Transcription activator‐like effector nucleases (TALENs) consist of a specific DNA‐binding domain comprising tandem repeats from transcription activator‐like effectors (TALEs) found in Xanthomonas bacteria, combined with a nonspecific DNA‐cleaving nuclease domain.^77^, ^78^ TALENs, similar to zinc finger nucleases (ZFNs), offer a versatile tool for precise genome editing purposes.^79^ The utilization of TALENs enables precise and targeted genetic modifications within the human genome, offering the capability for specific site‐specific alterations of the desired gene.^80^ Recent studies have started to apply TALENs to clinical trials, which made DNA editing possible for the first time.^81^, ^82^ Furthermore, TALENs have the advantages of low cytotoxicity and stability compared with other gene‐editing technologies.

Although TALENs are a powerful genome‐editing tool, they also have some limitations and disadvantages. First, the design of TALENs requires precise DNA‐binding modules to recognize the target DNA sequences, which may involve complex molecular cloning and sequencing operations. Second, despite the high specificity of TALENs, they can still potentially cause DNA cutting at off‐target sites, leading to unintended effects. Finally, the expression of TALENs may have toxicity to certain cell types, especially when expressed at high concentrations or over long periods.^83^, ^84^

### 3.4 Cell therapy

Cell therapy is an evolving field of research in the application for cardiomyopathy, and the most widely used cell therapy in cardiomyopathy at present is induced pluripotent stem cell (iPSC) therapy. iPSCs are reprogrammed cells analogous to embryonic stem cells. They have the capacity for self‐renewal and multidirectional differentiation.^85^ Genome editing would take place in ex vivo iPSCs, which after editing or differentiating into the desired tissue type, and then be transplanted back into the patient.^86^ Ong et al.^87^ suggested the transplantation of human iPSC‐derived cardiomyocytes (hiPSC‐CMs) could improve LV function and attenuate cardiac remodeling in an acute mouse myocardial infarction (MI) model. However, nonfatal ventricular tachycardia may occur in the process of transplantation. Thus, the prevention of arrhythmogenesis is one of the next areas of study for hiPSC‐CMs.^88^ Moreover, the exosomes secreted from the hiPSC‐CMs exert protective effects to salvage the injured neighboring cells by transferring the endogenous molecules.^89^ Tachibana et al.^90^ demonstrated that induced cardiomyocytes (iCMs) exhibited superior efficacy in salvaging injured myocardium compared with undifferentiated stem cells. This enhanced outcome was attributed to the paracrine effects exerted by and iCMs.^90^ The integration of iPSCs with genome editing technology is beneficial to expand the understanding of the gene's biological function and the pathological implications of genetic variants in cardiomyopathies. The CRISPR/Cas9 technology has been proven to be particularly useful for editing iPSC.^75^, ^91^

## 4 HYPERTROPHIC CARDIOMYOPATHY

HCM stands as the prevalent primary cardiomyopathy and can cause exertional dyspnea, presyncope, atypical chest pain, heart failure, and SCD in adults under 50 years of age, especially among young athletes.^92^, ^93^, ^94^ Asymmetric septal hypertrophy represents a frequently observed characteristic of HCM, which is subsequently accompanied by contractile dysfunction, myocardial fibrosis, and arrhythmias. HCM is a genetically diverse disorder that exhibits heterogeneity, often attributed to mutations in sarcomeric genes. These genetic mutations give rise to LV hypertrophy, fibrosis, hypercontractility, and decreased compliance. HCM follows an autosomal dominant inheritance pattern, meaning it is passed on to offspring with a Mendelian frequency of 50%.^95^, ^96^, ^97^ Many studies have shown that family screening in HCM contributed to the prediction of morbidity.^98^, ^99^

### 4.1 Pathogenesis and disease‐causing genes

**FIGURE 3:**
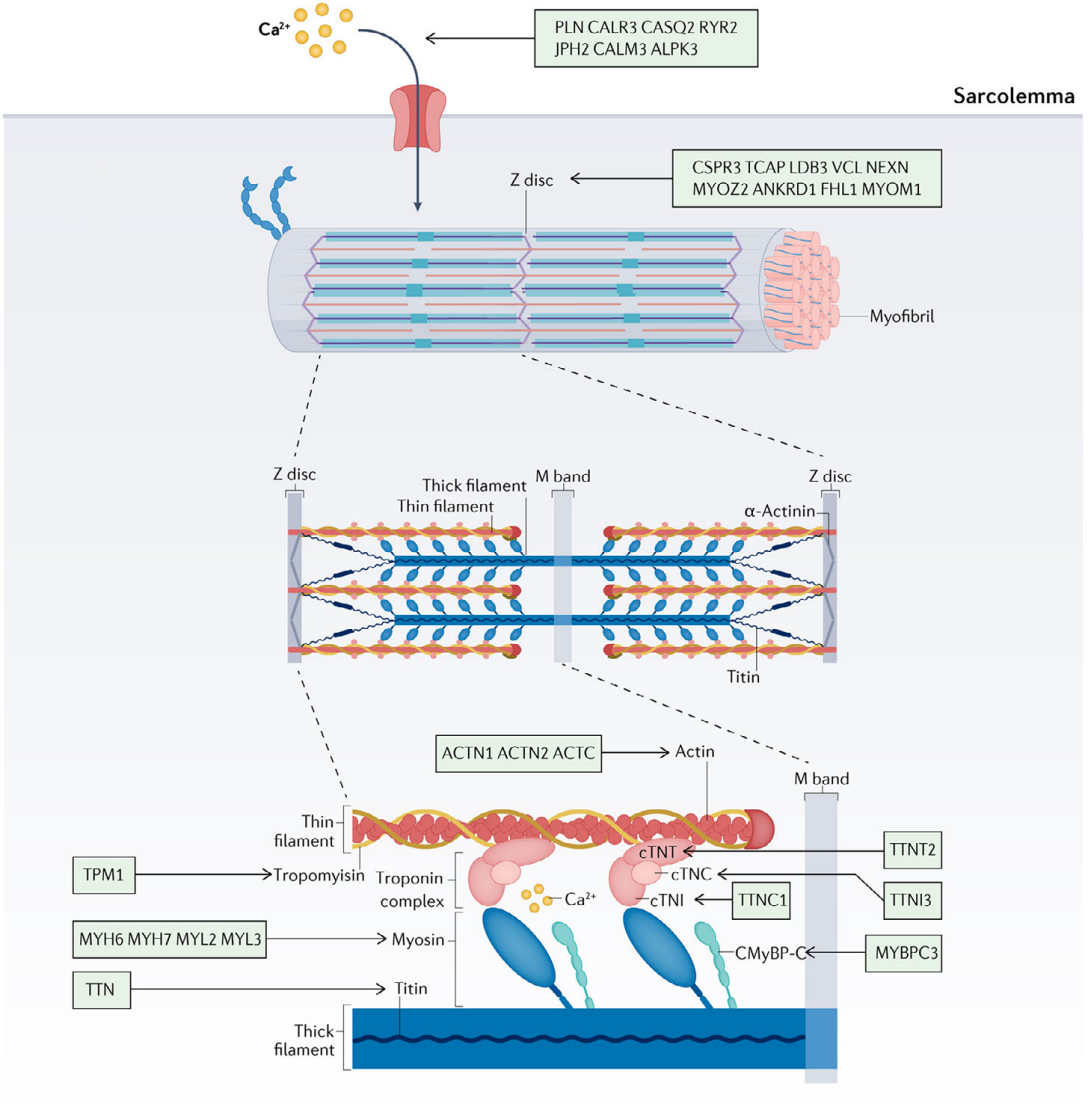
Summary of genes associated with HCM and their function. The sarcomere is the most basic contractile unit in cardiac myocytes. Mutations in genes encoding sarcomeric proteins give rise to cardiomyopathies. The motor domains of myosin filaments (encoded by *MYH6*, etc.) cyclic interact with actin filaments (encoded by *ACTN1*, etc.) and form cross‐bridges, generating force and movement by using ATP, which influenced by intracellular Ca^2+^ concentration, PLN, and so on can regulate Ca^2+^ influx. Cardiac troponin is the protein that plays a major regulatory role in the contractile machinery. There are three subunits: troponin T (cTnT), binding to tropomyosin (encoded by *TPM1*), troponin I (*cTnI*), regulating actin ATPase activity, and troponin C (cTnC), binding to calcium, which separately encoded by *TNNT2*, and so on. The shortening of the sarcomere is caused by a relative sliding of the actin and myosin filaments. Cross‐linking elements such as Z‐disc (encoded by *CSPR3*, etc.) at the Z‐ and M‐lines hold the sarcomeric structures in the correct place. Titin proteins (encoded by *TTN*) connect the myosin filaments to the Z line leading to muscle relaxation and flexing. Cardiac myosin‐binding protein C, encoded by *MYBPC3*, is an important regulator of cardiomyocyte contraction and relaxation.

The pathogenesis of HCM involves the participation of several genes responsible for encoding sarcomeric proteins, Z‐Disc proteins, and calcium‐handling proteins. The genes associated with HCM are summarized here (Table 1 and Figure 3).^9^, ^10^, ^11^, ^12^ The primary genetic cause of HCM is the presence of dominant pathogenic mutations in genes that encode sarcomere proteins, specifically those involved in thick and thin filament formation.^100^ Numerous clinical genomic studies suggest that mutations in the sarcomere protein gene *MYH7* and *MYBPC3* may cause HCM. Mutations in *MYH7* and *MYBPC3* can lead to altered sarcomere function, which may increase myocardial load due to impaired contractility. Mutation of the *MYBPC3* gene promotes the expression of cMyBP‐C protein and results in excessive contraction of myosin,^101^, ^102^ which induces aberrant cross‐bridge kinetics, resulting in severe myocardial contractile dysfunction and eventually leading to HCM. In a study consisting of 26 HCM patients (11 with MYBPC3 mutations, nine with *MYH7* mutations, and six with no sarcomere mutations, referred to as HCMsmn), it was observed that sarcomere mutations disrupt the energetic expenditure of cardiac contraction.^103^ Furthermore, several studies have proposed that disturbed metabolic signaling and impaired mitochondrial function represent prevalent pathogenic mechanisms in individuals diagnosed with HCM.^104^

**TABLE 1:** Chromosomal loci and disease‐causing genes in HCM and ARVC/D.

Gene	Locus	Protein	Frequency
*HCM‐associated genes*			
Sarcomere HCM			
Giant filament			
*TTN* ^9^	2q31	Titin	Rare
Thick filament			
*MYH7* ^9^	14q11.2‐q12	β‐Myosin heavy chain	25–40%
*MYH6* ^9^	14q11.2‐q12	α‐Myosin heavy chain	Rare
*MYL2* ^9^	12q23‐q24.3	Regulatory myosin light chain	0.5–1%
*MYL3* ^9^	3p21.2‐p21.3	Essential myosin light chain	0.5–1%
Intermediate filament			
*MYBPC3* ^9^	11p11.2	Cardiac myosin‐binding protein C	25–40%
Thin filament			
*TNNT2* ^9^	1q32	Cardiac troponin T	3–5%
*TNNI3* ^9^	19p13.4	Cardiac troponin I	1–5%
*TPM1* ^9^	15q22.1	α‐Tropomyosin	1–5%
*ACTC* ^9^	15q14	α‐Cardiac action	Rare
*TNNC1* ^9^	3p21.1	Cardiac troponin C	Rare
*MYOM1* ^9^	18p11.31	Myomesin 1	Rare
*MYOZ2* ^9^	4q26‐q27	Myozenin 2	Rare
Z‐Disc HCM			
*CSRP3* ^12^	11p15.1	Muscle LIM protein	Rare
*TCAP* ^12^	17q12‐q21.1	Telethonin	Rare
*LDB3* ^12^	10q22.2‐q23.3	LIM binding domain 3	Rare
*ACTN1* ^12^	14q24.1	α‐Actinin 1	Rare
*ACTN2* ^12^	1q42‐q43	α‐Actinin 2	Rare
*VCL* ^12^	10q22.1‐q23	Vinculin/metavinculin	Rare
*ANKRD1* ^12^	10q23.31	Cardiac ankyrin repeat protein	Rare
*FHL1* ^12^	Xq26.3	Four‐and‐a‐half LIM domains 1	Rare
*NEXN* ^12^	1p31.1	Nexilin F‐actin binding protein	Rare
*BAG3* ^12^	10q26.11	BAG cochaperone 3	Rare
Calcium‐handling HCM			
*PLN* ^12^	6q22.1	Phospholamban	Rare
*CALR3* ^12^	19p13.11	Calreticulin 3	Rare
*CASQ2* ^10^	1p13.3‐p11	Calsequestrin	Rare
*RYR2* ^10^	1q42.1‐q43	Ryanodine receptor 2	Rare
*JPH2* ^10^	20q13.12	Junctophilin 2	Rare
*CALM3* ^10^	19q13.2–q13.3	Calmodulin 3	Rare
*ALPK3* ^11^	15q25.2	Alpha‐protein kinase 3	Rare
*ARVC/D‐associated genes*			
Desmosomal			
*PKP2* ^105^	12p11	Plakophilin‐2	20–46%
*DSP* ^106^	6p24	Desmoplakin	3–15%
*DSG2* ^106^	18q12.1	Desmoglein‐2	3–20%
*DSC2* ^106^	18q12.1	Desmocollin‐2	1–15%
*JUP* ^107^	17q21	Plakoglobin	Rare
Nondesmosomal			
*CTNNA3* ^107^	10q21.3	αT catenin	Rare
*CDH2* ^108^	18q12.1	Cadherin‐2	Rare
*PLN* ^109^	6q22.31	Phospholamban	0–4%
*TMEM43* ^109^	3p25.1	Transmenbrance protein 43	0–2%
*TGFB3* ^109^	14q24.3	Transforming growth factor beta 3	Rare
*SCN5A* ^109^	3p22.2	Na_v_1.5	2%
*LMNA* ^109^	1q22	Lamin A/C	0–4%
*FLNC* ^109^	7q32.1	Filamin C	0–3%
*DES* ^109^	2q35	Desmin	Rare
*RYR2* ^109^	1q43	Ryanodine receptor‐2	Rare
*TJP1* ^109^	15q13.1	Zona occludens‐1	Rare
*TTN* ^109^	2q31.2	Titin	0–10%

In a multicenter multinational study of 358 patients with consecutive genotyped HCM, *MYH7* (*n* = 53) and *MYBPC3* (*n* = 75) were identified as the most common genes, respectively, at 33.1 and 47% of gene positive patients.^110^ Out of more than 1400 reported pathogenic variants, *MYH7* and *MYBPC3* genes account for approximately 70–80% of the causal genes in HCM.^111^ Homozygous or compound heterozygous frameshift mutations in *MYBPC3* can be the underlying cause of neonatal HCM, which promptly progresses to systolic heart failure and usually results in mortality within the first year of life.^112^ According to a comprehensive study conducted in Iceland, it has been found that a founder mutation of *MYBPC3*, which originated more than 550 years ago, is the primary cause of HCM in the country. Additionally, the genotype of this mutation can influence the prognosis of individuals with HCM. Recent research has indicated that the *MYBPC3* c.927‐2A:G mutation is linked to lower rates of adverse events (AEs), but it is associated with earlier cardiovascular mortality.^113^ Montag et al.^114^ applied TALEN‐mediated genome editing to create a porcine model that demonstrated characteristics of HCM by introducing the HCM‐point mutation R723G into the *MYH7* gene.

Furthermore, changes in the promoter region of *MYH7* could be linked to the risk of developing HCM, and the patients harboring likely pathogenic or pathogenic variations in the *MYH7* gene exhibited a higher incidence of developing new‐onset atrial fibrillation.^115^, ^116^, ^117^ Apart from the mutations with *MYBPC3* and *MYH7*, the variants in other genes encoding sarcomeric proteins also cause HCM. Compound *DSG2/DSC2/MYH6* mutations were found in an athlete, and these variants determined a mild hypertrophic phenotype associated with both ventricular tachyarrhythmias and atrioventricular block.^118^ Mutations in troponin T, I, and Tm represent less than 10% of diagnosed cases of HCM. Pua et al.^119^ demonstrated that Chinese HCM patients often have low penetrance risk alleles in *TNNT2* or *TNNI3* compared with white patients.^120^

In general, patients with sarcomere mutations receive an earlier diagnosis and exhibit more severe hypertrophy compared with those without mutations.^121^, ^122^ Differences in disease onset may be due to the gene‐specific severity of cardiac abnormalities.^103^

Recent research indicates that mutations in genes encoding the proteins forming the cardiac Z‐disc, located adjacent to each other, are prevalent in both HCM and DCM. The proteins at the Z‐disc facilitate the anchoring of thin filaments in neighboring cardiac sarcomeres, serving as a mechanical integration site for transducing sarcomere force generation through the myofilaments.^123^ Some genes encoding Z‐disc proteins have been demonstrated to result in the onset of HCM (Table 2). Wang et al.^124^ demonstrated that mutations in *NEXNs*, a recently identified member of the Z‐disc gene family, may be associated with HCM. Gallego‐Delgado et al.^125^ suggested that HCM caused by *FHL1* mutations has a very aggressive course and poor prognosis.

**TABLE 2:** Major clinical trials of therapeutic strategies on cardiomyopathy.

Intervention/treatment	Targets	Mechanisms	ClinicalTrials.gov ID	Study population	Primary endpoint	Phase	References
Mavacamten	β‐Myosin heavy chain	Stabilizes the super relaxed state	NCT03470545	HCM	1.5 mL/kg per min or greater increase in pVO_2_ and at least one NYHA class reduction or a 3·0 mL/kg per min or greater pVO_2_ increase without NYHA class worsening	Phase III	^126^
			NCT04349072	oHCM	The proportion of patients proceeding with SRT or remaining guideline‐eligible at 32 weeks in both treatment groups	Phase III	^127^
			NCT05174416	Chinese oHCM	Change in Valsalva LVOT peak gradient	Phase III	^128^
Aficamten	Cardiac myosin	Decreases the number of active actin‐myosin cross‐bridges	NCT0518681	HCM	Adverse cardiac events	Phase III	^129^
AAV1/SERCA2a	*SERCA2a*	Corrects abnormal Sarcoplasmic reticulum Ca^2^+–ATPase activity	NCT01643330	Heart failure	Cardiovascular hospitalizations and time to terminal events	Phase II	^56^
Belantamab mafodotin	B‐cell maturation antigen	Induces apoptosis	NCT04484623	Relapsed or refractory myeloma	Progression‐free survival	Phase III	^130^
			NCT03525678	Relapsed or refractory myeloma	The proportion of randomly assigned patients in the intention‐to‐treat population who achieved an overall response	Phase II	^131^
			NCT04162210	Multiple myeloma	Progression‐free survival in all patients who were randomly allocated	Phase III	^132^
rAAVrh74.MHCK7.micro‐dystrophin	*DMD*	Transfers microdystrophin gene	NCT03375164	DMD	Safety	Phase I/IIa	^133^
Givinostat	Histone deacetylase	Inhibits histone deacetylase	NCT02851797	DMD	Results of the four‐stair climb assessment	Phase III	^134^
Eteplirsen	Exon 51	Exon skipping to restore the dystrophin open reading frame	NCT0229655552	DMD	Change in 6‐min walk test	Phase III	^135^
Bromocriptine	Prolactin	Inhibits the production of antiangiogenic cleaved prolactin fragment	NCT00998556	PPCM	LVEF change (delta)	Multicenter randomized study	^136^
Inotersen	TTR	Inhibits hepatic production of transthyretin	NCT01737398	Cardiac amyloidosis	Modified neuropathy impairment score+7	Phase III	^137^
VEGF165	VEGF receptors	Induces angiogenesis in ischemic tissues	NCT00744315	Refractory angina	Worsening of rest ischemia scores	Phase I/II	^138^
AdVEGF121	VEGF receptors	Mediates the generation of new blood vessels and reverse coronary ischemia	NCT01174095	Late‐stage, diffuse coronary artery disease	Death	Phase I	^139^

Abbreviations: pVO_2_, peak oxygen consumption; oHCM, obstructive hypertrophic cardiomyopathy; SRT, septal reduction therapies; NYHA, New York Heart Association; LVOT, left ventricular out flow tract; nHCM, nonobstructive hypertrophic cardiomyopathy; KCCQ‐CSS, Kansas City Cardiomyopathy Questionnaire—Clinical Summary Score; rAAVrh74, recombinant adeno‐associated virus serotype rh74; DMD, Duchenne muscular dystrophy; PPCM, peripartum cardiomyopathy; LVEF, left ventricular ejection fraction; TTR, transthyretin; VEGF, vascular endothelial growth factor.

An increasing number of studies have indicated that the alteration of Ca^2+^ homeostasis may be associated with prohypertrophic remodeling.^140^ Therefore, apart from sarcomeric HCM and Z‐disc HCM, genes encoding Ca^2+^‐handling or Ca^2+^‐regulatory proteins (PLN, CALR3, CALM3,^141^ Junctophilin 2 [JPH2],^142^, ^143^ CASQ2, RYR2) have been proposed as potential causal genes for HCM,^144^ further indicating the heterogeneity in the etiology and pathogenesis of cardiomyopathy. The *JPH2* mutant gene has been considered one of the causal genes in familial HCM.^145^ Matsushita et al.^143^ demonstrated that the *JPH2* gene mutation could lead to the diagnosis of HCM. Vanninen et al.^146^ proposed that the heterozygous *JPH2* p.(Thr161Lys) variant could lead to heart failure in atypical HCM.

### 4.2 Therapeutic interventions

The treatment strategies for HCM include pharmacological therapy, surgical treatment, and emerging gene therapy approaches. The goals of HCM treatment are to reduce mortality, improve cardiac function, alleviate clinical symptoms, and slow down the progression of the disease.

#### 4.2.1 Pharmacological treatment

##### Traditional pharmacological treatment

Traditional medications mainly include β‐blockers, nondihydropyridine calcium channel blockers, late sodium current inhibitors, angiotensin receptor blockers (ARBs), and the antiarrhythmic drug disopyramide. The traditional pharmacological treatment options for HCM are limited to nonspecific drugs that can alleviate symptoms to varying degrees but do not truly slow the progression of the disease. In recent years, new compounds that directly address myocardial hypercontraction and energy changes have been developed.^147^

##### Mavacamten

Mavacamten (MYK‐461) is a small molecule allosteric inhibitor of cardiac myosin that inhibits the excessive formation of myosin‐actin cross‐bridges at the sarcomere level.^148^ Mavacamten has successfully advanced to Phase III clinical trials. The EXPLORER‐HCM study is a 30‐week, double‐blind, placebo‐controlled RCT, showing that its primary composite endpoint [increase in peak oxygen consumption (pVO_2_) by ≥1.5 mL kg^−1^ min^−1^, along with an improvement of ≥1 in New York Heart Association (NYHA) functional classification; or an improvement in pVO_2_ by ≥3.0 mL kg^−1^ min^−1^ without a decline in NYHA classification] achieved significant statistical difference (*p* = 0.0005). It also confirmed that Mavacamten can significantly improve patients’ exercise capacity (*p* = 0.0006), LVOTO (*p* < 0.0001), NYHA functional classification (*p* < 0.0001), and health status (*p* < 0.0001). The proportion of patients achieving complete resolution in both groups was 27% and less than 1%, respectively.^126^ The study demonstrated that Mavacamten, as a specific treatment for HCM, can bring about significant improvements in hemodynamics, cardiac function, and quality of life scores with clinical significance. Moreover, in terms of safety and tolerability, the results for the treatment group were similar to those of the placebo patients, with adverse reactions during treatment usually being mild. Another Phase III study, the VALOR‐HCM study, aimed to assess whether the treatment with Mavacamten could reduce the need for septal reduction therapies (SRTs) in patients with obstructive HCM.^127^ The study demonstrated that the addition of Mavacamten treatment for 16 weeks on top of maximally tolerated background medical therapy significantly reduced the proportion of patients who still met the guidelines for SRT indications (*p* < 0.0001), which may also be beneficial for improving the quality of life in patients with severe symptomatic disease.

##### Aficamten

Aficamten (CK‐274/CK‐3773274) is a novel selective small molecule inhibitor of cardiac myosin. The drug has demonstrated safety and tolerability in healthy populations, with no serious AEs observed during treatment, and no clinically meaningful changes in vital signs, electrocardiograms, or laboratory test results.^149^ Based on this, the drug has initiated Phase III (SEQUOIA‐HCM) clinical trials.^129^ Preliminary results from the Phase II study indicate that Aficamten can significantly reduce LV outflow tract gradient and NT‐proBNP levels.

Furthermore, potential pharmaceutical interventions might exist for mitigating HCM resulting from *MYBPC3* mutations. In their study, Singh et al.^116^ observed that administering rapamycin at a dose of 2.24 mg/kgxd or implementing a 40% caloric restriction for a duration of 9 weeks enhanced Akt–mTORC1 signaling, partially reinstated autophagic flux, and successfully rescued cardiomyopathy in *Mybpc3*‐targeted KI mice. These findings serve as evidence that autophagy modulators can effectively impede the progression of cardiomyopathy in KI mice.^150^

#### 4.2.2 Surgical treatment

The surgical treatments for HCM mainly include ventricular septal myectomy (VSM) and alcohol septal ablation (ASA). VSM involves the surgical removal of a portion of the hypertrophied myocardium from the interventricular septum to reduce or eliminate LVOTO, thereby improving cardiac function and symptoms. This is an effective treatment for obstructive HCM, especially for patients with ineffective medical treatment or severe symptoms. ASA, on the other hand, involves the percutaneous injection of alcohol into the septal branches of the coronary artery, causing local MI, reducing the thickness of the interventricular septum, and thus alleviating LVOTO.^151^ This method is suitable for patients with high surgical risk or contraindications to surgery.

#### 4.2.3 Gene therapy

##### Myosin‐binding protein C3

Gene therapy approaches aimed at addressing HCM primarily concentrate on targeting *MYBPC3* mutations, which offer promising potential as a successful translation from laboratory research to practical clinical interventions. This focus is justified by the fact that *MYBPC3* mutations are the most frequently observed genetic abnormalities in patients with HCM.^152^ Merkulov et al.^153^ carried out gene transfer by using a specific lentiviral vector, which increased the expression level of the *MYBPC3* gene in HCM mice, thereby restoring the abnormal dynamics of myocardial cross‐bridges, improving myocardial contractile function, delaying or reversing the pathogenesis of cardiac hypertrophy and myocardial fibrosis. On account of most *MYBPC3* mutations leading to cMyBP‐C haploinsufficiency, the potential strategy to treat *MYBPC3*‐caused HCM is the introduction of wild‐type *MYBPC3* cDNA into abnormal cardiomyocytes.^154^ Recently, Prondzynski et al.^155^ introduced the full‐length *MYBPC3* cDNA into abnormal cardiomyocytes induced by pluripotent stem cells from HCM patients caused by mutation of the *MYBPC3* gene, hence increasing the cMyBP‐C expression level and successfully improving cardiac hypertrophy. Gedicke‐Hornung et al.^66^ transduced the AON, which mediates exon skipping, into cardiomyocytes of neonatal mice by exon skipping therapy and inhibiting *MYBPC3* gene mutation. The expression of abnormal transcriptional mRNA increased the expression of the deleted exon and successfully inhibited the progression of cardiac hypertrophy. Recent studies have found that in the HCM model of *MYBPC3* gene mutation, the entire *MYBPC3* mRNA mutation can be repaired by PTMs (pretrans‐splicing molecule); theoretically, 40–60% of HCM patients can be cured.^156^, ^157^, ^158^

In addition, the full‐length and functional repair of the cMyBP‐C protein can be achieved using trans‐splicing technology, enabling a faster and more accessible approach. Mearini et al.^159^ showed the feasibility of employing the 5′‐trans‐splicing strategy to repair *Mybpc3* mRNA in a mouse model of HCM with a *Mybpc3* mutation. The repaired *Mybpc3* mRNA constituted approximately 66% of the overall *Mybpc3* transcripts present in cardiac myocytes. Moreover, they correctly repaired the cMyBP‐C protein incorporated into the sarcomere in cardiac myocytes. Mearini et al.^159^ also found that a single systemic administration of AAV9–*Mybpc3* in mice could ameliorate cardiomyopathy by increasing *Mybpc3* mRNA and cMyBP‐C protein levels in a dose‐dependent manner. Moreover, Li et al.^160^ also indicated that AAV9 gene transfer of cMyBP‐C N‐terminal domains that contained domains C0C2 prevented the development of cardiac hypertrophy and dysfunction in cMyBP‐C‐deficient mice, which genetically mimic this human cardiomyopathy.

##### Beta‐myosin heavy chain

Mutations in the *MYH7* R403Q in HCM patients result in a particularly severe cardiomyopathy characterized by progressive myocardial dysfunction. In a recent study, Anderson et al.^161^ identified that the *MYH7* sequence could be targeted, referenced, or alternated selectively by three SNPs in MYH7 or ASOs libraries and suggested that SNP‐targeting ASOs are a promising therapeutic strategy for treating cardiomyopathy. Yue et al.^162^ discovered that CASAAV (CRISPR/Cas9–AAV9‐based somatic mutagenesis) technique successfully silenced Myh6 and Myh7 in cardiomyocytes. Bu et al.^163^ recently discovered ventricle myosin heavy chain like (vmhcl) as the zebrafish equivalent of human *MYH7* and subsequently showcased the therapeutic advantages of inhibiting mTOR and mitogen‐activated protein kinase (MAPK) pathways in vmhcl homozygous mutants.

##### Myosin heavy chain 6

Heterozygous MHC^403/+^ mice express the R403Q mutation in *Myosin heavy chain 6* (*Myh6)*, which causes changes in sarcomere function, including increased actomyosin sliding rate and hydrolysis of ATP.^148^, ^164^ Jiang et al.^64^ delayed the development of HCM by introducing a specific RNA inhibitor into the HCM animal model induced by the new mutation of the *MYH6* gene via the viral vector AAV9 (AAV‐9–cTnT–EGFP–RNAi).^165^, ^166^ In their research, Ma et al.^167^ developed a novel adenine base editor platform known as ABEmax‐NG system, which exhibited the capability to effectively rectify a pathogenic *Myh6* mutation through embryonic gene correction in mouse embryos. This correction mechanism proved instrumental in averting the progression of HCM.^167^

##### Chromatin remodeling protein

Han et al.^168^ discovered that chromatin remodeling protein (*BRG1*) plays a pivotal role in governing early‐age myocardial development, differentiation, and gene expression. The *MYH7* in healthy adult hearts can be cleaved by cardiac‐specific antisense transcription to generate a batch of lncRNA molecules called *Mhrt* (Myosin Heavy Chain Associated RNA Transcripts); *Mhrt* can antagonize the effect of *Brg1* on chromatin. A pressure load stimulus activates *Brg1*, resulting in cardiac hypertrophy. In hiPSC‐CMs, *MYH7* could be decreased, but *MYH6* could be increased by inhibition of *BRG1*, which suggests *BRG1* assumes a regulatory role in the pathological imbalance of the two myosin heavy chain isoforms in individuals with HCM.^169^ The team found a lncRNA that blocks pathological cardiac hypertrophy by antagonizing the effects of *Brg1*. Moreover, when subjected to adverse stimulus in the myocardium, the activated *Brg1* will form a BRG1–Hdac–Parp complex, binding to the *Mhrt* promoter and inhibiting *Mhrt* transcription, forming a complete cardioprotective feedback loop.^168^ The discovery of the HCM regulatory protein BRG1 and the myocardial protection sequence *Mhrt* gene promotes the development of biochemical markers, enabling early identification and targeted treatment of HCM through designing novel, targeted drugs.

##### Sarcoplasmic reticulum Ca^2+^‐ATPase 2a

A decreased ratio of sarcoplasmic reticulum Ca^2+^‐ATPase 2a (Serca2a) to phosphoprotein (*PLB*) can affect the activity of the sarcomere and reticulum calcium pump, resulting in the myocardial inability to maintain normal diastolic function.^170^ Pena et al.^171^ indicated that the *Serca2a/PLB* ratio can be improved by upregulating *Serca2a* gene expression using viral vectors, which may enhance myocardial cell diastolic function and delay cardiac hypertrophy and myocardial fibrosis. Phospholamban (PLN) is an inhibitor of cardiac muscle Serca2a in the unphosphorylated state. Gaffin et al.^172^ demonstrated that the enduring mitigation of familial HCM, resulting from mutations in genes encoding thin filament protein and tropomyosin, could potentially be achieved through the modulation of a calcium cycling protein, specifically the deletion of the *PLN* gene. The CUPID study is the first clinical study to use gene transfer technology for individual therapy. In this study, the patients with severe heart failure received a single IC infusion of AAV1/*SERCA2a*. A CUPID Phase II study included 39 patients with heart failure compared with the placebo group. The high‐dose group significantly decreased cardiovascular event rate (HR = 0.12, *p* = 0.003) and hospitalization (0.4 d vs. 4.5 d, *p* = 0.05) at 1 year.^173^ After 3 years, the high‐dose group had an 82% reduction in cardiovascular events compared with the placebo group (*p* = 0.048). Cardiac biopsy was performed on three high‐dose patients to confirm the presence of therapeutic genes.^55^ Despite the same treatment, in the CUPID IIb phase study with more subjects, the 1‐year follow‐up showed no improvement in clinical outcomes or ejection fraction in patients with heart failure.^56^ Nevertheless, the study provides a perspective for the future use of adenoviruses in the gene therapy for cardiomyopathy.

##### Protein kinase AMP‐activated noncatalytic subunit gamma 2

Mutations occurring in the protein kinase AMP‐activated noncatalytic subunit gamma 2 (*PRKAG2*) gene, responsible for encoding the γ2‐subunit of AMPK, give rise toHCM and familial Wolff–Parkinson–White syndrome.^174^, ^175^ Ben Jehuda et al.^176^ generated iPSC‐HCMs with *PRKAG2* mutations, which demonstrated both functional and structural abnormalities in cardiac myocytes, indicative of HCM. The researchers successfully employed CRISPR technology to rectify the mutation in the patient's iPSCs, thereby eliminating the HCM‐associated characteristics.^176^ Zhan et al.^177^ utilized CRISPR–Cas9‐mediated genome editing to rectify the R302Q mutation in hiPS‐CMs. These cardiomyocytes harbored a heterozygous missense mutation (c.905G>A, R302Q) in the *PRKAG2* gene.^177^

##### Long noncoding RNAs

Apart from the therapy targeting causal genes, Mosqueira et al.^178^ proposed potential diagnostic biomarkers and therapeutic targets. They used CRISPR/Cas9 to produce isogenic sets of C9123T–MYH7 (R453C–bMHC) mutants in hiPSC‐CM. The discovery of previously unidentified lncRNAs and potential gene modifiers opens up opportunities for gaining fresh insights into molecular mechanisms and functional aspects through techniques such as knockout, overexpression, and pathway analysis.^178^

## 5 ARRHYTHMOGENIC RIGHT VENTRICULAR CARDIOMYOPATHY

ARVC is an uncommon hereditary cardiac disorder that ranks among the leading causes of SCD in young individuals.^107^, ^179^ Therefore, professional societies in Europe and North America advise individuals with ARVC to refrain from engaging in high‐intensity exercise.^180^, ^181^, ^182^

### 5.1 Pathogenesis and disease‐causing genes

Alterations in genes encoding desmosomal proteins or proteins that interact with desmosomal proteins have been identified as a disease‐causing factor in ARVC, which contribute to the occurrence of the disease in more than 50% of individuals diagnosed with classical ARVC.^183^, ^184^ The most common defective ARVC genes have been discovered in genes encoding desmosomal proteins, including *JUP, PKP2, DSP, DSG2*, and *DSC2*,^14^, ^15^, ^16^ in which 87% of the genetic variants were found within the five genes.^105^, ^106^ Nondesmosomal pathogenic variants have been described in *DES*, *LMNA*, *SCN5A*, *CDH2*, *CTNNA3*, *FLNC*, *PLN*, *TGFβ3*, *TMEM43*, *RYR2*, *TJP1*, and *TTN* ^106^, ^107^, ^109^, ^179^, ^180^ (Table 1). Brun et al.^185^ identified two unique FLNCtv variants in two families causing ARVC. Currently available therapeutic tools include antiarrhythmic drugs, catheter ablation, and implantable cardioverter defibrillators.^186^ In accordance with the desmosomal model, recent studies have shown that exercise often triggers SCD in ARVC. Gene therapy facilitated by recombinant AAV (rAAV) offers a compelling approach for precise, targeted interventions, holding the potential to revolutionize treatment strategies for patients with ARVC.^187^

The propensity of ARVC to cause arrhythmias is intricate, involving multiple mechanisms. The abnormal signal transduction and the establishment of macro‐reentry circuits, triggered by the deposition of fibrofatty scar tissue, can result in the formation of malignant ventricular arrhythmias. Moreover, the intricate interplay among desmosomes, voltage‐gated sodium channels, and gap junction proteins within the intercalated disc is associated with the disruption of normal cell signaling, further promoting arrhythmogenesis.^24^

### 5.2 Therapeutic interventions

Although ARVC significantly contributes to SCD among young individuals, there is currently no effective method to reverse its progression. Consequently, recent therapeutic approaches have shifted their focus toward inhibiting or delaying the advancement of ARVC. Numerous preclinical studies have been conducted in an effort to uncover additional evidence that could aid in the management of this condition.

#### 5.2.1 Pharmacological treatment

The data related to drug treatment for ARVC are relatively scarce. In clinical practice, some antiarrhythmic drugs are commonly recommended to slow down the progression of the disease.

#### 5.2.2 Surgical treatment

When using ICD as primary prevention, it is necessary to weigh the absolute risk of SCD and device‐related complications, including inappropriate shocks and infections.^188^ For patients with ARVC, a combination of endocardial and epicardial ablation is usually required. Some researchers suggest starting with endocardial ablation followed by epicardial ablation. In experienced centers, catheter ablation is an important adjunctive treatment for ARVC patients with ventricular arrhythmias.^189^ Patients who have undergone catheter ablation and meet the indications can still receive an ICD implant. The main indication for heart transplantation is severe right ventricular dysfunction, and patients with refractory ventricular arrhythmias can also be considered for heart transplantation.^190^

#### 5.2.3 Gene therapy

##### Plakophilin‐2

PKP2, a key desmosome component, plays a crucial role in cell–cell adhesion. Mutations in the human *PKP2* gene are linked to the severe, life‐threatening ARVC.^191^ Preliminary research into gene therapy for *PKP2* has utilized rAAV as a vector. Wu et al.^192^ demonstrated that a single administration of AAV9:*PKP2* gene delivery effectively prevents the onset of ARVC by restoring the integrity of desmosomal and gap junctional cellular structures. This intervention not only maintains or enhances LVEF but also arrests or reverses right ventricular dilation. Furthermore, it mitigates the frequency and severity of ventricular arrhythmias and averts detrimental fibrotic remodeling, showcasing the therapeutic potential of this targeted gene therapy approach.^192^ van Opbergen et al.^193^ determined that the delivery of PKP2a via AAVrh.74–PKP2a significantly enhanced survival rates in the *PKP2*–cKO murine model, which features cardiac‐specific, tamoxifen‐inducible *PKP2* deletion. The therapeutic advantage was pronounced in mice that received AAVrh.74–PKP2a postonset of the disease. Echocardiographic evaluation disclosed that AAVrh.74–PKP2a efficaciously averted dilation of the right ventricle, halted the progressive decline in LV function, and alleviated the severity of arrhythmias. The study presented robust preclinical evidence supporting the candidacy of AAVrh.74–PKP2a (RP‐A601) as a promising therapeutic intervention for *PKP2*‐associated ARVC, efficacious in both the incipient and advanced stages of the disease.^193^

##### Phospholamban

*PLN* is a critical regulator of calcium cycling and contractility in the heart. The loss of arginine at position 14 in *PLN* (R14del) is associated with DCM with a high prevalence of ventricular arrhythmias.^194^ Karakikes et al.^195^ derived iPSCs from a patient with the *PLN* R14del mutation and successfully differentiated these into cardiomyocytes (iPSC‐CMs). Their research revealed that gene correction employing TALENs effectively mitigates the disease phenotypes associated with the R14del mutation in the iPSC‐CMs.^195^ Dave et al.^196^ employed the CRISPR/Cas9 system in conjunction with a cardiotropic AAV9 to perform in vivo genome editing. Their study successfully demonstrated a reduction in end‐diastolic and stroke volumes, as well as a decreased susceptibility to ventricular tachycardia in young adult mice that express the human *PLN*‐R14del mutation.^196^ This preclinical research presents encouraging, potentially translatable methods for the detection and therapeutic modulation of the arrhythmogenic phenotype in individuals with *PLN*‐R14del disease and may also be applicable to other inherited cardiomyopathies.

##### Desmoglein‐2

DSG2, a protein encoded by the *DSG2* gene, is integral to the desmosomal complex that upholds tissue integrity, particularly within the cardiac muscle. Genetic alterations in the *DSG2* gene are known to precipitate arrhythmogenic cardiomyopathy, a condition predominantly associated with the Japanese variant of ARVC.^197^, ^198^ Shiba et al.^199^ generated iPSC from a patient carrying the *DSG2* (c.C355T, p.R119X) mutation (R119X‐iPSC). They successfully heterozygously corrected the mutated *DSG2* gene locus to a normal allele using HDR, resulting in HDR‐iPSCs. In the cardiomyocytes derived from these HDR‐iPSC, the previously observed phenotypes, including abnormal desmosome protein deposition and disrupted intercalated disk structures, were notably restored.^199^

## 6 DILATED CARDIOMYOPATHY

DCM can be an end‐stage form and a common feature of several known causes (e.g., hypertension, ischemia, diabetes, etc.). Nevertheless, DCM is usually associated with a genetic predisposition caused by specific gene mutations, often inherited in an autosomal dominant pattern. Patients with DCM may have multiple affected members within their family, and risks can be identified through genetic counseling and testing. In addition, certain individuals exhibit clinical symptoms such as enlarged ventricular cavities and reduced contractility even without a definitive diagnosis of an underlying primary disease.

DCM is characterized by ventricular enlargement and systolic dysfunction.^200^ Although the left ventricle (LV) quality in DCM is usually significantly increased, the LV wall thickness is reasonable compared with HCM.^3^ It is estimated that the incidence of primary DCM has exceeded one out of 250, and it has a trend of increasing year by year.^201^

### 6.1 Pathogenesis and disease‐causing genes

**FIGURE 4:**
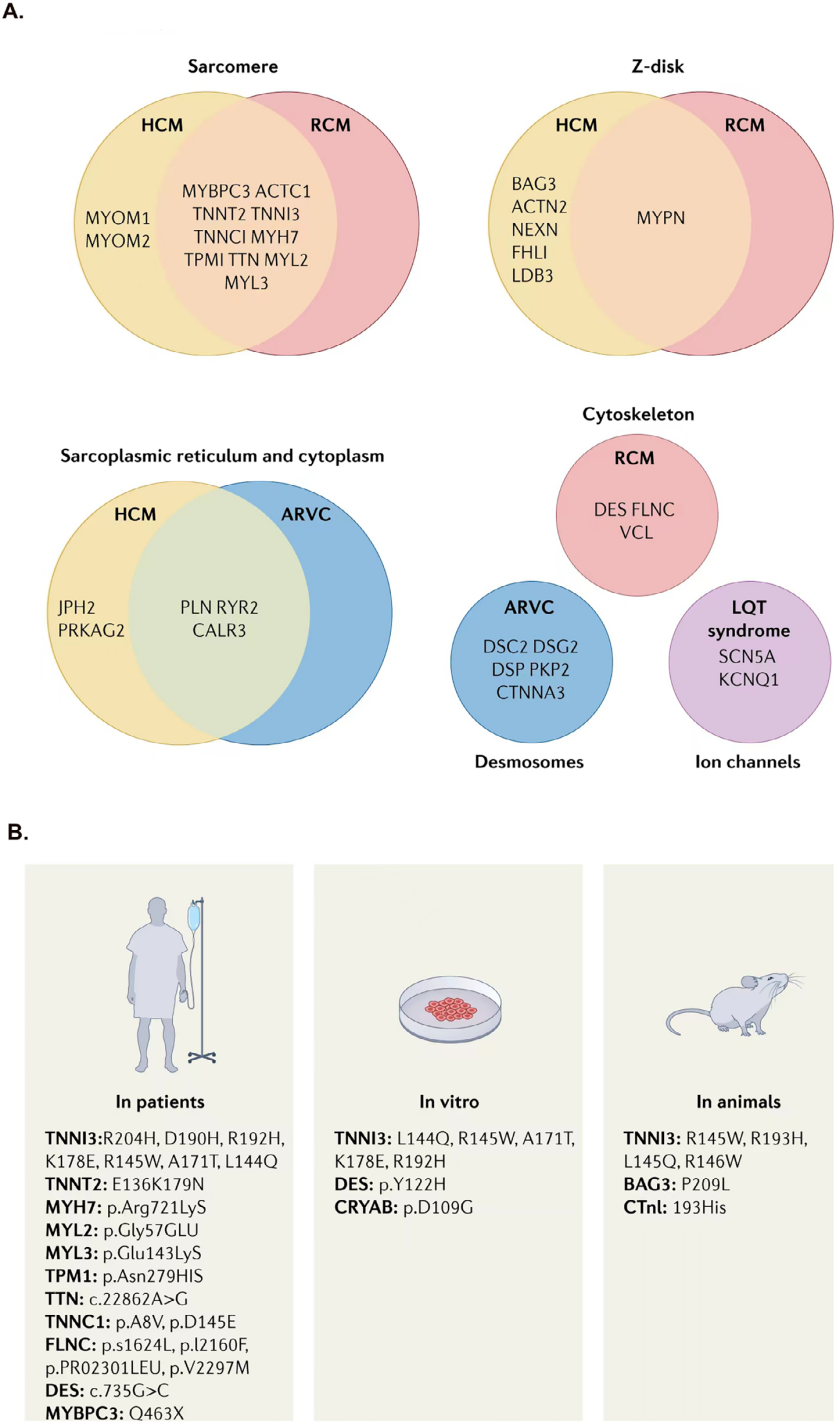
The causal genes in primary cardiomyopathies. (A) The shared genes between DCM and other cardiomyopathies. (B) The different mutations associated with RCM in patients, in vitro, and in animals. The different mutations have been found in patients, in vitro, and in animals. In different species, recent studies have found that mutations at different sites of the same gene can lead to RCM.

In about 35% of patients with DCM, genetic mutations can be identified, which usually involve genes encoding ion channels, cytoskeletal, sarcomere, and nuclear envelope proteins.^202^ Disease‐causing mutations have been identified in more than 50 genes,^13^ including *TTN, DSP, MYH7, BAG3, TNNT2, TNNC1, PLN, ACTC1, NEXN, TPM1, VCL, LMNA, MYBPC3, ABCC9, ACTN2, ANKRD1, CAV3, CHRM2, CRYAB, DES, DMD, DOLK, DSC2, DSCG2, DTNA, EMD, FHL2, GATAD1, GATA4, GLA, LK, JPH2, JUP, LAMA4, LAMP2,LDB3, MURC, MYH6, MYL2, MYL3, MYLK2, MYOM1, MYOZ2, MYPN, NEBL, PDLIM3, PKP2, PRDM16, PRKAG2, PTNP11, RAF1, RBM2, RIT1, RYR2, TNNI3, RBM20, SCM5A*, and so on.^200^, ^203^ Furthermore, more than 40 genes are known to be associated with DCM, and mutations in these genes could also lead to phenotypes of other types of cardiomyopathies. Although each type of cardiomyopathy has its unique characteristics, there may be a certain degree of overlap in clinical presentation and genetic background. DCM often has shared genes and overlapping phenotypes with other cardiomyopathies (Figure 4A). Significant evidence exists for variants within the top 12 genes, which could potentially account for 17% of the cases observed in the outpatient clinic cohort with DCM.^204^ The high‐evidence DCM genes are recommended for use in clinical practice.^205^

#### 6.1.1 Phospholamban

DCM can be caused by mutations in the gene encoding the cardiac protein PLN. Disease‐causing mutations for familial DCM have been identified in the *PLN* gene. The p.(Arg14del) pathogenic variant (R14del) of the *PLN* gene has been regarded as a common cause of DCM with heart failure. Eijgenraam and colleagues^206^ discovered changes in proteostasis and aggregation of PLN protein as the initial indicators of *PLN*‐R14del‐related DCM, suggesting a novel therapeutic target. Yost et al.^207^ described a missense G>A mutation in exon 1 of the *PLN* gene that changed an amino acid arginine to histidine in a spontaneous canine model of familial DCM. hiPSC‐CMs harboring the R9C *PLN* mutation showed activation of a hypertrophic phenotype and also perturbed the expression of several miRNAs involved in fibrosis, hypertrophy, and cardiac metabolism.^208^

#### 6.1.2 Titin

Recent studies have shown that mutations in the gene encoding giant‐muscle filament titin cause autosomal dominant DCM linked to chromosome 2q31 (CMD1G; MIM 604145).^209^ Truncating variants in the *TTN* gene (*TTN*tv) have been identified as the most common cause of heritable DCM. Yoskovitz et al.^210^ determined the sequences of the gene in an Israeli Arab family. The linkage studies and direct sequencing excluded *LMNA, MYH7, TNNT2, TNNI3, SCN5A, DES, SGCD, ACTC, PLN*, and *MYH6* but found a linkage between the TTN locus at chromosome 2q31 and DCM. Sequence analysis identified an insertion (c.58880insA), which finally caused protein truncation after 19,628 amino acids (p.S19628IfsX1). Hinson et al.^211^ indicated that titin mutations disrupted critical linkages between sarcomerogenesis and adaptive remodeling and caused DCM in iPSCs. *TTN*tv results in reduced phosphorylation levels of Troponin I (TnI) and MYBP‐C in the LV, contributing to the manifestation of frequent arrhythmias.^212^, ^213^

#### 6.1.3 GATA binding protein 4

In cardiac development, the cardiac transcription factor GATA binding protein 4 (GATA4) is essential, and mutations in *GATA4* have been associated with a wide variety of congenital heart diseases and DCM.^214^, ^215^ In recent studies, various new heterozygous *GATA4* mutations—namely, p.V291L, p.V39L, p.P226Q, and p.T279S—have been detected in three unrelated patients with sporadic DCM and in a family exhibiting DCM inheritance via an autosomal dominant pattern.^216^, ^217^

#### 6.1.4 Beta‐myosin heavy chain

The majority of pathogenic mutations found in DCM affect genes responsible for both sarcomeric and cytoskeletal proteins. Among these, mutations in the *MYH7* gene are the most prevalent. Patients with *MYH7* variants exhibit a specific correlation with LV noncompaction characteristics.^218^ Rani et al.^219^ found a novel mutation in the β‐*MYH7* gene in Indian patients with DCM.

#### 6.1.5 Lamins

*Lamins (LMNA) PP‐*associated cardiomyopathy is a form of DCM with poor prognosis and high mortality. *LMNA* DCM has gender difference, which is more severe in males in both human patients and a knock‐in mouse model carrying a homozygous p.H222P mutation (LmnaH222P/H222P).^220^ Cai et al.^221^ found that the *LMNA*‐R225X nonsense mutation induces cardiac conduction defects through AV node fibrosis, resulting in DCM.

#### 6.1.6 Junctophilin 2

Recent studies have identified two novel variants by ultra‐sequencing in patients with DCM: the p.Asn1474Lys variant in the *SCN5A* gene and the p.Glu85Lys variant in the *JPH2* gene.^222^ However, pathogenic *JPH2* variants are rare among patients with DCM.^223^

#### 6.1.7 Sodium voltage‐gated channel alpha subunit 5

Mutations in the Sodium voltage‐gated channel alpha subunit 5 (*SCN5A)* gene have been associated with the development of DCM, a total of 12 family members (10 males, 83.3%), 2 of them carriers of the p.Asn1474Lys variant in the *SCN5A* gene.^222^ Mann et al.^224^ indicated that the R222Q *SCN5A* variant activates sodium channel function and is associated with reversible ventricular ectopy and DCM.

#### 6.1.8 BAG Cochaperone 3

BAG Cochaperone 3 (*BAG3*) has been identified as one of the most common DCM causative genes in recent human genetic studies, with its variants contributing to 2.3–6.7% of DCMs.^225^ *BAG3*‐related DCM is characterized by a high penetrance in patients >40 years of age and a high risk of progressive heart failure.^226^ The mechanism of *BAG3* mutations caused DCM possibly to interfere with Z‐disc assembly and enhance sensitivity to apoptosis in cardiomyocytes.^227^ Hakui et al.^228^ identified that loss‐of‐function mutations in *BAG5* caused inherited DCM in five patients among four unrelated families with complete penetrance.

### 6.2 Therapeutic interventions

To date, there is no evidence‐based treatment for DCM, and DCM is treated the same way as heart failure.^229^ In many cases, heart transplantation remains the only option when it is impossible to inhibit the progression of heart failure through drug therapy.^230^ On this issue, gene therapy may be a promising treatment option or supplement for patients with DCM caused by genetic mutations.

#### 6.2.1 Gene therapy

More than 50 causal genes associated with DCM have been identified. The use of contemporary genetic testing has also demonstrated the underlying pathogenesis in between 50 and 75% of patients with multiple causes found in 25%.^231^ Recent studies have suggested that therapeutic strategies to attenuate disease‐causing gene activity may rescue depressed cardiac contractility in patients with DCM.^102^

##### Troponin T2

The DCM caused by *TNNT2* mutations tends to have severe clinical phenotypes and develop markedly enlarged hearts with LV systolic dysfunction and frequent SCD.^232^ Li et al.^233^ found that the expression of cardiac XIN protein was decreased in *TNNT2*‐1 K210 hESCs‐derived cardiomyocytes and the heart‐specific delivered overexpression of XINB via AAV9 could ameliorate DCM remodeling in *Tnnt2‐1* K210 mice. Migliore et al.^234^ identified that allele‐specific silencing by RNAi (ASP‐RNAi) could specifically knock down mutant alleles coding for R92Q and R173W mutant TNNT2 proteins in HCM and DCM.

The point mutation in the LMNA gene can lead to Hutchinson‐Gilford progeria syndrome (HGPS), which might cause heart complications. Lee et al.^235^ found that the antisense oligonucleotide therapy (siRNAs) targeting exon 11 could rescue LMNA‐relate progeria and reduce prelamin A/progerin in favor of the alternative splicing of lamin C. Genome editing was used to correct LMNA‐relate progeria in two similar recent preclinical study,^236^, ^237^ suggesting that in other malignant LMNA missense variants, the HGPS gene editing model could be recapitulated. PTC124 induces translational read‐through across the premature stop codon and restores the production of the full‐length proteins encoded from the mutated genes. Lee et al.^238^ found that the production of full‐length LMNA proteins was increased by PTC124 treatment and improved the excitation–contraction coupling of the affected cardiomyocytes in the R225X mutant.

##### Titin

TTN is the largest protein in humans and is required for sarcomere assembly.^239^, ^240^ It provides most of the motility and regulates the active contractile force in the striated muscle.^241^ *TTN* mutations and consequent truncated protein abnormalities are among the most common genetic causes of DCM in approximately 25% of idiopathic DCM family cases and 18% of sporadic cases.^242^ Gramlich et al.^68^ found that AON treatment in *Ttn* knock‐in mice improved sarcomere formation and contractile properties in homozygous embryos and prevented the development of DCM phenotypes in heterozygous animals. The intervention of calcium sensitivity may promote myofilament function in DCM patients with sarcomere gene mutations, thereby preventing cardiomyocyte dysfunction.^243^ Romano et al.^244^ indicated that using CRISPR to ablate A‐band variant‐specific truncation peptides by introducing a proximal I‐band *TTN*tv restored functional deficits and could be adapted as a potential genome editing strategy to target above 30% of DCM‐associated *TTN*tvs.

##### Phospholamban

Therapeutic genome editing could be effectively applied to the *PLN*, encoding the protein functions to regulate the kinetics of calcium flux in cardiomyocytes.^195^, ^245^, ^246^ Recent studies found that irregular Ca^2+^ handling, abnormal cytoplasmic PLN protein distribution, and increased cardiac hypertrophy marker expression could be exhibited in the iCMs carrying a deleterious *PLN* R14del mutation.^195^ A TALEN vector pair introducing a double‐strand break adjacent to the mutation and the gene correction matrix incorporating the wild‐type copy of the gene into the DNA via recombination could be used to correct the R14del mutation. TALEN‐mediated genetic correction restored contractile function in this model that has been impaired by *PLN* R14del mutation.^246^ Feyen et al.^194^ modeled the *PLN* R14del cardiomyopathy with isogenic pairs of hiPSC‐CMs, and single‐cell RNA sequencing revealed that the unfolded protein response pathway (UPR) had been induced in *PLN* R14del. Therefore, modulation of the UPR might be exploited therapeutically.^194^ Hoshijima et al.^247^ trans‐coronary delivered S16EPLN gene via rAAV vector, myocardial SR Ca^2+^ uptake and LV systolic function could be improved. Beverborg et al.^248^ used ASOs to target *Pln* mRNA and interfered with the *PLN/SERCA2a* interaction in the heart of murine HF models. The progression of LV dilatation had been suppressed by this therapeutic modality.^248^

##### Immunoglobulin Mu DNA binding protein 2

The therapeutic strategy of a tissue‐specific requirement for immunoglobulin Mu DNA binding protein 2 (*IGHMBP2*) has been verified effectively in cardiomyocyte maintenance and survival, and a genetic modifier has been found that it can alter the course of DCM through cardiac functional adaptation and physical remodeling.^249^ Maddatu et al.^250^ indicated that transgenic expression of the *Ighmbp2* cDNA prevented the process of impairing the function of skeletal and cardiac myocytes in mouse.

##### Apoptosis signal‐regulating kinase 1

Hikoso et al.^251^ indicated that the rAAV expressing an N‐terminal truncated form of the dominant‐negative mutant of apoptosis signal‐regulating kinase 1 (*ASK1*) inhibited ASK1 protein activation in the hamster hearts and suppressed the progression of ventricular remodeling such as chamber dilation, impairment of contractile and relaxation functions, and fibrosis.

##### Vascular endothelial growth factor B

Vascular endothelial growth factor B (*VEGF‐B*) gene transfer has shown beneficial effects in experimental models of cardiac injury.^252^, ^253^ VEGF‐B is one of the five members of the mammalian VEGFs family and is a major presurvival factor.^254^ Its remarkable cytoprotective/antiapoptotic and minimal angiogenic effects make it particularly suitable for gene therapy for nonischemic DCM.^255^, ^256^, ^257^ Woitek et al.^258^ used a dog DCM model to inject the adeno‐associated‐9 virus carrying the *VEGF‐B_167_* gene into the coronary arteries of dogs with compensated heart failure. Compared with the control group, the *VEGF‐B_167_* group revealed significant retention of diastolic and systolic function and reduced ventricular remodeling that prevented progression from compensated to decompensated heart failure.

##### MicroRNAs

MicroRNA can be highly expressed in cardiomyocytes and plays a vital role in muscle growth, regeneration, and fibrosis processes. Misexpression of miRNA could have severe effects on cardiomyocytes. Studies have shown that down‐regulation of miR‐448‐3p can trigger reactive oxygen species (ROS) production and lead to cardiac hypertrophy, atrial fibrillation, myocardial fibrosis, and inflammation, resulting in DCM.^259^

Studies have shown that approximately 8% of sporadic and 25% of familial DCM are associated with truncated mutations in the gene encoding casin. Moreover, the frameshift mutation of the annexin‐encoding gene mediated by antisense oligonucleotides is one of the critical genetic forms of DCM. Gramlich et al.^68^ exhibited that the beneficial potential of AON‐mediated exon skipping could be used to reframe titin transcripts in humans. The reframed TTN could produce three major isoforms (N2A, N2B, and N2BA) by alternative splicing, which predominately differ in the length of the extensible I‐band domains.^260^ Quattrocelli et al.^261^ demonstrated that intraventricular delivery of AAV vectors induces long‐term (18 months) miR‐669a overexpression significantly improved myocardial structure and cardiac function in Sgcb gene knockout mice, and it reduced adverse LV remodeling, thereby attenuating malnutrition and improving survival in mice with severe cardiomyopathy rate. In addition, drug therapy can also regulate the expression of mRNA in cardiomyocytes. Sukumaran et al.^261^ found that olmesartan treatment upregulated myocardial protein and mRNA levels of ACE‐2, ANG 1−7 receptor but effectively suppressed the myocardial protein and mRNA expressions of inflammatory markers compared with the vehicle‐treated DCM rats.

#### 6.2.2 Therapeutic dilemma

The degree of genetic mutation varies from gene to gene. For example, the *LAMIN A/C* mutation is highly porous and therefore requires correction by silencing the mutated gene.^262^ siRNA‐mediated deleterious allelic silencing can be used to treat more malignant genetic mutations. Conversely, some benign gene mutations (such as *TTN* truncation mutations) with cardiac function that are likely to improve after treatment do not benefit from gene silencing therapy.^263^ The phenotypic manifestation of a genetic variation will also determine the treatment. Antiarrhythmic therapy is more often considered in DCM patients with *LMNA* or *RBM20* gene mutations, whereas standard heart failure therapy may be sufficient in other benign DCM patients.^264^, ^265^, ^266^, ^267^

## 7 RESTRICTIVE CARDIOMYOPATHY

RCM is a rare form of cardiomyopathy characterized by restrictive ventricular physiology in the presence of normal diastolic volume and normal ventricular wall thickness. Genes associated with RCM include those encoding myosin heavy chain, myosin binding protein C, troponin I and T, tropomyosin, and desmin (DES). RCM is rarer than HCM and DCM, but the prognosis is worse, and the risk of pulmonary hypertension, thromboembolic events, and sudden death is higher.^268^, ^269^ Although the primary molecular mechanism in RCM is not fully understood, recent studies have advanced hypotheses based on experimental models.

### 7.1 Pathogenesis and disease‐causing genes

Primary RCM is usually related to genetic factors, and its pathogenesis mainly involves the increased sensitivity of cardiac muscle filaments to calcium, as well as the accumulation of DES and type III collagen within the cardiac muscle cells, which are usually caused by mutations in the related encoding genes. Alterations in genes encoding for sarcomeric proteins, Z‐disc proteins, or transthyretin (TTR) have been identified as the potential disease‐causing genes of RCM.^17^ Causes of RCM with associated genetic perturbations included mutations in genes encoding sarcomeric proteins (MYH7−4, MYBPC3−3, TNNI3−2, and TNNT2−1), genes encoding structural and genes encoding cytoskeletal proteins (BAG3, JUP, ACTN2, DES). Among 24 variants of unknown significance, 19 were identified in structural and cytoskeletal genes (*TTN, SYNE, MYOM1, CACNB2, FKTN, LDB3, EMD, MYOZ, DSP, TMPO*), two in ion channel genes, and two in genes encoding mitochondrial proteins.^270^, ^271^ Gallego‐Delgado et al.^272^ analyzed the variants in genes associated with RCM in 32 unrelated patients and found that mutated genes included *MYH7* (four patients), *DES* (three), *FLNC* (three), *MYBPC3* (two), *LMNA* (two), *TCAP* (one), *TNNI3* (one), *TNNT2* (one), *TPM1* (one), and *LAMP2* (one). Eleven patients (34%) exhibited only VUS and two patients(6%) did not bear any mutation. DNA samples from 12 children have been analyzed in a recent study, which identified sarcomere protein gene mutations in four patients (33%): two in *TNNI3* and one each in *TNNT2* and *ACTC* genes.^273^ Caleshu et al.^17^ first reported that mutations in *TPM1, MYL3*, and *MYL2* were associated with primary, nonhypertrophied RCM in two DCM patients.

Recent studies have found different mutations associated with RCM in patients, in vitro, and in animals (Figure 4B).^17^, ^273^, ^274^, ^275^, ^276^, ^277^, ^278^, ^279^, ^280^, ^281^, ^282^, ^283^, ^284^, ^444^

### 7.2 Therapeutic interventions

RCM is relatively rare compared with HCM and DCM, with diverse inherited and acquired causes and manifestations. Elimination of the pathogenic protein and organ recovery is the goal of effective treatment.^271^ There is no approved therapy that targets the underlying genetic cause. Zaleta‐Rivera et al.^285^ found that a one‐time injection of AAV9–M7.8L R into 3‐day‐old humanized regulatory light chain mutant transgenic mice silenced the mutated allele (RLC‐47K). The expression of hypertrophic biomarkers was suppressed and the pathological increase was attenuated in the LV mass, which showed RNAi therapeutics may be a feasible and safe treatment strategy directed toward human RCM. This is a promising step toward targeted gene therapy for RCM.^286^

## 8 LEFT VENTRICULAR NONCOMPACTION

LVNC, characterized by abnormal trabeculations in the LV, is the third most common cardiomyopathy in children. The intrauterine arrest of compaction of the loosely woven meshwork that constitutes the fetal myocardial primordium leads to an altered myocardial wall. In at least 30−50% of patients, the development of the condition can be attributed to genetic inheritance, and researchers have identified several genes responsible for LV noncompaction.^286^

### 8.1 Pathogenesis and disease‐causing genes

LVNC, characterized by abnormal trabeculations in the LV, is the third most common cardiomyopathy in children. The intrauterine arrest of compaction of the loosely woven meshwork that constitutes the fetal myocardial primordium leads to an altered myocardial wall. In at least 30−50% of patients, the development of the condition can be attributed to genetic inheritance, and researchers have identified several genes responsible for LV noncompaction.^286^ The pathogenesis of LVNC involves mutations in multiple genes. These include genes encoding for sarcomeric (*MYBPC3*, *TPM1*, *MYH7*, *ACTC1*, *TNNT2*,^18^ *TNNI3*, *MYL2*, and *MYL3* ^287^), Z‐disc (*LDB3*, *Cypher*, *ZASP*, *DSC2* ^287^, ^288^), nuclear envelope (e.g., *LMNA*), mitochondrial (*SCO2*, *SDHA*, *TAZ*), ion channel proteins (*ABCC9*, *ANK2*, *CACNA1C*, *KCNE3*, *KCNH2*, *KCNQ1*, *RYR1*, *RYR2*, *SCN5A*, *HCN4* ^289^, ^290^), α‐dystrobrevin (*DTNA*), and NOTCH pathway regulators (e.g., *MIB1*), muscle‐specific intermediate filament protein desmin (e.g., *DES*), AMP‐activated protein kinase (*PRKAG2*). The different genotype may correlate with different phenotype.^291^, ^292^, ^293^

Kolokotronis et al.^294^ revealed that severe cardiomyopathy accompanied by LVNC could be caused by a missense variant in either *MYH7* or *MYBPC3*. Kodo et al.^295^ discovered that by inhibiting TGFβ signaling and correcting the *TBX20* mutation in iPSC‐CMs from LVNC patients, the disease phenotype associated with the cardiac transcription factor *TBX20* mutation could be reversed.

### 8.2 Therapeutic interventions

#### 8.2.1 Pharmacological treatment and surgical treatment

The drug treatment for LVNC primarily targets the symptoms of heart failure, arrhythmias, and thromboembolism that may arise from the condition. In treating heart failure, standard antiheart failure medications are typically used, and for end‐stage heart failure patients, heart transplantation should be considered. For ICDs, the indications for secondary or primary prevention are the same as for other cardiomyopathies, and appropriate ICD implantation can effectively prevent sudden death and reduce mortality rates. Additionally, since patients with LVNC may be at high risk for thromboembolism, long‐term anticoagulation therapy is recommended for patients with atrial fibrillation, a history of systemic embolism, or a LVEF less than 40%. In the case of drug treatment for patients with LVNC, there are currently no specific drug guidelines and treatment mainly involves symptomatic therapy and management of complications.^296^

#### 8.2.2 Gene therapy

Although numerous mutations have been discovered in the genes associated with LVNC, the exact mechanism behind ventricular compaction and the pathogenesis of LVNC remains to be fully elucidated. With a large number of genes leading to LVNC, genetic testing plays a minor role in clinical practice at this time.^297^ There is no targeted gene therapy at present, but we believe that the molecular underpinnings and genetic mechanisms behind LVNC will soon be discovered, and then LVNC patients will be able to receive the proper treatment targeted to their specific pathogenic gene mutation.

## 9 GLYCOGEN STORAGE CARDIOMYOPATHY

Glycogen storage cardiomyopathy is a group of genetic disorders characterized by the abnormal accumulation of glycogen in cardiac muscle tissue, leading to impaired heart function. This disease can result in glycogen accumulation in various tissues, especially in skeletal and cardiac muscle tissue, causing a genetics storage cardiomyopathy with enlarged glycogen vacuoles.^298^, ^299^ Glycogen storage cardiomyopathy usually contains Pompe disease (PD), PRKAG2 syndrome, and LAMP2 syndrome.

### 9.1 PRKAG2 cardiomyopathy: pathogenesis and therapeutic interventions

HCM is characterized by mutations in the contractile elements of the sarcomere or Z‐disc. Moreover, certain cardiomyocytes exhibiting unexplained hypertrophy may harbor mutations in additional genes. One such gene is *PRKAG2*, responsible for encoding the gamma2 subunit of AMPK, causing an accumulation of cardiac glycogen and LV hypertrophy.^298^, ^300^, ^301^, ^302^ Laforêt et al.^303^ reported 38‐year‐old male presenting with HCM, which was attributed to a recently discovered heterozygous *PRKAG2* mutation (Ser548Pro). Recent research has provided evidence of an elevated susceptibility to arrhythmic and myocardial complications in individuals affected by cardiac glycogenosis caused by *PRKAG2* mutations.^304^, ^305^ CRISPR technology could correct the mutation and ameliorate disease in the patient's iPSCs.^176^ Xie et al.^306^ injected AAV9–Cas9/sgRNA either on postnatal day 4 or day 42, resulting in significant restoration of the cardiac morphology and function in H530R *Prkag2* transgenic and KI mice.

### 9.2 LAMP2 cardiomyopathy: pathogenesis and therapeutic interventions

Human mutations in the X‐linked *LAMP2* gene can cause Danon disease (DD), a lysosomal glycogen storage disease with fatal cardiomyopathy.^307^, ^308^ In *LAMP‐2* deficient myocytes, cardiac contractile function is significantly attenuated.^309^ Dvornikov et al.^310^ discovered that partial mTOR deficiency had the ability to restore certain features of *lamp2* KO in zebrafish, such as ejection fraction and actomyosin activation kinetics. These findings hold promise for the development of targeted gene therapies aimed at addressing these specific characteristics.^310^ In an open‐label Phase 1 clinical trial, a single IV administration of RP‐A501 (AAV9.LAMP2B) was shown to effectively deliver and transduce cardiomyocytes in patients afflicted with DD—an X‐linked monogenic cardiomyopathy attributed to mutations in the LAMP2 gene. This targeted therapy not only arrested but also demonstrated the potential to reverse the disease's rapid progression of cardiomyopathy.^311^

### 9.3 Pompe disease: pathogenesis and therapeutic interventions

PD, also known as glycogen storage disease type II, is an autosomal recessive metabolic disorder that ultimately causes damage to muscle and nerve cells throughout the body. The condition arises due to a deficiency in the lysosomal enzyme acid α‐glucosidase (GAA), leading to the accumulation of glycogen within the lysosome.^312^ Recently, a large number of studies have demonstrated the feasibility of AAV vectors for in vivo *GAA* gene therapy are available. Pauly et al.^313^ constructed an E1‐deleted recombinant adenovirus encoding human *GAA*. They demonstrated that recombinant acid GAA could express at a high level in treated target tissues, which supported the availability of gene replacement strategies for PD.^313^ Mah et al.^314^ showed that the deficiency of cardiac and diaphragmatic *Gaa* enzymatic activity in mice with PD could be successfully restored through the use of a rAAV serotype 1 (rAAV2/1) vector administered in vivo. Keeler et al.^315^ found that both AAVB1 and AAV9 vectors expressing *GAA* transduced the heart efficiently, leading to glycogen clearance. Stok et al.^316^ reported the normalization of glycogen in heart tissue by ex vivo hematopoietic stem cell gene therapy. They have used a codon‐optimized *GAA* (*GAA*co) to normalize the glycogen in the heart, muscles, and brain in enzyme levels.^316^ Liang et al.^317^ utilized a fusion approach by combining insulin‐like growth factor 2 (IGF2) with a codon‐optimized variant of *GAA* (LV‐IGF2.*GAA*co) to enhance cellular uptake. This fusion construct demonstrated the ability to fully restore glycogen levels, alleviate pathology, and improve impaired autophagy in both heart and skeletal muscles. Importantly, these effects were achieved at a clinically relevant vector copy number of 3.^317^

## 10 BARTH SYNDROME

### 10.1 Pathogenesis and disease‐causing genes

Barth syndrome (BTHS) arises from mutations in the gene responsible for encoding tafazzin (TAZ), leading to a mitochondrial disorder. The mutations resulted in muscle dysfunction and DCM, characterized by abnormal sarcomere assembly, compromised contractility, and an excessive presence of ROS.^318^ In a cellular experiment, Wang et al.^319^ utilized iPSCs derived from a healthy individual and introduced *TAZ* mutations. They successfully demonstrated that *TAZ* mutation alone is both necessary and sufficient to induce the cardiac myocyte dysfunction associated with BTHS. These findings suggest potential new treatment approaches, such as in vivo gene therapy targeting the *TAZ* gene, to rescue cardiomyopathy in patients with BTHS.^319^

### 10.2 Potential therapeutic interventions

In recent studies, AAV has been used for gene therapy to deliver *Taz* since AAV is long‐lasting and provides stable gene transfer.^320^ Wang et al.^47^ showed that the replacement of Taz through AAV gene therapy effectively rescued neonatal death, cardiac dysfunction, and fibrosis in *Taz*‐KO mice. Suzuki‐Hatano et al.^321^ indicated that AAV‐mediated *TAZ* gene replacement restored mitochondrial and cardioskeletal function by improving the Des promoter. Moreover, the therapeutic targeting of *YAP* signaling has emerged as a promising approach for the treatment of pressure overload‐induced heart disease, suggesting potential efforts to treat BTHS by targeting *YAP*.^322^

## 11 CONDUCTION AND ION CHANNEL DISORDERS

### 11.1 Lamin A/C gene (LMNA) cardiomyopathy

#### 11.1.1 Pathogenesis and disease‐causing genes

*LMNA* cardiomyopathy is caused by mutations in the *LMNA*, encoding the nuclear proteins lamin A/C. The main clinical manifestations are cardiomyopathy and conduction disorders. In an iPSCs model of the K219T mutation on *LMNA*, Salvarani et al.^323^ revealed that the K219T‐LMNA mutation synergistically interacted with *PRC2*, leading to the downregulation of *SCN5A*, resulting in a reduction in sodium current density and a slowdown in conduction velocity. This mechanism potentially contributes to the conduction abnormalities observed in *LMNA* cardiomyopathy.^323^ Gerbino et al.^324^ investigated a newly discovered frameshift variant on the *LMNA* gene (p.D243Gfs*4), which was found in three individuals from an Italian family and was found to be cosegregating with a severe manifestation of cardiac conduction defects. The results indicated that the aberrant CX43 expression participated in the pathogenic mechanism for this *LMNA* truncating alteration.^324^ Moreover, Cai et al.^221^ indicated that the *LMNA*‐R225X nonsense mutation induced cardiac conduction defects and DCM.

#### 11.1.2 Potential therapeutic interventions

In terms of treatment, there is currently no specific therapeutic method for *LMNA*‐related cardiomyopathy. However, research is exploring the possibility of interventional treatment by targeting epigenetic modifiers or specific signaling pathways. Sun et al.^325^ identified that whole‐body supplementation with *LMNA* using rAAVs partially ameliorated the cardiac abnormalities in mouse models harboring *Lmna* truncating variants. This finding implicates AAV‐mediated gene supplementation as an emerging and promising therapeutic approach for the treatment of *LMNA*‐related cardiomyopathy.^325^ Lee and his team^326^ established an in vitro model for *LMNA*‐DCM by utilizing patient‐specific iPSC‐CMs. Through this model, they discovered that the activation of the PDGF pathway plays a significant role in the pathogenesis of *LMNA*‐DCM. They also proposed that PDGF receptor beta (PDGFRB) might represent a promising therapeutic target for this condition.^326^ Chen et al. identified the E2F/DNA damage response/TP53 axis as a mechanism underlying the development of DCM in laminopathies and suggested it as a potential target for interventions.^327^ Upregulated MAP kinase signaling pathway in the heart was detected in the lamin A/C gene mutated mouse. Loss of functional lamin protein leads to activation of the p38 MAPK pathway, secondary to cardiomyocyte apoptosis and interstitial fibrosis.^328^ Therefore, the MAP kinase signaling pathway may potentially serve as a therapeutic target for *LMNA*‐related cardiomyopathy. Choi et al.^329^ found that in vivo administration of the rapamycin analog temsirolimus prevented the hyperactivation of the AKT–mTOR pathway in the hearts of mice with cardiomyopathy caused by the *LMNA* mutation and suppressed the deterioration of cardiac function. Chatzifrangkeskou et al.^330^ showed that TGF‐β/Smad signaling participated in activated ERK1/2 signaling in *LMNA* cardiomyopathy, leading to altered activation of CTGF/CCN2 to mediate fibrosis and altered left cardiac function, which indicated a novel therapeutic target in the treatment of LMNA cardiomyopathy. Wu et al.^331^ also demonstrated that inhibitors of ERK and JNK signaling could potentially be used to treat humans with DCM caused by *LMNA* insufficiency. Tan et al.^332^ found that upregulation of Yy1 suppressed Lmna DCM and cardiac fibrosis by inducing Bmp7 expression and preventing upregulation of Ctgf, which offered novel therapeutic strategies for the treatment of *LMNA*‐related DCM.

### 11.2 Brugada syndrome

#### 11.2.1 Pathogenesis and disease‐causing genes

Brugada syndrome (BrS) is an inherited disease associated with loss‐of‐function mutations in the cardiac sodium channel Nav1.5, which is encoded by the *SCN5A* gene.^333^ Genetic tests for diagnosis of BrS should include screening for the pathogenic variant in one of 23 genes: *ABCC9*, *CACNA1C*, *CACNA2D1*, *CACNB2*, *FGF12*, *GPD1L*, *HCN4*, *KCND2*, *KCND3*, *KCNE5*, *KCNE3, KCNH2*, *KCNJ8*, *PKP2*, *RANGRF*, *SCN1B*, *SCN2B*, *SCN3B*, *SCN5A*, *SCN10A*, *SEMA3A*, *SLMAP*, and *TRPM4*.^334^, ^335^ Currently, *SCN5A* and *SCN10A* have attracted the most attention as significant susceptibility genes for BrS.^336^, ^337^ Furthermore, the mutations in different genes are related to phenotype severity. Individuals with mutations in the *SCN5A* gene show heightened epicardial electrical abnormalities and a more severe clinical presentation.^338^

#### 11.2.2 Potential therapeutic interventions

In recent years, ICDs have been proven effective for the treatment of BrS, while epicardial ablation of the right ventricular outflow tract has shown success in reducing the incidence of arrhythmias and normalizing the ECG patterns in BrS patients.^339^

Disease models and experimental therapeutic techniques have developed rapidly in recent years. Cellular reprogramming and gene editing through the technology of IPSCs has been described for different primary cardiomyopathies with cardiac conduction dysfunction.^340^ Liang et al.^341^ used iPSC‐CMs to explore the pathomechanism of BrS. They then corrected the proposed causative SCN5A variant and rescued the phenotypes, including abnormal Ca^2+^ transients, via CRISPR/Cas9 genome editing.^341^ Teng et al.^342^ suppressed these *SCN5A* nonsense mutations by utilizing two readthrough‐enhancing methods (either aminoglycosides or a siRNA‐targeting eukaryotic release factor eRF3a (a GTPase that binds eRF1)), which suggested nonsense mutations in *SCN5A* could be suppressed. The expression of full‐length channels could be restored effectively via readthrough‐enhancing methods. Yu et al.^343^ employed AAV9 vector‐based delivery of *MOG1* to enhance *MOG1* expression. This increase in *MOG1* led to elevated cell surface expression of NaV1.5, resulting in augmented ventricular INa levels. As a result, they were able to successfully mitigate the symptoms of cardiac arrhythmias and contractile dysfunction in heterozygous humanized knock‐in mice carrying the *SCN5A* mutation p.D1275N. Chakrabarti et al.^344^ discovered that MOG1 effectively restored diminished PM expression. Consequently, utilizing *MOG1* to enhance Nav1.5 trafficking holds promise as a targeted therapeutic approach for certain patients with BrS in future applications.

### 11.3 Long QT syndrome

#### 11.3.1 Pathogenesis and disease‐causing genes

The most common forms of long QT syndrome (LQTS) mutations with LQTS are uncovered in *KCNQ, KCNH2*, and *SCN5A*, accounting for approximately 75% of genotype‐positive LQTS cases.^345^ LQTS, BrS, and cardiomyopathy may all be caused by *SCN5A* mutations,^336^ encoding the alpha‐subunit of the Nav1.5 ion channel protein responsible for the sodium inward current (INa).^334^ Investigating *SCN5A* variations in various *SCN5A*‐related cardiac conditions and exploring newly developed therapeutic strategies could prove valuable in the clinical setting for the prevention and treatment of these disorders.^346^

#### 11.3.2 Potential therapeutic interventions

Given that LQTS arises from a gain‐of‐function in the SCN5A channel, sodium channel blockers, SGLT2 inhibitor and SGK1 inhibitors are potential candidate drugs for its treatment.^347^ Recently, research has shown that SGK1 inhibitors can selectively diminish the late sodium current (INa‐L) and abbreviate the action potential duration (APD) in various contexts. Their efficacy has been demonstrated in iPSC‐derived cardiomyocytes (iPSC‐CMs) harboring the SCN5A–N406K mutation, as well as in iPSC‐CMs treated with dofetilide.^348^, ^349^ Furthermore, an SGLT2 inhibitor has been demonstrated to decrease the INa‐L in cardiomyocytes from mice with heart failure, as well as in cells expressing the SCN5A–R1623Q or SCN5A–ΔKPQ mutations.^350^

Dotzler and colleagues developed a dual‐component gene therapy called *KCNQ1* Suppression‐and‐Replacement (SupRep) by introducing a *KCNQ1* short hairpin RNA and a short hairpin RNA‐resistant *KCNQ1* cDNA into LQT1 iPSC‐CMs. The *KCNQ1* SupRep gene therapy effectively reduced APD, thereby eliminating the characteristic feature of LQT1, which is a significant step in the treatment of this condition.^351^ They provided a promising therapeutic approach for LQTS.

### 11.4 Short QT syndrome

#### 11.4.1 Pathogenesis and disease‐causing genes

Short QT syndrome (SQTS) is a rare, life‐threatening, inherited heart disease presenting a family history of SCD in 15%. Therefore, genetic testing is important in the diagnosis of SQTS, but the causative mutation has been found in <25% of cases up to now.^352^ The mutations associated with SQTS usually happened in genes including *KCNH2*, *KCNQ1*, *KCNJ1*, *CACNA1C*, *CACNB2*, and *CACNA2D1*, responsible for SQT‐1–8 subtypes, respectively.^353^ In children carrying a *KCNH2*–V141M mutation, a nonthreatening form of the disease has been noted. Additionally, recent studies suggest that SQTS may arise from a mutation in the cardiac Cl/HCO3 exchanger AE3.^352^

#### 11.4.2 Potential therapeutic interventions

At present, the primary treatment approach for individuals with SQTS involves the use of an ICD, while quinidine remains the sole medication to have undergone clinical evaluation for this condition.^353^ Targeted gene therapy is not currently available. Some studies have found that *KCNH2* mutation may be the potential target for therapy in SQTS patients.^354^ To uncover fresh potential targets, an exome or genome sequencing methodology is essential.^355^

### 11.5 Sudden unexpected nocturnal death syndrome

Sudden Unexpected Nocturnal Death Syndrome (SUNDS) is a phenomenon that predominantly affects young, seemingly healthy Southeast Asian individuals, with a higher prevalence among men. The exact pathophysiological mechanisms of SUNDS remain elusive; however, several factors and genetic predispositions have been proposed to contribute to its development.^356^ Roughly 50% of the ion channel anomalies linked to SUNDS are attributed to variations in the *SCN5A* gene or abnormalities in other constituents of the sodium channel macromolecular complex.^357^ Variants in this gene can lead to sodium channel dysfunction, potentially resulting in disturbed cardiac conduction and an increased risk for life‐threatening arrhythmias, such as ventricular fibrillation, especially during sleep.

While the precise mechanisms of SUNDS are not fully understood, it is believed to be a multifactorial condition involving genetic predispositions, possibly interacting with environmental and lifestyle factors to increase the risk of SCD during sleep. Further research is needed to elucidate the pathophysiology of SUNDS and to develop effective preventive strategies.

## 12 CARDIAC AMYLOIDOSIS

Cardiac amyloidosis (CA) is a progressive cardiomyopathy caused by an accumulation of endogenous proteins that fold and degrade in the heart (mainly in the kidneys, liver, gastrointestinal tract, and soft tissues with amyloid fibrils). Clinically, systemic amyloidosis is divided into five types, primary amyloidosis (AL) (or light chain amyloidosis), secondary (AA) amyloidosis (or reactive amyloidosis), familial amyloidosis (ATTR or hereditary amyloidosis), dialysis‐related amyloidosis and senile systemic amyloidosis (SSA). Of these, AL, ATTR, and SSA are commonly involved with the myocardium.^358^ ATTR has become the most frequent type of CA in clinical practice.^359^ However, the prognosis of amyloidosis with cardiac involvement is poor. The average survival time in AL‐CA patients is 6 months, and in ATTR‐CA patients is 26−43 months.^360^ Cardiac involvement in ATTR typically manifests in the sixth and seventh decades of life, presenting as heart failure with preserved ejection fraction (HFpEF), with “wild‐type” or “senile systemic amyloidosis” being the predominant cause in the United States.^361^, ^362^

### 12.1 Primary amyloidosis

#### 12.1.1 Pathogenesis

Plasma cells in the bone marrow (a type of B lymphocyte) abnormally proliferate to form clonal plasma cells. These abnormal plasma cells produce abnormal monoclonal immunoglobulin light chains, which under normal conditions should bind with other parts of the immunoglobulin, but may exist in a free form in pathological states. Due to their structural characteristics, these monoclonal light chains are prone to misfolding and form beta‐sheet structures, which then spontaneously aggregate to form oligomers and fibrils. They further aggregate to form insoluble amyloid protein deposits, which accumulate in cardiac tissue, particularly in the subendocardial and myocardial interstitial regions. The deposition of amyloid proteins leads to an increase in the extracellular matrix of cardiomyocytes, affecting the normal contraction and relaxation functions of the heart, causing an increase in cardiac stiffness, and ultimately may progress to heart failure.^363^

#### 12.1.2 Potential therapeutic interventions

Previous treatments for AL amyloidosis have often been based on bortezomib‐based regimens for multiple myeloma, but there has still been a significant unmet clinical need, and researchers have never stopped exploring new protocols. In recent years, with the emergence of immunotherapies such as monoclonal antibodies, the survival rate of patients with AL amyloidosis has improved compared with the past. Dara (daratumumab) is a monoclonal antibody targeting the CD38 antigen on the surface of plasma cells and has shown good efficacy in the treatment of AL‐CA, especially when used in combination with the CyBorD regimen.^364^ As research progresses, some new drugs, such as Belantamab mafodotin, are being evaluated in clinical trials for their efficacy and safety in patients with AL‐CA.^130^, ^131^, ^132^

### 12.2 Familial amyloidosis

#### 12.2.1 Pathogenesis and disease‐causing genes

The pathogenesis of ATTR mainly involves the deposition of misfolded TTR in cardiac muscle tissue. Under normal conditions, TTR is a soluble tetramer responsible for transporting thyroxine and retinol, but when TTR dissociates into monomers and misfolds, it forms amyloid substances that deposit in the myocardial interstitium, ultimately leading to myocardial disease and progressing to progressive heart failure.

Researchers believe that approximately 10% of amyloidosis gene variants are present in suspected patients with systemic AL amyloidosis.^365^ The *TTR* gene, located on chromosome, 18 has more than 130 known pathogenic variants that result in different phenotypic manifestations. Genetic variation is largely inherited by autosomal dominant inheritance, and its penetrance often changes.

The Val30Met mutation is the most common in the *TTR* gene worldwide.^366^ This mutation showed a variety of phenotypic expressions, and almost all forms have neurological symptoms, among which lower extremity neuropathy (familial amyloid polyneuropathy) is more common than others. Usually, the disease has a bimodal manifestation, early‐onset (30–40 years of life) rarely with cardiomyopathy, and late‐onset (50–60 years of life) with heart involvement.^367^ The Val122Ile mutation is the most prevalent genetic variant in the United States, which mainly occurs in patients of African descendant and causes almost only heart disease.^362^ The Cardiovascular Health Study reported that after 65 years of age, Val122Ile patients had a higher incidence of heart failure symptoms (38 vs. 15%) and mortality (76 vs. 53%) compared with the general population. However, there was no difference between the two groups was found in patients <65 years of age.^368^ In Europe, Val122Ile is also considered to be a common cause of heart failure.^369^ The most common variant leading to cardiomyopathy in the UK is Thr60Ala.^370^ In the United States, this mutation is found in 20% of patients with THAOS.^362^ In an epidemiological analysis of British patients with the Thr60Ala mutation, cardiac involvement was found in 93% of patients by echocardiography. Most patients also show autonomic or peripheral neuropathy. In Japan, the first case of sporadic hereditary V122I ATTR amyloidosis was reported in male patients.^371^

Apart from these common mutations associated with ATTR amyloidosis, there are some rare mutations in the *TTR* gene have been found. Nakase et al.^372^ reported that a novel mutation (Y114S, p.Y134S) in the *TTR* gene was found in a 65‐year‐old Japanese man who suffered from hereditary amyloidosis, which was characterized by progressive cardiomyopathy with a poor vital prognosis. Bauer et al.^373^ found a predominantly cardiac phenotype with high penetrance and late onset of symptoms in ATTR amyloidosis caused by the *TTR* Val20Ile mutation and progressed to end‐stage heart failure within a few years. Moreover, p.Ser43Asn is a sporadic *TTR* mutation leading to familial ATTR amyloidosis, associated almost invariably with an isolated cardiac phenotype.^374^

#### 12.2.2 Potential therapeutic interventions

Genetic variation in amyloid heart disease provides an opportunity to explore potential treatments for the disease, especially for ATTR. Based on the elucidation of the mechanisms of amyloid formation, targeted gene therapies are now approved for treating ATTR‐CA.^375^ Genetic treatment for ATTR involves inhibiting the production of abnormal TTR proteins by the liver, stabilizing the TTR tetramer, and breaking down amyloid fibrils by interfering with the protein. Gene‐based therapies work by preventing the liver from producing TTR. These newly developed therapies can be achieved by using small interfering ribonucleic acid (siRNA), ASOs, or CRISPR–Cas9 system.^376^

##### RNA interference

The purpose of siRNA is to knock down the production of hepatic mutants and wild‐type ATTR, thereby reducing unstable cyclic TTR tetramers and preventing organ deposition of TTR monomers and amyloid fibrils, eventually resolving the disease. The feasibility of this method was confirmed using siRNA in a mouse model of hereditary ATTR. Moreover, the extent of TTR tissue sediment degradation appears to be linearly related to RNAi‐mediated knockdown and serum TTR protein exposure.^377^

##### Antisense oligonucleotides

ASO can hybridize with native mRNA to form double‐stranded RNA, which is recognized and cleaved by RNase. Inotersen is a 2′‐O‐methoxyethyl‐modified ASO that selectively binds to mRNA encoding TTR and causes mutant and wild‐type *TTR* mRNA degradation, preventing synthesis of TTR proteins (mainly in the liver). *TTR* ASOs suppressed hepatic *TTR* mRNA levels and serum TTR levels by as much as 80%, leading to a significant decrease in circulating and wild‐type TTR protein levels, thereby reducing amyloid deposition.^378^, ^379^ The Phase III NEURO‐TTR trial showed that inotersen improved neurological disease and quality of life in patients with ATTR.^137^ Recent studies have shown that ASO treatment of patients with moderate to advanced ATTR cardiomyopathy could prevent the disease progression, and therefore the life expectancy may be enhanced.^380^ Currently, regulatory reviews of inotersen are conducted in the US and Canada.

##### CRISPR–Cas9 system

Gillmore and colleagues proposed NTLA‐2001 as an in vivo gene‐editing therapeutic utilizing the CRISPR–Cas9 system. This agent comprises a lipid nanoparticle encapsulating messenger RNA for the Cas9 protein and a single guide RNA targeting *TTR*. The administration of NTLA‐2001 resulted in a reduction of serum TTR protein concentrations achieved by specifically knocking out the *TTR* gene.^381^

## 13 MUSCULAR DYSTROPHIC

### 13.1 Pathogenesis and disease‐causing genes

Muscular dystrophies encompass a range of genetic conditions characterized by gradual muscle weakening and wasting. Duchenne muscular dystrophy (DMD) and Becker muscular dystrophy (BMD) are closely associated disorders primarily impacting skeletal and cardiac muscles, leading to progressive muscle weakness, cardiomyopathy, and a reduced lifespan.

In DMD, the absence or dysfunction of dystrophin leads to muscle fiber damage, necrosis, and a reduced capacity for regeneration, which manifests clinically as progressive muscle weakness and eventual replacement of muscle tissue with connective and fatty tissue, leading to pseudohypertrophy, particularly in the calves. The disease typically presents in early childhood, with symptoms such as difficulty in running, jumping, climbing stairs, and rising from the floor, as well as the development of a characteristic waddling gait and Gower's sign. BMD is a milder form of the disease, also caused by mutations in the dystrophin gene, but with later onset and slower progression.^382^

Cardiomyopathy, which emerges in adulthood, affects nearly all patients with DMD and stands as the primary cause of death in this condition. Both DMD and BMD are caused by mutations in the X‐linked *dystrophin* (*DMD*) gene. Wong et al.^383^ showed that deletion mutation of exons 52−54 led to the absence of dystrophin, presenting with early‐onset HCM in mice models. In recent years, there have been significant advances in the understanding of the pathogenesis of DMD, leading to the development of targeted therapies such as exon‐skipping drugs, gene replacement using AAVs, and CRISPR gene editing technologies, which are currently at the forefront of research and emerging as promising treatment options for DMD.

### 13.2 Potential therapeutic interventions

#### 13.2.1 Duchenne muscular dystrophy

AAV‐mediated micro‐dystrophin gene therapy has significantly prevented the progress of the disease in rodent models associated with DMD. Validation of these encouraging results in large animal models will outline the path forward to human trials.^384^, ^385^ Mice are engineered to replicate a frequently observed set of mutations found in patients with DMD, and this dystrophin‐deficient mouse DMD model is named mdx mice. Recent studies have shown that intravascular delivery of AAV micro‐dystrophin could significantly ameliorate muscle pathology, attenuate dystrophic cardiomyopathy, and normalize the heart rate, PR interval, and QT interval in animals.^386^, ^387^ These discoveries hold significant implications for utilizing AAV gene therapy in managing DCM and heart failure.^388^ Moreover, AAV‐mediated cardiac transduction with other proteins also could attenuate dystrophic cardiomyopathy. Bauer et al.^389^ indicated that AAV9–βARKct—cDNA with a cardiac‐specific promoter injection into mdx mice over a long time could obviously improve LV systolic function and ameliorate myocardial hypertrophy. Xu and colleagues discovered that administering rAAVrh74.MCK.GALGT2 to mdx hearts prevented initial LV remodeling and the expression of fibrotic gene markers.^390^ Several clinical trials are now underway to advance therapy to DMD patients.^133^, ^134^, ^135^

CRISPR‐mediated genome editing has proven effective in enhancing dystrophin expression and improving cardiac function in mdx mouse and deltaE50‐MD dog models following a sole systemic administration of recombinant AAVs.^391^, ^392^ Xu et al.^393^ suggested that in vivo CRISPR genome editing could be developed as a safe treatment for DMD and did not lead to other deleterious defects, and dystrophin restoration could be increased. Fibrosis could be reduced via systemic AAV CRISPR therapy with an increased dose of the guide RNA vector in all striated muscles at 18 months in a mouse mdx model of DMD.^394^ In mdx mice, recent studies found that a premature termination codon located in exon 23 of the *DMD* gene has been pinpointed. Three research teams employed AAV vectors to administer guided RNAs (gRNAs) and CRISPR/Cas9 into mdx muscles, targeting the mutated exon 23 of the *DMD* gene. This approach effectively removed the premature termination codon, restored the expression of truncated yet partially functional dystrophin through exon skipping, and notably extended the survival of mdx mice.^395^, ^396^, ^397^, ^398^ Immunofluorescence data suggested that the expression of dystrophin protein was restored to a level approaching 40% via CRISPR/Cas9 in dystrophic cardiac muscles.^391^ Zhang et al.^399^ deployed a unique class 2 CRISPR effector Cpf1, the *DMD* mutations could be successfully corrected through Cpf1, and pathophysiological hallmarks of muscular dystrophy in patient‐derived iPSCs and mdx mice also could be improved.

Goyenvalle et al.^400^ presented a new class of AONs consisting of tricyclo‐DNA (tcDNA) that could be fully absorbed by many tissues after systemic administration in mice characterized by *DMD*. The rescue of dystrophin expression could be promoted by systemic delivery of tcDNA‐AONs in skeletal muscles, heart, and brain, thereby improving physiological cardio‐respiratory functions and correcting behavioral features.

#### 13.2.2 MicroRNA

Previous studies have shown that miRNAs have been implicated as fine regulators in the progression of cardiomyopathy, so miRNAs have become genetic targets for therapy.^401^ Quattrocelli et al.^261^ demonstrated that miR‐669a downregulation linked to the severe DCM progression in Sgcb‐null dystrophic mice, the intraventricular delivery of AAV vectors induced the overexpression of miR‐669a and reduced the mortality of Sgcb‐null mice. miR‐669a treatment could reduce adverse remodeling, enhance systolic fractional shortening of the LV, and ameliorate the gene/miRNA profile of DCM markers in treated dystrophic mice. Apart from the therapeutic target, miRNAs also have the potential to play a role as a biomarker in early disease detection.

#### 13.2.3 Transient receptor potential vaniloid 2

Transient receptor potential vaniloid 2 (TRPV2), a stretch‐sensitive Ca^2+^‐permeable channel, can accumulate and become activated in the sarcolemma of cardiomyocytes/myocytes in cardiomyopathy and MD. The mitigation of muscle dysgenesis may be achieved through TRPV2 inactivation, leading to enhanced cardiac function and improved survival prognosis. While *TRPV2* presents as a promising therapeutic target for cardiomyopathy and MD, research on specific inhibitors is currently underway.^402^

## 14 ISCHEMIC CARDIOMYOPATHY

In developed countries, coronary heart disease continues to be a major contributor to illness and death. Although revascularization strategies, such as coronary artery bypass grafting (CABG), percutaneous coronary intervention, and enhanced medication significantly improve outcomes, approximately 30% of patients still develop chronic heart failure.^403^, ^404^ Ischemic heart disease is characterized by shoddy cardiac remodeling, including cardiac hypertrophy, increased fibrosis, and sparse capillaries. Therefore, gene therapy for ischemic heart disease, such as improving systolic function and promoting new blood vessel formation, appears promising.^405^

### 14.1 Pathogenesis and disease‐causing genes

Ischemic cardiomyopathy (ICM) is primarily characterized by the pathogenesis involving long‐term cardiac ischemia that leads to localized or diffuse myocardial fibrosis, which impairs the heart's contractile and/or diastolic functions, resulting in clinical manifestations such as cardiac dilation or stiffness, congestive heart failure, and arrhythmias. The etiology of ICM is typically associated with atherosclerotic narrowing or occlusion of the coronary arteries; these pathological changes cause an imbalance between the oxygen supply and demand of the myocardium, leading to degeneration, necrosis, myocardial fibrosis, and scar formation of the cardiac muscle cells, and ultimately may lead to heart failure, arrhythmias, and cardiac chamber enlargement.^406^

### 14.2 Potential therapeutic interventions

In acute MI, a single gene therapy application provides continuous therapeutic protein at the site of infarction and may lead to a pathophysiological reversal. Utilizing innovative gene constructs through genetic modification enables the regulation of gene expression based on the intracellular milieu, thereby reducing unrestricted protein synthesis. Stem cell therapy, combined with gene therapy, can promote cardiac regeneration with a high success rate.^407^, ^408^, ^409^ Therefore, gene therapy can improve heart function and relieve symptoms, potentially delaying or reducing the need for heart transplantation.

#### 14.2.1 Vascular endothelial growth factor

VEGF binds to distinct receptors on endothelial cells, playing a vital role in the process of angiogenesis.^410^ The mammalian genome contains five isoforms within the *VEGF* family: *VEGF‐A, VEGF‐B, VEGF‐C, VEGF‐D*, and the *placental growth factor*. Among these, VEGF‐A and VEGF‐B activate VEGF receptor one and VEGF receptor two, thereby governing vascular physiology.^254^, ^411^, ^412^

Transcripts for VEGF‐121 and VEGF‐165 isoforms were found in the majority of cells and tissues expressing the *VEGF* gene. *VEGF‐165* gene therapy has been found to be very effective in promoting angiogenesis.^413^ Gene therapy with *VEGF‐165* is successful in the Kuopio Angiogenesis Assay (KAT trial). In the KAT trial, adenovirus‐mediated *VEGF‐A165* gene therapy showed an improvement in coronary perfusion when IC injections in percutaneous transluminal coronary angioplasty (PTCA) patients with grade 2−3 angina were performed.^414^ The research monitored patients over an 8‐year period, showcasing both safety and effectiveness in individuals with coronary heart disease.^415^ Giusti et al.^138^ performed a Phase I/II prospective and time‐controlled series of clinical trials. Thirteen patients were maintained under optimized clinical management for at least 6 months and then received an IM injection of 2000 µg of plasmid *VEGF‐165*. After treatment, the third month of single‐photon emission computed tomography (SPECT) under stress and the sixth month under rest had a transient increase in myocardial perfusion. One year later, the treadmill test and oxygen consumption of the experimental group improved. For patients with advanced ICM, high‐dose gene therapy has also proven feasible and safe.

In the REVASC trial, patients with severe refractory angina who were not eligible for standard drug therapy were treated with adenovirus for *VEGF‐121* gene therapy by IM injection after mini‐thoracic incision. At 26 weeks of follow‐up, the exercise time before the ischemic change of ECG was prolonged, the total exercise time was increased, and the symptoms of angina were improved.^416^ A clinical study of 31 patients with advanced diffuse coronary artery disease who underwent direct IM injection of AdVEGF121 showed that patients with gene therapy had a low incidence of malignant diseases and retinopathy at a mean follow‐up of 11.8 years. Given the expected prevalence of the age‐matched general population, it was concluded that adenovirus‐mediated *VEGF* could be safely used in the myocardium of patients with severe coronary heart disease. However, conducting trials in a larger population is necessary to assess efficacy.^139^

#### 14.2.2 Granulocyte‐macrophage colony‐stimulating factor

The versatile granulocyte‐macrophage colony‐stimulating factor (GM‐CSF) oversees the production, differentiation, proliferation, and survival of leukocytes.^417^ Although GM‐CSF is not required for hematopoietic function under normal physiological conditions, its yield is significant after myocardial injury. GM‐CSF is the primary coordinator of the white blood cell supply chain during inflammation. It plays a pivotal role in the development of MI and represents a promising therapeutic target.^418^

Seiler et al.^419^ randomized 21 patients who did not meet the CABG criteria but had coronary heart disease who were treated with an IC injection of 40 mg GM‐CSF or placebo and subcutaneous GM‐CSF (10 mg/kg) or placebo respectively for 2 weeks. The invasive collateral flow index of the GM‐CSF group was significantly increased. However, there are still a few contradictory findings. For instance, increased serum levels of GM‐CSF in MI patients correlate with acute decompensated heart failure and subsequent extensive cardiac remodeling,^420^ and administration with exogenous GM‐CSF aggravates heart failure.^421^ These findings suggest that the role of GM‐CSF in MI needs further investigation, and that *GM‐CSF* may become a potential therapeutic target for ICM.

#### 14.2.3 Synthetic‐modified mRNA

Synthetic‐modified mRNA (modRNA) represents a novel gene delivery vector known for its stability, low immunogenicity, and high expression levels, rendering it a promising candidate for treating ICM, particularly post‐MI. After luciferase modRNA was injected IM into the LV, high luciferase activity could be identified within 3 h after injection. The activity reached a peak at 18 h after injection and gradually declined for 6 days. modRNA has been identified as an attractive vector to quickly and efficiently express gene or gene combinations due to its expression kinetics, which could lead to minimizing heart injury and induce regeneration.^422^

## 15 ACQUIRED CARDIOMYOPATH

### 15.1 Tako‐tsubo cardiomyopathy

#### 15.1.1 Pathogenesis

Tako‐tsubo cardiomyopathy (TTC), also known as stress‐induced cardiomyopathy, has a pathogenesis that is not yet fully understood, but it is widely believed to be related to intense emotional or physical stress, involving the massive release of catecholamines.^423^ This release may lead to direct toxic effects on the myocardium, microcirculatory disturbances, and multivessel coronary artery spasm, thereby causing temporary changes in cardiac structure and function. Specifically, catecholamine toxicity is an important factor in the pathogenesis of TTC; elevated levels of catecholamines in the blood of patients may cause microcirculatory disturbances and myocardial stunning.^424^ In addition, metabolic abnormalities in the myocardium and multivessel coronary spasm may also be one of the mechanisms of the disease.

#### 15.1.2 Therapeutic interventions

Because TTC is a self‐limiting condition, treatment mainly targets symptoms, such as using antianxiety medications to alleviate emotional stress and using diuretics, ACE inhibitors, and so on, to treat heart failure.

### 15.2 Peripartum cardiomyopathy

#### 15.2.1 Pathogenesis

Peripartum cardiomyopathy (PPCM) is a form of myocardial disease that emerges during the final month of pregnancy or within the first 5 months postpartum, hallmarked by the onset of LV dysfunction that can range from acute to gradually progressive. While the exact etiology of PPCM remains elusive, it is thought to result from a confluence of contributing factors. The influence of genetic factors in the development of PPCM is underscored by numerous studies, revealing a genetic etiology in as many as 20% of the patients examined.^425^ Truncating mutations in the *TTN*, *DSP*, *FLNC*, and *BAG3* genes have been identified in women with PPCM, with relative prevalence rates (*TTN*: 10%; *DSP* and *FLNC*: 1%; *BAG3*: 0.2%) that are nearly identical to the rates found in the DCM cohort, further supporting a high degree of genetic similarity between PPCM and DCM.^426^ However, it is still unclear how these genetic mutations predispose individuals to PPCM.

#### 15.2.2 Potential therapeutic interventions

Treatment for PPCM typically focuses on symptom management and may include diuretics, angiotensin‐converting enzyme inhibitors, ARBs, beta‐blockers, and anticoagulant drugs. As a dopamine D2 receptor agonist, bromocriptine, which blocks the production of prolactin, has emerged as a potential disease‐specific treatment for PPCM. The 2018 ESC guidelines recommend considering the use of bromocriptine in women newly diagnosed with PPCM.^427^ A recent multicenter randomized study evaluated the effects of two distinct bromocriptine dosing regimens‐2.5 mg daily for 1 week versus 5 mg daily for 2 weeks, followed by a continuation of 2.5 mg daily for 6 weeks—on patients with severe PPCM. The results demonstrated a high rate of LV recovery after 6 months, with zero mortality, no need for assistive devices or heart transplantation. These findings suggest a significant positive correlation between bromocriptine treatment in the acute phase of PPCM and improved clinical outcomes.^136^

### 15.3 Tachycardia‐induced cardiomyopathy

#### 15.3.1 Pathogenesis

Prolonged episodes of tachycardia are a recognized cause of LV systolic dysfunction. Tachycardia‐induced cardiomyopathy is characterized as a heart failure syndrome resulting from the sustained elevation of atrial or ventricular heart rates.^428^ The exact mechanisms of how tachycardia leads to cardiomyopathy are not completely understood.

#### 15.3.2 Potential therapeutic interventions

Treatment for TIC often involves controlling the heart rate and rhythm to prevent further damage and may include medications, catheter ablation, or other procedures to manage the underlying cause of the tachycardia. If the tachycardia is effectively treated, the heart may recover its normal function over time, although this is not guaranteed and depends on the extent of the damage and the individual's response to treatment.

### 15.4 Myocarditis

#### 15.4.1 Pathogenesis

Myocarditis is a prevalent precursor to DCM and SCD, often arising from cardiotropic viral infections that lead to subsequent active inflammatory myocardial damage.^429^ In addition, some noninfectious factors (including toxins, immunological syndromes, and hypersensitivity) may also lead to the occurrence of myocarditis.

#### 15.4.2 Potential therapeutic interventions

IV immunoglobulin is a highly effective immunomodulatory treatment, commonly utilized for patients afflicted with systemic autoimmune diseases characterized by antibody‐mediated pathogenesis, including the particularly aggressive giant‐cell myocarditis.^430^ At present, there are no specific pathogen‐targeted or antiviral treatments approved for patients with viral myocarditis. While the use of aciclovir, ganciclovir, or valaciclovir for herpesvirus infections could be contemplated, their effectiveness has not been explicitly assessed in myocarditis patients.^431^ A pioneering in vitro model replicating human viral myocarditis has been established by exposing hiPSC‐CMs to coxsackievirus. These infected cells exhibit detrimental alterations in cardiomyocyte structure and function, and they demonstrate varied responses to an array of antiviral agents. This hiPSC‐CM model represents a promising tool for investigating the underlying disease mechanisms and serves as a platform for high‐throughput screening of potential new therapies.^432^

## 16 CONCLUSION AND PROSPECTIVE

Cardiomyopathies are a group of heterogeneous diseases characterized by structural and functional impairment of the heart. The pathogenesis of cardiomyopathy is complex and diverse, involving genetic mutations, immune responses, apoptosis, and energy metabolism imbalances, among other aspects. We emphasize the importance of a deep understanding of these mechanisms, which not only helps to identify new therapeutic targets but is also crucial for the development of personalized medical strategies. This review has summarized the pathogenesis of cardiomyopathies, current treatment strategies, and emerging gene and cell therapies. We have gained insights into the molecular mechanisms of cardiomyopathies, including genetic mutations, cell death, fibrosis, and immune responses, providing new perspectives for early diagnosis and treatment. Recent preclinical animal experiments and clinical trials have demonstrated that gene therapy and cell therapy have shown great potential in the treatment of cardiomyopathies, especially for hereditary forms, by targeting pathogenic genes and offering more precise therapeutic directions (Tables 2 and 3).

**TABLE 3:** Major preclinical studies of potential therapy intervention in cardiomyopathies.

Cardiomyopathy	Targeted genes and molecules	Therapeutic intervention	Mechanism of action	References
Hypertrophic cardiomyopathy	*MYBPC3*	Rapamycin	Enhances Akt–mTORC1 signaling	^150^
		AAV‐U7–AON‐5+6	Increases Var‐4 mRNA/protein levels and reduces aberrant mRNAs	^66^
		AAV9–*Mybpc3*	Increasing Mybpc3 mRNA and cMyBP‐C protein levels	^159^
		CRISPR/Cas9	Corrects the variant using HDR	^71^
	*cMyBPC*	AAV9–C0C2	cMyBPC gene transfer	^160^
		Lentiviral vector	cMyBPC gene transfer	^153^
	*MYH7*	CASAAV	Silences *Myh6* and *Myh7* and early depletion of *Myh7*	^162^
		CRISPR/Cas9	Inhibits mTOR or MAPK	^163^
	*MYH6*	RNAi cassette	Silences Myh6 R403Q mutation	^64^
	*PRKAG2*	CRISPR/Cas9	Rectifies heterozygous missense mutation (c.905G>A, R302Q) in the *PRKAG2* gene	^177^
Arrhythmogenic right ventricular cardiomyopathy	*PKP2*	AAVrh.74–PKP2a	Recovers the expression of PKP2a	^193^
	*PLN*	TALEN	Corrects *PLN* R14del mutation	^195^
		AAV9–CRISPR/Cas9	Disruption of hPLN‐R14del allele	^196^
	*DSG2*	HDR‐iPSC	Heterozygously corrects mutated DSG2 gene locus to a normal allele	^199^
Dilated cardiomyopathy	*TNNT2*	AAV9	Heart‐specific delivery overexpression of XINB	^233^
		ASP‐RNAi	Specifically knocks down mutant alleles coding for R92Q and R173W mutant	^234^
		siRNAs	Rescues LMNA‐relate progeria and reduces prelamin A/progerin in favor of the alternative splicing of lamin C.	^235^
		PTC124	Full‐length LMNA proteins were increased by PTC124 treatment and improves the excitation–contraction coupling of the affected cardiomyocytes in the R225X mutant	^238^
	*TTN*	AON	Reframing titin transcripts by AON‐mediated exon skipping	^68^
		CRISPR/Cas9	Ablates A‐band variant‐specific truncation peptide	^244^
	*ASK1*	rAAV	Inhibits ASK1 protein activation	^251^
Restrictive cardiomyopathy	*MYL2*	AAV9–M7.8L R	Silences the mutated allele (RLC‐47K)	^285^
PRKAG2 cardiomyopathy	*PRKAG2*	AAV9–Cas9/sgRNA	Disrupt the mutant PRKAG2 allele encoding H530R while leaving the wild‐type allele intact	^306^
Pompe disease	*GAA*	AAVB1 and AAV9	Combines IGF2 with a codon‐optimized variant of GAA (LV‐IGF2.GAAco) to enhance cellular uptake	^317^
Lamin A/C gene (LMNA) cardiomyopathy	*LMNA*	rAAVs	Whole‐body supplementation with LMNA using rAAVs	^325^
		Temsirolimus	Prevents the hyperactivation of the AKT–mTOR	^329^
Brugada syndrome	*SCN5A*	CRISPR/Cas9	Utilizes two readthrough‐enhancing methods (either aminoglycosides or a siRNA‐targeting eukaryotic release factor eRF3)	^342^
	*MOG1*	AAV9	Enhances MOG1 expression, resulting in augmented ventricular INa levels	^343^
Long QT syndrome	*KCNQ*	SupRep	Introduces a KCNQ1 short hairpin RNA and a short hairpin RNA‐resistant KCNQ1 cDNA into LQT1 iPSC‐CMs.	^351^
Cardiac amyloidosis	*TTR*	RNAi	Reduces unstable cyclic TTR tetramers and Prevents organ deposition of TTR monomers and amyloid fibrils	^377^
		CRISPR/Cas9	specifically knocks out the *TTR* gene	^381^
Muscular dystrophies	*DMD*	AAV9–βARKct–cDNA	Increases cardiac GRK2 activity with βARKct expression	^389^
		CRISPR/Cas9	Removes the premature termination codon and restores the expression of truncated dystrophin through exon skipping	^395^, ^396^, ^397^, ^398^
		Systemic AAV CRISPR therapy	Increases dose of the guide RNA vector in all striated muscles	^394^
		tcDNA‐AONs	Improves physiological cardio‐respiratory functions	^400^

Abbreviations: cMyBPC, cardiac myosin binding protein C; HDR, homology‐directed repair; CASAAV, CRISPR/Cas9‐AAV9‐based somatic mutagenesis; MAPK, mitogen‐activated protein kinase; RNAi, RNA interference; AAVrh.74, adeno‐associated virus vector of serotype rh.74; PKP2a, PKP2 variant A; ATTR‐CA, hereditary amyloidosis; TTR, transthyretin; GRK2, G‐protein‐coupled‐receptor‐kinase‐2; tcDNA, tricyclo‐DNA; ASP‐RNAi, allele‐specific silencing by RNA interference; AON, antisense oligonucleotide; IGF2, insulin‐like growth factor 2; SupRep, suppression‐and‐replacement.

With an enhanced understanding of the molecular mechanisms of cardiomyopathies, future treatments will become more personalized, tailored to the patient's genetic background and disease characteristics. Gene editing technologies such as CRISPR/Cas9, TALENs, and applications of cell therapy like iPSCs offer new strategies for the treatment of cardiomyopathies. Although gene therapies open a new era for genetic diseases, the results have various limitations. For example, most studies have shown that gene therapy can inhibit or delay the progression of cardiomyopathy only for animals born 1 day, and some of the inhibitory effects will gradually disappear over time and cannot reverse the already thickened myocardium.^433^ The challenges in the future would be how to strictly control the effect of the target gene with gene transfer techniques and reduce side effects or excessive expression of exogenous proteins; how to avoid potential immunity to the transgene and viral capsid; how to find a more specific cardiac‐targeted virus, and reduce the uptake of “off‐target” viral vectors by other tissues because incorrect intake may impair the function of different tissues and the carrier of viral vectors. Besides, most animal experiments have been performed in mice or dogs, so the dose of virus injected, the dose‐effect relationship, along with the injection interval in higher mammals need to be further explored.

Cardiac gene therapy is evolving into a replacement for traditional treatments, significant advances have been reported in preclinical models of cardiomyopathy, but technical challenges remain. Therefore, the development of new, more efficient, and smaller gene vectors is particularly important, such as micro circles and mini‐intronic plasmids that exhibit superior transfection efficiency and greater biological effects.^434^ At the same time, exosomes are a new delivery platform for gene therapy. Besides, cell‐containing RNA‐containing exosomes can serve as functional genetic biomarkers for disease.^435^, ^436^, ^437^ While animal models often replicate the clinical features of cardiomyopathy, its crucial to acknowledge the physiological and physical distinctions between these models and humans. Recently, a large number of reports on stem cell therapy have provided us with a better choice for assessing cardiomyopathy in a real clinical setting.^438^, ^439^, ^440^ Stem cell therapy has been successfully used in small studies of ischemic, dilated, and RCM. In the near future, stem‐cell‐based gene therapy and cell therapy might be an effective way for cardiomyopathy treatment.

In summary, the research and treatment of cardiomyopathy are in a rapidly advancing phase. Through interdisciplinary collaboration and the application of innovative technologies, we have reason to believe that in the future, we will be able to more effectively prevent, diagnose, and treat cardiomyopathy, thereby improving the quality of life for patients.

## AUTHOR CONTRIBUTIONS

Abdelouahab Bellou, Jian Zhuang, and Liming Lei conceived the manuscript. Shitong Huang, Qiuying Li, and Jiaxin Li wrote the initial draft of the manuscript. Shitong Huang drew figures. Xianwu Zhou, Xuanhui Chen, and Jimei Chen participated in the revision of the manuscript. All authors have read and agreed on the submission a publication of this manuscript.

## CONFLICT OF INTEREST STATEMENT

The authors declare no conflict of interest.

## ETHICS STATEMENT

This systematic review adhered to the ethical guidelines for research and publication as outlined by the Guangdong Provincial People's Hospital. Since this review did not involve the collection or analysis of primary data, no formal ethics approval was required. However, the authors ensured that all data sources used in this review were obtained and analyzed in accordance with the principles of ethical research.

## Data Availability

Data availability is not applicable to this article as no new data were created or analyzed in this study.

## References

[1] Lipshultz SE , Law YM , Asante‐Korang A , et al. Cardiomyopathy in Children: Classification and Diagnosis: A Scientific Statement From the American Heart Association. Circulation. 2019;140(1):e9‐e68. 31132865 10.1161/CIR.0000000000000682

[2] Dadson K , Hauck L , Billia F . Molecular mechanisms in cardiomyopathy. Clin Sci (Lond). 2017;131(13):1375‐1392. 28645928 10.1042/CS20160170

[3] Maron BJ , Towbin JA , Thiene G , et al. Contemporary definitions and classification of the cardiomyopathies: an American Heart Association Scientific Statement from the Council on Clinical Cardiology, Heart Failure and Transplantation Committee; Quality of Care and Outcomes Research and Functional Genomics and Translational Biology Interdisciplinary Working Groups; and Council on Epidemiology and Prevention. Circulation. 2006;113(14):1807‐1816. 16567565 10.1161/CIRCULATIONAHA.106.174287

[4] Strong A , Musunuru K . Genome editing in cardiovascular diseases. Nat Rev Cardiol. 2017;14(1):11‐20. 27609628 10.1038/nrcardio.2016.139

[5] Tan K , Foo R , Loh M . Cardiomyopathy in Asian Cohorts: Genetic and Epigenetic Insights. Circ Genom Precis Med. 2023;16(5):496‐506. 37589150 10.1161/CIRCGEN.123.004079

[6] Hershberger RE , Givertz MM , Ho CY , et al. Genetic Evaluation of Cardiomyopathy‐A Heart Failure Society of America Practice Guideline. J Card Fail. 2018;24(5):281‐302. 29567486 10.1016/j.cardfail.2018.03.004 PMC9903357

[7] Yamada T , Nomura S . Recent Findings Related to Cardiomyopathy and Genetics. Int J Mol Sci. 2021;22(22):12522. 34830403 10.3390/ijms222212522 PMC8623065

[8] Argiro A , Bui Q , Hong KN , Ammirati E , Olivotto I , Adler E . Applications of Gene Therapy in Cardiomyopathies. JACC Heart Fail. 2024;12(2):248‐260. 37966402 10.1016/j.jchf.2023.09.015

[9] Teekakirikul P , Zhu W , Huang HC , Fung E . Hypertrophic Cardiomyopathy: An Overview of Genetics and Management. Biomolecules. 2019;9(12):878. 31888115 10.3390/biom9120878 PMC6995589

[10] Tsoutsman T , Lam L , Semsarian C . Genes, calcium and modifying factors in hypertrophic cardiomyopathy. Clin Exp Pharmacol Physiol. 2006;33(1‐2):139‐145. 16445713 10.1111/j.1440-1681.2006.04340.x

[11] Lopes LR , Garcia‐Hernández S , Lorenzini M , et al. Alpha‐protein kinase 3 (ALPK3) truncating variants are a cause of autosomal dominant hypertrophic cardiomyopathy. Eur Heart J. 2021;42(32):3063‐3073. 34263907 10.1093/eurheartj/ehab424 PMC8380059

[12] Landstrom AP , Ackerman MJ . Mutation type is not clinically useful in predicting prognosis in hypertrophic cardiomyopathy. Circulation. 2010;122(23):2441‐2449. discussion 2450. 21135372 10.1161/CIRCULATIONAHA.110.954446 PMC6309993

[13] Alimadadi A , Munroe PB , Joe B , Cheng X . Meta‐Analysis of Dilated Cardiomyopathy Using Cardiac RNA‐Seq Transcriptomic Datasets. Genes (Basel). 2020;11(1):60. 31948008 10.3390/genes11010060 PMC7017089

[14] Asimaki A , Tandri H , Huang H , et al. A new diagnostic test for arrhythmogenic right ventricular cardiomyopathy. N Engl J Med. 2009;360(11):1075‐1084. 19279339 10.1056/NEJMoa0808138

[15] Pilichou K , Nava A , Basso C , et al. Mutations in desmoglein‐2 gene are associated with arrhythmogenic right ventricular cardiomyopathy. Circulation. 2006;113(9):1171‐1179. 16505173 10.1161/CIRCULATIONAHA.105.583674

[16] Corrado D , Basso C , Pilichou K , Thiene G . Molecular biology and clinical management of arrhythmogenic right ventricular cardiomyopathy/dysplasia. Heart. 2011;97(7):530‐539. 20930047 10.1136/hrt.2010.193276

[17] Caleshu C , Sakhuja R , Nussbaum RL , et al. Furthering the link between the sarcomere and primary cardiomyopathies: restrictive cardiomyopathy associated with multiple mutations in genes previously associated with hypertrophic or dilated cardiomyopathy. Am J Med Genet A. 2011;155a(9):2229‐2235. 21823217 10.1002/ajmg.a.34097 PMC3158811

[18] Luedde M , Ehlermann P , Weichenhan D , et al. Severe familial left ventricular non‐compaction cardiomyopathy due to a novel troponin T (TNNT2) mutation. Cardiovasc Res. 2010;86(3):452‐460. 20083571 10.1093/cvr/cvq009

[19] Verdonschot JAJ , Hazebroek MR , Krapels IPC , et al. Implications of Genetic Testing in Dilated Cardiomyopathy. Circ Genom Precis Med. 2020;13(5):476‐487. 32880476 10.1161/CIRCGEN.120.003031

[20] Cirino AL , Seidman CE , Ho CY . Genetic Testing and Counseling for Hypertrophic Cardiomyopathy. Cardiol Clin. 2019;37(1):35‐43. 30447714 10.1016/j.ccl.2018.08.003

[21] Kubo T , Kitaoka H . Genetic Testing for Cardiomyopathy in Japan 2022: Current Status and Issues of Precision Medicine. J Card Fail. 2023;29(5):805‐814. 37169422 10.1016/j.cardfail.2022.11.017

[22] Chiswell K , Zaininger L , Semsarian C . Evolution of genetic testing and gene therapy in hypertrophic cardiomyopathy. Prog Cardiovasc Dis. 2023;80:38‐45. 37137376 10.1016/j.pcad.2023.04.009

[23] Ahluwalia M , Ho CY . Cardiovascular genetics: the role of genetic testing in diagnosis and management of patients with hypertrophic cardiomyopathy. Heart. 2021;107(3):183‐189. 33172912 10.1136/heartjnl-2020-316798

[24] Gaine SP , Calkins H . Antiarrhythmic Drug Therapy in Arrhythmogenic Right Ventricular Cardiomyopathy. Biomedicines. 2023;11(4):1213. 37189831 10.3390/biomedicines11041213 PMC10136163

[25] Cappelli F , Morini S , Pieragnoli P , et al. Cardiac Resynchronization Therapy for End‐Stage Hypertrophic Cardiomyopathy: The Need for Disease‐Specific Criteria. Journal of the American College of Cardiology. 2018;71(4):464‐466. 29389365 10.1016/j.jacc.2017.11.040

[26] Sidhu K , Castrini AI , Parikh V , et al. The response to cardiac resynchronization therapy in LMNA cardiomyopathy. Eur J Heart Fail. 2022;24(4):685‐693. 35229420 10.1002/ejhf.2463 PMC9106891

[27] Daubert JP , Barnett AS . Primary Prevention Implantable Cardioverter‐Defibrillators in Patients With Nonischemic Cardiomyopathy. JACC Heart Fail. 2019;7(8):725‐727. 31302053 10.1016/j.jchf.2019.05.011

[28] Stefàno P , Argirò A , Bacchi B , et al. Does a standard myectomy exist for obstructive hypertrophic cardiomyopathy? From the Morrow variations to precision surgery. Int J Cardiol. 2023;371:278‐286. 36130619 10.1016/j.ijcard.2022.09.036

[29] Bogle C , Colan SD , Miyamoto SD , et al. Treatment Strategies for Cardiomyopathy in Children: A Scientific Statement From the American Heart Association. Circulation. 2023;148(2):174‐195. 37288568 10.1161/CIR.0000000000001151

[30] Callis TE , Jensen BC , Weck KE , Willis MS . Evolving molecular diagnostics for familial cardiomyopathies: at the heart of it all. Expert Rev Mol Diagn. 2010;10(3):329‐351. 20370590 10.1586/erm.10.13 PMC5022563

[31] Li Z , Chen P , Li C , et al. Genetic arrhythmias complicating patients with dilated cardiomyopathy. Heart Rhythm. 2020;17(2):305‐312. 31521807 10.1016/j.hrthm.2019.09.012

[32] Gacita AM , Fullenkamp DE , Ohiri J , et al. Genetic Variation in Enhancers Modifies Cardiomyopathy Gene Expression and Progression. Circulation. 2021;143(13):1302‐1316. 33478249 10.1161/CIRCULATIONAHA.120.050432 PMC8009836

[33] Zacchigna S , Zentilin L , Giacca M . Adeno‐associated virus vectors as therapeutic and investigational tools in the cardiovascular system. Circ Res. 2014;114(11):1827‐1846. 24855205 10.1161/CIRCRESAHA.114.302331

[34] Salman OF , El‐Rayess HM , Abi Khalil C , Nemer G , Refaat MM . Inherited Cardiomyopathies and the Role of Mutations in Non‐coding Regions of the Genome. Front Cardiovasc Med. 2018;5:77. 29998127 10.3389/fcvm.2018.00077 PMC6028572

[35] Ashrafian H , Watkins H . Reviews of translational medicine and genomics in cardiovascular disease: new disease taxonomy and therapeutic implications cardiomyopathies: therapeutics based on molecular phenotype. J Am Coll Cardiol. 2007;49(12):1251‐1264. 17394955 10.1016/j.jacc.2006.10.073

[36] Ferrua F , Cicalese MP , Galimberti S , et al. Lentiviral haemopoietic stem/progenitor cell gene therapy for treatment of Wiskott‐Aldrich syndrome: interim results of a non‐randomised, open‐label, phase 1/2 clinical study. Lancet Haematol. 2019;6(5):e239‐e253. 30981783 10.1016/S2352-3026(19)30021-3 PMC6494976

[37] Sessa M , Lorioli L , Fumagalli F , et al. Lentiviral haemopoietic stem‐cell gene therapy in early‐onset metachromatic leukodystrophy: an ad‐hoc analysis of a non‐randomised, open‐label, phase 1/2 trial. Lancet. 2016;388(10043):476‐487. 27289174 10.1016/S0140-6736(16)30374-9

[38] Aiuti A , Biasco L , Scaramuzza S , et al. Lentiviral hematopoietic stem cell gene therapy in patients with Wiskott‐Aldrich syndrome. Science. 2013;341(6148):1233151. 23845947 10.1126/science.1233151 PMC4375961

[39] De Ravin SS , Wu X , Moir S , et al. Lentiviral hematopoietic stem cell gene therapy for X‐linked severe combined immunodeficiency. Sci Transl Med. 2016;8(335):335ra57. 10.1126/scitranslmed.aad8856 PMC5557273 27099176

[40] Campochiaro PA , Lauer AK , Sohn EH , et al. Lentiviral Vector Gene Transfer of Endostatin/Angiostatin for Macular Degeneration (GEM) Study. Hum Gene Ther. 2017;28(1):99‐111. 27710144 10.1089/hum.2016.117 PMC5278797

[41] Villanueva MT . Gene therapy: Gene therapy before the cradle. Nat Rev Drug Discov. 2018;17(9):619. 30116052 10.1038/nrd.2018.140

[42] Naso MF , Tomkowicz B , Perry WL, 3rd , Strohl WR . Adeno‐Associated Virus (AAV) as a Vector for Gene Therapy. BioDrugs. 2017;31(4):317‐334. 28669112 10.1007/s40259-017-0234-5 PMC5548848

[43] Lodola F , Morone D , Denegri M , et al. Adeno‐associated virus‐mediated CASQ2 delivery rescues phenotypic alterations in a patient‐specific model of recessive catecholaminergic polymorphic ventricular tachycardia. Cell Death Dis. 2016;7(10):e2393. 27711080 10.1038/cddis.2016.304 PMC5133973

[44] Guo Y , VanDusen NJ , Zhang L , et al. Analysis of Cardiac Myocyte Maturation Using CASAAV, a Platform for Rapid Dissection of Cardiac Myocyte Gene Function In Vivo. Circ Res. 2017;120(12):1874‐1888. 28356340 10.1161/CIRCRESAHA.116.310283 PMC5466492

[45] Boutin S , Monteilhet V , Veron P , et al. Prevalence of serum IgG and neutralizing factors against adeno‐associated virus (AAV) types 1, 2, 5, 6, 8, and 9 in the healthy population: implications for gene therapy using AAV vectors. Hum Gene Ther. 2010;21(6):704‐712. 20095819 10.1089/hum.2009.182

[46] Hirata R , Chamberlain J , Dong R , Russell DW . Targeted transgene insertion into human chromosomes by adeno‐associated virus vectors. Nat Biotechnol. 2002;20(7):735‐738. 12089561 10.1038/nbt0702-735

[47] Wang S , Li Y , Xu Y , et al. AAV Gene Therapy Prevents and Reverses Heart Failure in a Murine Knockout Model of Barth Syndrome. Circ Res. 2020;126(8):1024‐1039. 32146862 10.1161/CIRCRESAHA.119.315956 PMC7233109

[48] Werfel S , Jungmann A , Lehmann L , et al. Rapid and highly efficient inducible cardiac gene knockout in adult mice using AAV‐mediated expression of Cre recombinase. Cardiovasc Res. 2014;104(1):15‐23. 25082846 10.1093/cvr/cvu174

[49] Ishikawa K , Weber T , Hajjar RJ . Human Cardiac Gene Therapy. Circ Res. 2018;123(5):601‐613. 30355138 10.1161/CIRCRESAHA.118.311587 PMC6390977

[50] Nguyen GN , Everett JK , Kafle S , et al. A long‐term study of AAV gene therapy in dogs with hemophilia A identifies clonal expansions of transduced liver cells. Nat Biotechnol. 2021;39(1):47‐55. 33199875 10.1038/s41587-020-0741-7 PMC7855056

[51] Mullard A . Gene therapy community grapples with toxicity issues, as pipeline matures. Nat Rev Drug Discov. 2021;20(11):804‐805. 34599291 10.1038/d41573-021-00164-x

[52] Vekstein AM , Wendell DC , DeLuca S , et al. Targeted Delivery for Cardiac Regeneration: Comparison of Intra‐coronary Infusion and Intra‐myocardial Injection in Porcine Hearts. Front Cardiovasc Med. 2022;9:833335. 35224061 10.3389/fcvm.2022.833335 PMC8866722

[53] Chung ES , Miller L , Patel AN , et al. Changes in ventricular remodelling and clinical status during the year following a single administration of stromal cell‐derived factor‐1 non‐viral gene therapy in chronic ischaemic heart failure patients: the STOP‐HF randomized Phase II trial. Eur Heart J. 2015;36(33):2228‐2238. 26056125 10.1093/eurheartj/ehv254 PMC4554960

[54] Milone MC , O'Doherty U . Clinical use of lentiviral vectors. Leukemia. 2018;32(7):1529‐1541. 29654266 10.1038/s41375-018-0106-0 PMC6035154

[55] Zsebo K , Yaroshinsky A , Rudy JJ , et al. Long‐term effects of AAV1/SERCA2a gene transfer in patients with severe heart failure: analysis of recurrent cardiovascular events and mortality. Circ Res. 2014;114(1):101‐108. 24065463 10.1161/CIRCRESAHA.113.302421

[56] Greenberg B , Butler J , Felker GM , et al. Calcium upregulation by percutaneous administration of gene therapy in patients with cardiac disease (CUPID 2): a randomised, multinational, double‐blind, placebo‐controlled, phase 2b trial. Lancet. 2016;387(10024):1178‐1186. 26803443 10.1016/S0140-6736(16)00082-9

[57] Boekstegers P , von Degenfeld G , Giehrl W , et al. Myocardial gene transfer by selective pressure‐regulated retroinfusion of coronary veins. Gene Ther. 2000;7(3):232‐240. 10694800 10.1038/sj.gt.3301079

[58] Boekstegers P , Kupatt C . Current concepts and applications of coronary venous retroinfusion. Basic Res Cardiol. 2004;99(6):373‐381. 15503084 10.1007/s00395-004-0486-3

[59] Salami CO , Jackson K , Jose C , et al. Stress‐Induced Mouse Model of the Cardiac Manifestations of Friedreich's Ataxia Corrected by AAV‐mediated Gene Therapy. Hum Gene Ther. 2020;31(15‐16):819‐827. 32646255 10.1089/hum.2019.363

[60] Kevany BM , Padegimas L , Miller TJ . A novel AAV capsid with improved CNS tropism for treating Pompe disease by intravenous administration. Molecular Genetics and Metabolism. 2019;126(2):S83.

[61] Vassalli G , Büeler H , Dudler J , von Segesser LK , Kappenberger L . Adeno‐associated virus (AAV) vectors achieve prolonged transgene expression in mouse myocardium and arteries in vivo: a comparative study with adenovirus vectors. Int J Cardiol. 2003;90(2‐3):229‐238. 12957756 10.1016/s0167-5273(02)00554-5

[62] Mearini G , Stimpel D , Krämer E , et al. Repair of Mybpc3 mRNA by 5'‐trans‐splicing in a Mouse Model of Hypertrophic Cardiomyopathy. Mol Ther Nucleic Acids. 2013;2(7):e102. 23820890 10.1038/mtna.2013.31 PMC3731888

[63] Wally V , Murauer EM , Bauer JW . Spliceosome‐mediated trans‐splicing: the therapeutic cut and paste. J Invest Dermatol. 2012;132(8):1959‐1966. 22495179 10.1038/jid.2012.101

[64] Jiang J , Wakimoto H , Seidman JG , Seidman CE . Allele‐specific silencing of mutant Myh6 transcripts in mice suppresses hypertrophic cardiomyopathy. Science. 2013;342(6154):111‐114. 24092743 10.1126/science.1236921 PMC4100553

[65] Bongianino R , Denegri M , Mazzanti A , et al. Allele‐Specific Silencing of Mutant mRNA Rescues Ultrastructural and Arrhythmic Phenotype in Mice Carriers of the R4496C Mutation in the Ryanodine Receptor Gene (RYR2). Circ Res. 2017;121(5):525‐536. 28620067 10.1161/CIRCRESAHA.117.310882

[66] Gedicke‐Hornung C , Behrens‐Gawlik V , Reischmann S , et al. Rescue of cardiomyopathy through U7snRNA‐mediated exon skipping in Mybpc3‐targeted knock‐in mice. EMBO Mol Med. 2013;5(7):1128‐1145. 23716398 10.1002/emmm.201202168 PMC3721478

[67] Hahn JK , Neupane B , Pradhan K , et al. The assembly and evaluation of antisense oligonucleotides applied in exon skipping for titin‐based mutations in dilated cardiomyopathy. J Mol Cell Cardiol. 2019;131:12‐19. 30998980 10.1016/j.yjmcc.2019.04.014

[68] Gramlich M , Pane LS , Zhou Q , et al. Antisense‐mediated exon skipping: a therapeutic strategy for titin‐based dilated cardiomyopathy. EMBO Mol Med. 2015;7(5):562‐576. 25759365 10.15252/emmm.201505047 PMC4492817

[69] West SC . Molecular views of recombination proteins and their control. Nat Rev Mol Cell Biol. 2003;4(6):435‐445. 12778123 10.1038/nrm1127

[70] He X , Du T , Long T , Liao X , Dong Y , Huang ZP . Signaling cascades in the failing heart and emerging therapeutic strategies. Signal Transduct Target Ther. 2022;7(1):134. 35461308 10.1038/s41392-022-00972-6 PMC9035186

[71] Nie J , Han Y , Jin Z , et al. Homology‐directed repair of an MYBPC3 gene mutation in a rat model of hypertrophic cardiomyopathy. Gene Ther. 2023;30(6):520‐527. 36765144 10.1038/s41434-023-00384-3

[72] Cox DB , Platt RJ , Zhang F . Therapeutic genome editing: prospects and challenges. Nat Med. 2015;21(2):121‐131. 25654603 10.1038/nm.3793 PMC4492683

[73] Vermersch E , Jouve C , Hulot JS . CRISPR/Cas9 gene‐editing strategies in cardiovascular cells. Cardiovasc Res. 2020;116(5):894‐907. 31584620 10.1093/cvr/cvz250

[74] Rezaei H , Khadempar S , Farahani N , et al. Harnessing CRISPR/Cas9 technology in cardiovascular disease. Trends Cardiovasc Med. 2020;30(2):93‐101. 30935726 10.1016/j.tcm.2019.03.005

[75] Motta BM , Pramstaller PP , Hicks AA , Rossini A . The Impact of CRISPR/Cas9 Technology on Cardiac Research: From Disease Modelling to Therapeutic Approaches. Stem Cells Int. 2017;2017:8960236. 29434642 10.1155/2017/8960236 PMC5757142

[76] Mehta A , Merkel OM . Immunogenicity of Cas9 Protein. J Pharm Sci. 2020;109(1):62‐67. 31589876 10.1016/j.xphs.2019.10.003 PMC7115921

[77] Zhang M , Wang F , Li S , Wang Y , Bai Y , Xu X . TALE: a tale of genome editing. Prog Biophys Mol Biol. 2014;114(1):25‐32. 24291598 10.1016/j.pbiomolbio.2013.11.006

[78] Dimitrov AS . Methods in molecular biology. Therapeutic antibodies. Methods and protocols. Preface. Methods Mol Biol. 2009;525:vii‐viii, xiii. 19350746 10.1007/978-1-59745-554-1

[79] Joung JK , Sander JD . TALENs: a widely applicable technology for targeted genome editing. Nat Rev Mol Cell Biol. 2013;14(1):49‐55. 23169466 10.1038/nrm3486 PMC3547402

[80] Karakikes I , Termglinchan V , Cepeda DA , et al. A Comprehensive TALEN‐Based Knockout Library for Generating Human‐Induced Pluripotent Stem Cell‐Based Models for Cardiovascular Diseases. Circ Res. 2017;120(10):1561‐1571. 28246128 10.1161/CIRCRESAHA.116.309948 PMC5429194

[81] Schreurs J , Sacchetto C , Colpaert RMW , Vitiello L , Rampazzo A , Calore M . Recent Advances in CRISPR/Cas9‐Based Genome Editing Tools for Cardiac Diseases. Int J Mol Sci. 2021;22(20):10985. 34681646 10.3390/ijms222010985 PMC8537312

[82] Hirakawa MP , Krishnakumar R , Timlin JA , Carney JP , Butler KS . Gene editing and CRISPR in the clinic: current and future perspectives. Biosci Rep. 2020;40(4):BSR20200127. 32207531 10.1042/BSR20200127 PMC7146048

[83] Chen K , Gao C . TALENs: customizable molecular DNA scissors for genome engineering of plants. J Genet Genomics. 2013;40(6):271‐279. 23790626 10.1016/j.jgg.2013.03.009

[84] Zhang HX , Zhang Y , Yin H . Genome Editing with mRNA Encoding ZFN, TALEN, and Cas9. Mol Ther. 2019;27(4):735‐746. 30803822 10.1016/j.ymthe.2019.01.014 PMC6453514

[85] Yoshida Y , Yamanaka S . Induced Pluripotent Stem Cells 10 Years Later: For Cardiac Applications. Circ Res. 2017;120(12):1958‐1968. 28596174 10.1161/CIRCRESAHA.117.311080

[86] Chong JJ , Yang X , Don CW , et al. Human embryonic‐stem‐cell‐derived cardiomyocytes regenerate non‐human primate hearts. Nature. 2014;510(7504):273‐277. 24776797 10.1038/nature13233 PMC4154594

[87] Ong SG , Huber BC , Lee WH , et al. Microfluidic Single‐Cell Analysis of Transplanted Human Induced Pluripotent Stem Cell‐Derived Cardiomyocytes After Acute Myocardial Infarction. Circulation. 2015;132(8):762‐771. 26304668 10.1161/CIRCULATIONAHA.114.015231 PMC4557214

[88] Tohyama S , Fukuda K . Safe and Effective Cardiac Regenerative Therapy With Human‐Induced Pluripotent Stem Cells: How Should We Prepare Pure Cardiac Myocytes? Circ Res. 2017;120(10):1558‐1560. 28495993 10.1161/CIRCRESAHA.116.310328

[89] Jung JH , Fu X , Yang PC . Exosomes Generated From iPSC‐Derivatives: New Direction for Stem Cell Therapy in Human Heart Diseases. Circ Res. 2017;120(2):407‐417. 28104773 10.1161/CIRCRESAHA.116.309307 PMC5260934

[90] Tachibana A , Santoso MR , Mahmoudi M , et al. Paracrine Effects of the Pluripotent Stem Cell‐Derived Cardiac Myocytes Salvage the Injured Myocardium. Circ Res. 2017;121(6):e22‐e36. 28743804 10.1161/CIRCRESAHA.117.310803 PMC5783162

[91] Song Y , Zheng Z , Lian J . Deciphering Common Long QT Syndrome Using CRISPR/Cas9 in Human‐Induced Pluripotent Stem Cell‐Derived Cardiomyocytes. Front Cardiovasc Med. 2022;9:889519. 35647048 10.3389/fcvm.2022.889519 PMC9136094

[92] Brieler J , Breeden MA , Tucker J . Cardiomyopathy: An Overview. Am Fam Physician. 2017;96(10):640‐646. 29431384

[93] Maron BJ , Haas TS , Ahluwalia A , Murphy CJ , Garberich RF . Demographics and Epidemiology of Sudden Deaths in Young Competitive Athletes: From the United States National Registry. Am J Med. 2016;129(11):1170‐1177. 27039955 10.1016/j.amjmed.2016.02.031

[94] Maron BJ , Haas TS , Murphy CJ , Ahluwalia A , Rutten‐Ramos S . Incidence and causes of sudden death in U.S. college athletes. J Am Coll Cardiol. 2014;63(16):1636‐1643. 24583295 10.1016/j.jacc.2014.01.041

[95] Tuohy CV , Kaul S , Song HK , Nazer B , Heitner SB . Hypertrophic cardiomyopathy: the future of treatment. Eur J Heart Fail. 2020;22(2):228‐240. 31919938 10.1002/ejhf.1715

[96] Walsh R , Mazzarotto F , Whiffin N , et al. Quantitative approaches to variant classification increase the yield and precision of genetic testing in Mendelian diseases: the case of hypertrophic cardiomyopathy. Genome Med. 2019;11(1):5. 30696458 10.1186/s13073-019-0616-z PMC6350371

[97] Elliott PM , Anastasakis A , Borger MA , et al. 2014 ESC Guidelines on diagnosis and management of hypertrophic cardiomyopathy: the Task Force for the Diagnosis and Management of Hypertrophic Cardiomyopathy of the European Society of Cardiology (ESC). Eur Heart J. 2014;35(39):2733‐2779. 25173338 10.1093/eurheartj/ehu284

[98] van Velzen HG , Schinkel AFL , Baart SJ , et al. Outcomes of Contemporary Family Screening in Hypertrophic Cardiomyopathy. Circ Genom Precis Med. 2018;11(4):e001896. 29661763 10.1161/CIRCGEN.117.001896

[99] Jensen MK , Havndrup O , Christiansen M , et al. Penetrance of hypertrophic cardiomyopathy in children and adolescents: a 12‐year follow‐up study of clinical screening and predictive genetic testing. Circulation. 2013;127(1):48‐54. 23197161 10.1161/CIRCULATIONAHA.111.090514

[100] Lopes LR , Zekavati A , Syrris P , et al. Genetic complexity in hypertrophic cardiomyopathy revealed by high‐throughput sequencing. J Med Genet. 2013;50(4):228‐239. 23396983 10.1136/jmedgenet-2012-101270 PMC3607113

[101] Harris SP , Lyons RG , Bezold KL . In the thick of it: HCM‐causing mutations in myosin binding proteins of the thick filament. Circ Res. 2011;108(6):751‐764. 21415409 10.1161/CIRCRESAHA.110.231670 PMC3076008

[102] Nag S , Trivedi DV , Sarkar SS , et al. The myosin mesa and the basis of hypercontractility caused by hypertrophic cardiomyopathy mutations. Nat Struct Mol Biol. 2017;24(6):525‐533. 28481356 10.1038/nsmb.3408 PMC5737966

[103] Witjas‐Paalberends ER , Güçlü A , Germans T , et al. Gene‐specific increase in the energetic cost of contraction in hypertrophic cardiomyopathy caused by thick filament mutations. Cardiovasc Res. 2014;103(2):248‐257. 24835277 10.1093/cvr/cvu127

[104] Ranjbarvaziri S , Kooiker KB , Ellenberger M , et al. Altered Cardiac Energetics and Mitochondrial Dysfunction in Hypertrophic Cardiomyopathy. Circulation. 2021;144(21):1714‐1731. 34672721 10.1161/CIRCULATIONAHA.121.053575 PMC8608736

[105] van Tintelen JP , Entius MM , Bhuiyan ZA , et al. Plakophilin‐2 mutations are the major determinant of familial arrhythmogenic right ventricular dysplasia/cardiomyopathy. Circulation. 2006;113(13):1650‐1658. 16567567 10.1161/CIRCULATIONAHA.105.609719

[106] Ye JZ , Delmar M , Lundby A , Olesen MS . Reevaluation of genetic variants previously associated with arrhythmogenic right ventricular cardiomyopathy integrating population‐based cohorts and proteomics data. Clin Genet. 2019;96(6):506‐514. 31402444 10.1111/cge.13621

[107] Lippi M , Chiesa M , Ascione C , et al. Spectrum of Rare and Common Genetic Variants in Arrhythmogenic Cardiomyopathy Patients. Biomolecules. 2022;12(8):1043. 36008935 10.3390/biom12081043 PMC9405889

[108] Asatryan B , Asimaki A , Landstrom AP , et al. Inflammation and Immune Response in Arrhythmogenic Cardiomyopathy: State‐of‐the‐Art Review. Circulation. 2021;144(20):1646‐1655. 34780255 10.1161/CIRCULATIONAHA.121.055890 PMC9034711

[109] Odak M , Douedi S , Mararenko A , et al. Arrhythmogenic Right Ventricular Cardiomyopathy: The Role of Genetics in Diagnosis, Management, and Screening. Cardiol Res. 2022;13(4):177‐184. 36128418 10.14740/cr1373 PMC9451588

[110] Weissler‐Snir A , Hindieh W , Gruner C , et al. Lack of Phenotypic Differences by Cardiovascular Magnetic Resonance Imaging in MYH7 (β‐Myosin Heavy Chain)‐ Versus MYBPC3 (Myosin‐Binding Protein C)‐Related Hypertrophic Cardiomyopathy. Circ Cardiovasc Imaging. 2017;10(2):e005311. 28193612 10.1161/CIRCIMAGING.116.005311

[111] Repetti GG , Toepfer CN , Seidman JG , Seidman CE . Novel Therapies for Prevention and Early Treatment of Cardiomyopathies. Circ Res. 2019;124(11):1536‐1550. 31120825 10.1161/CIRCRESAHA.119.313569 PMC7092753

[112] Mearini G , Stimpel D , Geertz B , et al. Mybpc3 gene therapy for neonatal cardiomyopathy enables long‐term disease prevention in mice. Nat Commun. 2014;5:5515. 25463264 10.1038/ncomms6515

[113] Adalsteinsdottir B , Teekakirikul P , Maron BJ , et al. Nationwide study on hypertrophic cardiomyopathy in Iceland: evidence of a MYBPC3 founder mutation. Circulation. 2014;130(14):1158‐1167. 25078086 10.1161/CIRCULATIONAHA.114.011207

[114] Montag J , Petersen B , Flögel AK , et al. Successful knock‐in of Hypertrophic Cardiomyopathy‐mutation R723G into the MYH7 gene mimics HCM pathology in pigs. Sci Rep. 2018;8(1):4786. 29555974 10.1038/s41598-018-22936-z PMC5859159

[115] Coto E , Reguero JR , Palacín M , et al. Resequencing the whole MYH7 gene (including the intronic, promoter, and 3' UTR sequences) in hypertrophic cardiomyopathy. J Mol Diagn. 2012;14(5):518‐524. 22765922 10.1016/j.jmoldx.2012.04.001

[116] Singh A , Kukreti S . A triple stranded G‐quadruplex formation in the promoter region of human myosin β(Myh7) gene. J Biomol Struct Dyn. 2018;36(11):2773‐2786. 28927343 10.1080/07391102.2017.1374211

[117] Lee SP , Ashley EA , Homburger J , et al. Incident Atrial Fibrillation Is Associated With MYH7 Sarcomeric Gene Variation in Hypertrophic Cardiomyopathy. Circ Heart Fail. 2018;11(9):e005191. 30354366 10.1161/CIRCHEARTFAILURE.118.005191

[118] Castellana S , Mastroianno S , Palumbo P , et al. Sudden death in mild hypertrophic cardiomyopathy with compound DSG2/DSC2/MYH6 mutations: Revisiting phenotype after genetic assessment in a master runner athlete. J Electrocardiol. 2019;53:95‐99. 30716529 10.1016/j.jelectrocard.2019.01.002

[119] Pua CJ , Tham N , Chin CWL , et al. Genetic Studies of Hypertrophic Cardiomyopathy in Singaporeans Identify Variants in TNNI3 and TNNT2 That Are Common in Chinese Patients. Circ Genom Precis Med. 2020;13(5):424‐434. 32815737 10.1161/CIRCGEN.119.002823 PMC7676617

[120] Wu G , Liu L , Zhou Z , et al. East Asian‐Specific Common Variant in TNNI3 Predisposes to Hypertrophic Cardiomyopathy. Circulation. 2020;142(21):2086‐2089. 33078954 10.1161/CIRCULATIONAHA.120.050384

[121] Olivotto I , Girolami F , Sciagrà R , et al. Microvascular function is selectively impaired in patients with hypertrophic cardiomyopathy and sarcomere myofilament gene mutations. J Am Coll Cardiol. 2011;58(8):839‐848. 21835320 10.1016/j.jacc.2011.05.018

[122] Girolami F , Ho CY , Semsarian C , et al. Clinical features and outcome of hypertrophic cardiomyopathy associated with triple sarcomere protein gene mutations. J Am Coll Cardiol. 2010;55(14):1444‐1453. 20359594 10.1016/j.jacc.2009.11.062

[123] Kimura A . Molecular genetics and pathogenesis of cardiomyopathy. J Hum Genet. 2016;61(1):41‐50. 26178429 10.1038/jhg.2015.83

[124] Wang H , Li Z , Wang J , et al. Mutations in NEXN, a Z‐disc gene, are associated with hypertrophic cardiomyopathy. Am J Hum Genet. 2010;87(5):687‐693. 20970104 10.1016/j.ajhg.2010.10.002 PMC2978958

[125] Gallego‐Delgado M , Gonzalez‐Lopez E , Garcia‐Guereta L , et al. Adverse clinical course and poor prognosis of hypertrophic cardiomyopathy due to mutations in FHL1. Int J Cardiol. 2015;191:194‐197. 25965631 10.1016/j.ijcard.2015.04.260

[126] Olivotto I , Oreziak A , Barriales‐Villa R , et al. Mavacamten for treatment of symptomatic obstructive hypertrophic cardiomyopathy (EXPLORER‐HCM): a randomised, double‐blind, placebo‐controlled, phase 3 trial. Lancet. 2020;396(10253):759‐769. 32871100 10.1016/S0140-6736(20)31792-X

[127] Desai MY , Owens A , Geske JB , et al. Dose‐Blinded Myosin Inhibition in Patients With Obstructive Hypertrophic Cardiomyopathy Referred for Septal Reduction Therapy: Outcomes Through 32 Weeks. Circulation. 2023;147(11):850‐863. 36335531 10.1161/CIRCULATIONAHA.122.062534

[128] Tian Z , Li L , Li X , et al. Effect of Mavacamten on Chinese Patients With Symptomatic Obstructive Hypertrophic Cardiomyopathy: The EXPLORER‐CN Randomized Clinical Trial. JAMA Cardiol. 2023;8(10):957‐965. 37639259 10.1001/jamacardio.2023.3030 PMC10463173

[129] Coats CJ , Masri A , Nassif ME , et al. Dosing and Safety Profile of Aficamten in Symptomatic Obstructive Hypertrophic Cardiomyopathy: Results From SEQUOIA‐HCM. J Am Heart Assoc. 2024;13(15):e035993. 39056349 10.1161/JAHA.124.035993 PMC11964075

[130] Dimopoulos MA , Beksac M , Pour L , et al. Belantamab Mafodotin, Pomalidomide, and Dexamethasone in Multiple Myeloma. N Engl J Med. 2024;391(5):408‐421. 38828951 10.1056/NEJMoa2403407

[131] Lonial S , Lee HC , Badros A , et al. Belantamab mafodotin for relapsed or refractory multiple myeloma (DREAMM‐2): a two‐arm, randomised, open‐label, phase 2 study. Lancet Oncol. 2020;21(2):207‐221. 31859245 10.1016/S1470-2045(19)30788-0

[132] Dimopoulos MA , Hungria VTM , Radinoff A , et al. Efficacy and safety of single‐agent belantamab mafodotin versus pomalidomide plus low‐dose dexamethasone in patients with relapsed or refractory multiple myeloma (DREAMM‐3): a phase 3, open‐label, randomised study. Lancet Haematol. 2023;10(10):e801‐e812. 37793771 10.1016/S2352-3026(23)00243-0

[133] Mendell JR , Sahenk Z , Lehman K , et al. Assessment of Systemic Delivery of rAAVrh74.MHCK7.micro‐dystrophin in Children With Duchenne Muscular Dystrophy: A Nonrandomized Controlled Trial. JAMA Neurol. 2020;77(9):1122‐1131. 32539076 10.1001/jamaneurol.2020.1484 PMC7296461

[134] Mercuri E , Vilchez JJ , Boespflug‐Tanguy O , et al. Safety and efficacy of givinostat in boys with Duchenne muscular dystrophy (EPIDYS): a multicentre, randomised, double‐blind, placebo‐controlled, phase 3 trial. Lancet Neurol. 2024;23(4):393‐403. 38508835 10.1016/S1474-4422(24)00036-X

[135] McDonald CM , Shieh PB , Abdel‐Hamid HZ , et al. Open‐Label Evaluation of Eteplirsen in Patients with Duchenne Muscular Dystrophy Amenable to Exon 51 Skipping: PROMOVI Trial. J Neuromuscul Dis. 2021;8(6):989‐1001. 34120909 10.3233/JND-210643 PMC8673535

[136] Hilfiker‐Kleiner D , Haghikia A , Berliner D , et al. Bromocriptine for the treatment of peripartum cardiomyopathy: a multicentre randomized study. Eur Heart J. 2017;38(35):2671‐2679. 28934837 10.1093/eurheartj/ehx355 PMC5837241

[137] Benson MD , Waddington‐Cruz M , Berk JL , et al. Inotersen Treatment for Patients with Hereditary Transthyretin Amyloidosis. N Engl J Med. 2018;379(1):22‐31. 29972757 10.1056/NEJMoa1716793 PMC12611561

[138] Giusti, II , Rodrigues CG , Salles FB , et al. High doses of vascular endothelial growth factor 165 safely, but transiently, improve myocardial perfusion in no‐option ischemic disease. Hum Gene Ther Methods. 2013;24(5):298‐306. 23944648 10.1089/hgtb.2012.221

[139] Rosengart TK , Bishawi MM , Halbreiner MS , et al. Long‐term follow‐up assessment of a phase 1 trial of angiogenic gene therapy using direct intramyocardial administration of an adenoviral vector expressing the VEGF121 cDNA for the treatment of diffuse coronary artery disease. Hum Gene Ther. 2013;24(2):203‐208. 23137122 10.1089/hum.2012.137 PMC3581022

[140] Landstrom AP , Kellen CA , Dixit SS , et al. Junctophilin‐2 expression silencing causes cardiocyte hypertrophy and abnormal intracellular calcium‐handling. Circ Heart Fail. 2011;4(2):214‐223. 21216834 10.1161/CIRCHEARTFAILURE.110.958694 PMC3059380

[141] Friedrich FW , Bausero P , Sun Y , et al. A new polymorphism in human calmodulin III gene promoter is a potential modifier gene for familial hypertrophic cardiomyopathy. Eur Heart J. 2009;30(13):1648‐1655. 19429631 10.1093/eurheartj/ehp153

[142] Landstrom AP , Weisleder N , Batalden KB , et al. Mutations in JPH2‐encoded junctophilin‐2 associated with hypertrophic cardiomyopathy in humans. J Mol Cell Cardiol. 2007;42(6):1026‐1035. 17509612 10.1016/j.yjmcc.2007.04.006 PMC4318564

[143] Matsushita Y , Furukawa T , Kasanuki H , et al. Mutation of junctophilin type 2 associated with hypertrophic cardiomyopathy. J Hum Genet. 2007;52(6):543‐548. 17476457 10.1007/s10038-007-0149-y

[144] Chiu C , Tebo M , Ingles J , et al. Genetic screening of calcium regulation genes in familial hypertrophic cardiomyopathy. J Mol Cell Cardiol. 2007;43(3):337‐343. 17655857 10.1016/j.yjmcc.2007.06.009

[145] Roberts R . JPH2 Mutant Gene Causes Familial Hypertrophic Cardiomyopathy: A Possible Model to Unravel the Subtlety of Calcium‐Regulated Contractility. JACC Basic Transl Sci. 2017;2(1):68‐70. 30167555 10.1016/j.jacbts.2016.11.007 PMC6113546

[146] Vanninen SUM , Leivo K , Seppälä EH , et al. Heterozygous junctophilin‐2 (JPH2) p.(Thr161Lys) is a monogenic cause for HCM with heart failure. PLoS One. 2018;13(9):e0203422. 30235249 10.1371/journal.pone.0203422 PMC6147424

[147] Cheng Z , Fang T , Huang J , Guo Y , Alam M , Qian H . Hypertrophic Cardiomyopathy: From Phenotype and Pathogenesis to Treatment. Front Cardiovasc Med. 2021;8:722340. 34760939 10.3389/fcvm.2021.722340 PMC8572854

[148] Green EM , Wakimoto H , Anderson RL , et al. A small‐molecule inhibitor of sarcomere contractility suppresses hypertrophic cardiomyopathy in mice. Science. 2016;351(6273):617‐621. 26912705 10.1126/science.aad3456 PMC4784435

[149] Chuang C , Collibee S , Ashcraft L , et al. Discovery of Aficamten (CK‐274), a Next‐Generation Cardiac Myosin Inhibitor for the Treatment of Hypertrophic Cardiomyopathy. J Med Chem. 2021;64(19):14142‐14152. 34606259 10.1021/acs.jmedchem.1c01290

[150] Singh SR , Zech ATL , Geertz B , et al. Activation of Autophagy Ameliorates Cardiomyopathy in Mybpc3‐Targeted Knockin Mice. Circ Heart Fail. 2017;10(10):e004140. 29021349 10.1161/CIRCHEARTFAILURE.117.004140 PMC5679453

[151] Maron BJ , Desai MY , Nishimura RA , et al. Management of Hypertrophic Cardiomyopathy: JACC State‐of‐the‐Art Review. J Am Coll Cardiol. 2022;79(4):390‐414. 35086661 10.1016/j.jacc.2021.11.021

[152] Prondzynski M , Mearini G , Carrier L . Gene therapy strategies in the treatment of hypertrophic cardiomyopathy. Pflugers Arch. 2019;471(5):807‐815. 29971600 10.1007/s00424-018-2173-5

[153] Merkulov S , Chen X , Chandler MP , Stelzer JE . In vivo cardiac myosin binding protein C gene transfer rescues myofilament contractile dysfunction in cardiac myosin binding protein C null mice. Circ Heart Fail. 2012;5(5):635‐644. 22855556 10.1161/CIRCHEARTFAILURE.112.968941 PMC4860813

[154] Carrier L . Targeting the population for gene therapy with MYBPC3. J Mol Cell Cardiol. 2021;150:101‐108. 33049255 10.1016/j.yjmcc.2020.10.003

[155] Prondzynski M , Krämer E , Laufer SD , et al. Evaluation of MYBPC3 trans‐Splicing and Gene Replacement as Therapeutic Options in Human iPSC‐Derived Cardiomyocytes. Mol Ther Nucleic Acids. 2017;7:475‐486. 28624223 10.1016/j.omtn.2017.05.008 PMC5458066

[156] Behrens‐Gawlik V , Mearini G , Gedicke‐Hornung C , Richard P , Carrier L . MYBPC3 in hypertrophic cardiomyopathy: from mutation identification to RNA‐based correction. Pflugers Arch. 2014;466(2):215‐223. 24337823 10.1007/s00424-013-1409-7

[157] van Velzen HG , Schinkel AFL , Oldenburg RA , et al. Clinical Characteristics and Long‐Term Outcome of Hypertrophic Cardiomyopathy in Individuals With a MYBPC3 (Myosin‐Binding Protein C) Founder Mutation. Circ Cardiovasc Genet. 2017;10(4):e001660. 28794111 10.1161/CIRCGENETICS.116.001660

[158] Seeger T , Shrestha R , Lam CK , et al. A Premature Termination Codon Mutation in MYBPC3 Causes Hypertrophic Cardiomyopathy via Chronic Activation of Nonsense‐Mediated Decay. Circulation. 2019;139(6):799‐811. 30586709 10.1161/CIRCULATIONAHA.118.034624 PMC6443405

[159] Mearini G , Simpel D , Geertz B , et al. P236Evaluation of safety and feasibility of Mybpc3 gene therapy in a mouse model of hypertrophic cardiomyopathy. Cardiovascular Research. 2014;103(suppl_1):S42‐S42.

[160] Li J , Mamidi R , Doh CY , et al. AAV9 gene transfer of cMyBPC N‐terminal domains ameliorates cardiomyopathy in cMyBPC‐deficient mice. JCI Insight. 2020;5(17):e130182. 32750038 10.1172/jci.insight.130182 PMC7526450

[161] Anderson BR , Jensen ML , Hagedorn PH , et al. Allele‐Selective Knockdown of MYH7 Using Antisense Oligonucleotides. Mol Ther Nucleic Acids. 2020;19:1290‐1298. 32092825 10.1016/j.omtn.2020.01.012 PMC7033438

[162] Yue P , Xia S , Wu G , et al. Attenuation of Cardiomyocyte Hypertrophy via Depletion Myh7 using CASAAV. Cardiovasc Toxicol. 2021;21(3):255‐264. 33098074 10.1007/s12012-020-09617-y

[163] Bu H , Ding Y , Li J , et al. Inhibition of mTOR or MAPK ameliorates vmhcl/myh7 cardiomyopathy in zebrafish. JCI Insight. 2021;6(24):e154215. 34935644 10.1172/jci.insight.154215 PMC8783688

[164] Tyska MJ , Hayes E , Giewat M , Seidman CE , Seidman JG , Warshaw DM . Single‐molecule mechanics of R403Q cardiac myosin isolated from the mouse model of familial hypertrophic cardiomyopathy. Circ Res. 2000;86(7):737‐744. 10764406 10.1161/01.res.86.7.737

[165] Bell CL , Vandenberghe LH , Bell P , et al. The AAV9 receptor and its modification to improve in vivo lung gene transfer in mice. J Clin Invest. 2011;121(6):2427‐2435. 21576824 10.1172/JCI57367 PMC3104778

[166] Prasad KM , Xu Y , Yang Z , Acton ST , French BA . Robust cardiomyocyte‐specific gene expression following systemic injection of AAV: in vivo gene delivery follows a Poisson distribution. Gene Ther. 2011;18(1):43‐52. 20703310 10.1038/gt.2010.105 PMC2988989

[167] Ma S , Jiang W , Liu X , et al. Efficient Correction of a Hypertrophic Cardiomyopathy Mutation by ABEmax‐NG. Circ Res. 2021;129(10):895‐908. 34525843 10.1161/CIRCRESAHA.120.318674

[168] Han P , Li W , Lin CH , et al. A long noncoding RNA protects the heart from pathological hypertrophy. Nature. 2014;514(7520):102‐106. 25119045 10.1038/nature13596 PMC4184960

[169] Scherba JC , Halushka MK , Andersen ND , et al. BRG1 is a biomarker of hypertrophic cardiomyopathy in human heart specimens. Sci Rep. 2022;12(1):7996. 35581268 10.1038/s41598-022-11829-x PMC9114001

[170] Helms AS , Alvarado FJ , Yob J , et al. Genotype‐Dependent and ‐Independent Calcium Signaling Dysregulation in Human Hypertrophic Cardiomyopathy. Circulation. 2016;134(22):1738‐1748. 27688314 10.1161/CIRCULATIONAHA.115.020086 PMC5127749

[171] Peña JR , Szkudlarek AC , Warren CM , et al. Neonatal gene transfer of Serca2a delays onset of hypertrophic remodeling and improves function in familial hypertrophic cardiomyopathy. J Mol Cell Cardiol. 2010;49(6):993‐1002. 20854827 10.1016/j.yjmcc.2010.09.010 PMC2982190

[172] Gaffin RD , Peña JR , Alves MS , et al. Long‐term rescue of a familial hypertrophic cardiomyopathy caused by a mutation in the thin filament protein, tropomyosin, via modulation of a calcium cycling protein. J Mol Cell Cardiol. 2011;51(5):812‐820. 21840315 10.1016/j.yjmcc.2011.07.026 PMC3221410

[173] Jessup M , Greenberg B , Mancini D , et al. Calcium Upregulation by Percutaneous Administration of Gene Therapy in Cardiac Disease (CUPID): a phase 2 trial of intracoronary gene therapy of sarcoplasmic reticulum Ca2+‐ATPase in patients with advanced heart failure. Circulation. 2011;124(3):304‐313. 21709064 10.1161/CIRCULATIONAHA.111.022889 PMC5843948

[174] Kim M , Hunter RW , Garcia‐Menendez L , et al. Mutation in the γ2‐subunit of AMP‐activated protein kinase stimulates cardiomyocyte proliferation and hypertrophy independent of glycogen storage. Circ Res. 2014;114(6):966‐975. 24503893 10.1161/CIRCRESAHA.114.302364 PMC3971100

[175] Arad M , Moskowitz IP , Patel VV , et al. Transgenic mice overexpressing mutant PRKAG2 define the cause of Wolff‐Parkinson‐White syndrome in glycogen storage cardiomyopathy. Circulation. 2003;107(22):2850‐2856. 12782567 10.1161/01.CIR.0000075270.13497.2B

[176] Ben Jehuda R , Eisen B , Shemer Y , et al. CRISPR correction of the PRKAG2 gene mutation in the patient's induced pluripotent stem cell‐derived cardiomyocytes eliminates electrophysiological and structural abnormalities. Heart Rhythm. 2018;15(2):267‐276. 28917552 10.1016/j.hrthm.2017.09.024

[177] Zhan Y , Sun X , Li B , et al. Establishment of a PRKAG2 cardiac syndrome disease model and mechanism study using human induced pluripotent stem cells. J Mol Cell Cardiol. 2018;117:49‐61. 29452156 10.1016/j.yjmcc.2018.02.007

[178] Mosqueira D , Mannhardt I , Bhagwan JR , et al. CRISPR/Cas9 editing in human pluripotent stem cell‐cardiomyocytes highlights arrhythmias, hypocontractility, and energy depletion as potential therapeutic targets for hypertrophic cardiomyopathy. Eur Heart J. 2018;39(43):3879‐3892. 29741611 10.1093/eurheartj/ehy249 PMC6234851

[179] Elliott PM , Anastasakis A , Asimaki A , et al. Definition and treatment of arrhythmogenic cardiomyopathy: an updated expert panel report. Eur J Heart Fail. 2019;21(8):955‐964. 31210398 10.1002/ejhf.1534 PMC6685753

[180] James CA , Calkins H . Arrhythmogenic Right Ventricular Cardiomyopathy: Progress Toward Personalized Management. Annu Rev Med. 2019;70:1‐18. 30355260 10.1146/annurev-med-041217-010932

[181] Cruz FM , Sanz‐Rosa D , Roche‐Molina M , et al. Exercise triggers ARVC phenotype in mice expressing a disease‐causing mutated version of human plakophilin‐2. J Am Coll Cardiol. 2015;65(14):1438‐1450. 25857910 10.1016/j.jacc.2015.01.045

[182] Sen‐Chowdhry S , Syrris P , McKenna WJ . Role of genetic analysis in the management of patients with arrhythmogenic right ventricular dysplasia/cardiomyopathy. J Am Coll Cardiol. 2007;50(19):1813‐1821. 17980246 10.1016/j.jacc.2007.08.008

[183] Towbin JA . Inherited cardiomyopathies. Circ J. 2014;78(10):2347‐2356. 25186923 10.1253/circj.cj-14-0893 PMC4467885

[184] Watkins H , Ashrafian H , Redwood C . Inherited cardiomyopathies. N Engl J Med. 2011;364(17):1643‐1656. 21524215 10.1056/NEJMra0902923

[185] Brun F , Gigli M , Graw SL , et al. FLNC truncations cause arrhythmogenic right ventricular cardiomyopathy. J Med Genet. 2020;57(4):254‐257. 31924696 10.1136/jmedgenet-2019-106394 PMC7539291

[186] Elias Neto J , Tonet J , Frank R , Fontaine G . Arrhythmogenic Right Ventricular Cardiomyopathy/Dysplasia (ARVC/D) ‐ What We Have Learned after 40 Years of the Diagnosis of This Clinical Entity. Arq Bras Cardiol. 2019;112(1):91‐103. 30673021 10.5935/abc.20180266 PMC6317628

[187] Mundisugih J , Ravindran D , Kizana E . Exploring the Therapeutic Potential of Gene Therapy in Arrhythmogenic Right Ventricular Cardiomyopathy. Biomedicines. 2024;12(6):1351. 38927558 10.3390/biomedicines12061351 PMC11201581

[188] Corrado D , Wichter T , Link MS , et al. Treatment of arrhythmogenic right ventricular cardiomyopathy/dysplasia: an international task force consensus statement. Eur Heart J. 2015;36(46):3227‐3237. 26216920 10.1093/eurheartj/ehv162 PMC4670964

[189] Garcia FC , Bazan V , Zado ES , Ren JF , Marchlinski FE . Epicardial substrate and outcome with epicardial ablation of ventricular tachycardia in arrhythmogenic right ventricular cardiomyopathy/dysplasia. Circulation. 2009;120(5):366‐375. 19620503 10.1161/CIRCULATIONAHA.108.834903

[190] Yoda M , Minami K , Fritzsche D , Tendrich G , Schulte‐Eistrup S , Koerfer R . Three cases of orthotopic heart transplantation for arrhythmogenic right ventricular cardiomyopathy. Ann Thorac Surg. 2005;80(6):2358‐2360. 16305911 10.1016/j.athoracsur.2004.07.071

[191] Cerrone M , Montnach J , Lin X , et al. Plakophilin‐2 is required for transcription of genes that control calcium cycling and cardiac rhythm. Nat Commun. 2017;8(1):106. 28740174 10.1038/s41467-017-00127-0 PMC5524637

[192] Wu I , Zeng A , Greer‐Short A , et al. AAV9:PKP2 improves heart function and survival in a Pkp2‐deficient mouse model of arrhythmogenic right ventricular cardiomyopathy. Commun Med (Lond). 2024;4(1):38. 38499690 10.1038/s43856-024-00450-w PMC10948840

[193] van Opbergen CJM , Narayanan B , Sacramento CB , et al. AAV‐Mediated Delivery of Plakophilin‐2a Arrests Progression of Arrhythmogenic Right Ventricular Cardiomyopathy in Murine Hearts: Preclinical Evidence Supporting Gene Therapy in Humans. Circ Genom Precis Med. 2024;17(1):e004305. 38288614 10.1161/CIRCGEN.123.004305 PMC10923105

[194] Feyen DAM , Perea‐Gil I , Maas RGC , et al. Unfolded Protein Response as a Compensatory Mechanism and Potential Therapeutic Target in PLN R14del Cardiomyopathy. Circulation. 2021;144(5):382‐392. 33928785 10.1161/CIRCULATIONAHA.120.049844 PMC8667423

[195] Karakikes I , Stillitano F , Nonnenmacher M , et al. Correction of human phospholamban R14del mutation associated with cardiomyopathy using targeted nucleases and combination therapy. Nat Commun. 2015;6:6955. 25923014 10.1038/ncomms7955 PMC4421839

[196] Dave J , Raad N , Mittal N , et al. Gene editing reverses arrhythmia susceptibility in humanized PLN‐R14del mice: modelling a European cardiomyopathy with global impact. Cardiovasc Res. 2022;118(15):3140‐3150. 35191471 10.1093/cvr/cvac021 PMC9732517

[197] Zankov D , Ohno S . Desmoglein 2 mutant mice reproduce arrhythmogenic right ventricular cardiomyopathy patients' phenotype. European Heart Journal. 2022;43(Supplement_2).

[198] Sonoda K , Nagase S , Aiba T , et al. Homozygous or compound heterozygous variants in DSG2 are mainly causative of Japanese arrhythmogenic right ventricular cardiomyopathy. European Heart Journal. 2023;44(Supplement_2)

[199] Shiba M , Higo S , Kondo T , et al. Phenotypic recapitulation and correction of desmoglein‐2‐deficient cardiomyopathy using human‐induced pluripotent stem cell‐derived cardiomyocytes. Hum Mol Genet. 2021;30(15):1384‐1397. 33949662 10.1093/hmg/ddab127 PMC8283207

[200] McNally EM , Mestroni L . Dilated Cardiomyopathy: Genetic Determinants and Mechanisms. Circ Res. 2017;121(7):731‐748. 28912180 10.1161/CIRCRESAHA.116.309396 PMC5626020

[201] Hershberger RE , Hedges DJ , Morales A . Dilated cardiomyopathy: the complexity of a diverse genetic architecture. Nat Rev Cardiol. 2013;10(9):531‐547. 23900355 10.1038/nrcardio.2013.105

[202] Cho KW , Lee J , Kim Y . Genetic Variations Leading to Familial Dilated Cardiomyopathy. Mol Cells. 2016;39(10):722‐727. 27802374 10.14348/molcells.2016.0061 PMC5104879

[203] Hänselmann A , Veltmann C , Bauersachs J , Berliner D . Dilated cardiomyopathies and non‐compaction cardiomyopathy. Herz. 2020;45(3):212‐220. 32107565 10.1007/s00059-020-04903-5 PMC7198644

[204] Mazzarotto F , Tayal U , Buchan RJ , et al. Reevaluating the Genetic Contribution of Monogenic Dilated Cardiomyopathy. Circulation. 2020;141(5):387‐398. 31983221 10.1161/CIRCULATIONAHA.119.037661 PMC7004454

[205] Jordan E , Peterson L , Ai T , et al. Evidence‐Based Assessment of Genes in Dilated Cardiomyopathy. Circulation. 2021;144(1):7‐19. 33947203 10.1161/CIRCULATIONAHA.120.053033 PMC8247549

[206] Eijgenraam TR , Boogerd CJ , Stege NM , et al. Protein Aggregation Is an Early Manifestation of Phospholamban p.(Arg14del)‐Related Cardiomyopathy: Development of PLN‐R14del‐Related Cardiomyopathy. Circ Heart Fail. 2021;14(11):e008532. 34587756 10.1161/CIRCHEARTFAILURE.121.008532 PMC8589082

[207] Yost O , Friedenberg SG , Jesty SA , Olby NJ , Meurs KM . The R9H phospholamban mutation is associated with highly penetrant dilated cardiomyopathy and sudden death in a spontaneous canine model. Gene. 2019;697:118‐122. 30794913 10.1016/j.gene.2019.02.022

[208] Ceholski DK , Turnbull IC , Kong CW , et al. Functional and transcriptomic insights into pathogenesis of R9C phospholamban mutation using human induced pluripotent stem cell‐derived cardiomyocytes. J Mol Cell Cardiol. 2018;119:147‐154. 29752948 10.1016/j.yjmcc.2018.05.007 PMC6039110

[209] Gerull B , Gramlich M , Atherton J , et al. Mutations of TTN, encoding the giant muscle filament titin, cause familial dilated cardiomyopathy. Nat Genet. 2002;30(2):201‐204. 11788824 10.1038/ng815

[210] Yoskovitz G , Peled Y , Gramlich M , et al. A novel titin mutation in adult‐onset familial dilated cardiomyopathy. Am J Cardiol. 2012;109(11):1644‐1650. 22475360 10.1016/j.amjcard.2012.01.392

[211] Hinson JT , Chopra A , Nafissi N , et al. HEART DISEASE. Titin mutations in iPS cells define sarcomere insufficiency as a cause of dilated cardiomyopathy. Science. 2015;349(6251):982‐986. 26315439 10.1126/science.aaa5458 PMC4618316

[212] Vikhorev PG , Vikhoreva NN , Yeung W , et al. Titin‐truncating mutations associated with dilated cardiomyopathy alter length‐dependent activation and its modulation via phosphorylation. Cardiovasc Res. 2022;118(1):241‐253. 33135063 10.1093/cvr/cvaa316 PMC8752363

[213] Akhtar MM , Lorenzini M , Cicerchia M , et al. Clinical Phenotypes and Prognosis of Dilated Cardiomyopathy Caused by Truncating Variants in the TTN Gene. Circ Heart Fail. 2020;13(10):e006832. 32964742 10.1161/CIRCHEARTFAILURE.119.006832

[214] Ang YS , Rivas RN , Ribeiro AJS , et al. Disease Model of GATA4 Mutation Reveals Transcription Factor Cooperativity in Human Cardiogenesis. Cell. 2016;167(7):1734‐1749. e22. 27984724 10.1016/j.cell.2016.11.033 PMC5180611

[215] Li RG , Li L , Qiu XB , et al. GATA4 loss‐of‐function mutation underlies familial dilated cardiomyopathy. Biochem Biophys Res Commun. 2013;439(4):591‐596. 24041700 10.1016/j.bbrc.2013.09.023

[216] Zhao L , Xu JH , Xu WJ , et al. A novel GATA4 loss‐of‐function mutation responsible for familial dilated cardiomyopathy. Int J Mol Med. 2014;33(3):654‐660. 24366163 10.3892/ijmm.2013.1600

[217] Li J , Liu WD , Yang ZL , et al. Prevalence and spectrum of GATA4 mutations associated with sporadic dilated cardiomyopathy. Gene. 2014;548(2):174‐181. 25017055 10.1016/j.gene.2014.07.022

[218] Khan RS , Pahl E , Dellefave‐Castillo L , et al. Genotype and Cardiac Outcomes in Pediatric Dilated Cardiomyopathy. J Am Heart Assoc. 2022;11(1):e022854. 34935411 10.1161/JAHA.121.022854 PMC9075202

[219] Rani DS , Vijaya Kumar A , Nallari P , et al. Novel Mutations in β‐MYH7 Gene in Indian Patients With Dilated Cardiomyopathy. CJC Open. 2022;4(1):1‐11. 35072022 10.1016/j.cjco.2021.07.020 PMC8767027

[220] Arimura T , Onoue K , Takahashi‐Tanaka Y , et al. Nuclear accumulation of androgen receptor in gender difference of dilated cardiomyopathy due to lamin A/C mutations. Cardiovasc Res. 2013;99(3):382‐394. 23631840 10.1093/cvr/cvt106

[221] Cai ZJ , Lee YK , Lau YM , et al. Expression of Lmna‐R225X nonsense mutation results in dilated cardiomyopathy and conduction disorders (DCM‐CD) in mice: Impact of exercise training. Int J Cardiol. 2020;298:85‐92. 31668660 10.1016/j.ijcard.2019.09.058

[222] Sabater‐Molina M , Navarro M , García‐Molina Sáez E , et al. Mutation in JPH2 cause dilated cardiomyopathy. Clin Genet. 2016;90(5):468‐469. 27471098 10.1111/cge.12825

[223] Jones EG , Mazaheri N , Maroofian R , et al. Analysis of enriched rare variants in JPH2‐encoded junctophilin‐2 among Greater Middle Eastern individuals reveals a novel homozygous variant associated with neonatal dilated cardiomyopathy. Sci Rep. 2019;9(1):9038. 31227780 10.1038/s41598-019-44987-6 PMC6588559

[224] Mann SA , Castro ML , Ohanian M , et al. R222Q SCN5A mutation is associated with reversible ventricular ectopy and dilated cardiomyopathy. J Am Coll Cardiol. 2012;60(16):1566‐1573. 22999724 10.1016/j.jacc.2012.05.050

[225] Ding Y , Dvornikov AV , Ma X , et al. Haploinsufficiency of mechanistic target of rapamycin ameliorates bag3 cardiomyopathy in adult zebrafish. Dis Model Mech. 2019;12(10):dmm040154. 31492659 10.1242/dmm.040154 PMC6826022

[226] Domínguez F , Cuenca S , Bilińska Z , et al. Dilated Cardiomyopathy Due to BLC2‐Associated Athanogene 3 (BAG3) Mutations. J Am Coll Cardiol. 2018;72(20):2471‐2481. 30442290 10.1016/j.jacc.2018.08.2181 PMC6688826

[227] Arimura T , Ishikawa T , Nunoda S , Kawai S , Kimura A . Dilated cardiomyopathy‐associated BAG3 mutations impair Z‐disc assembly and enhance sensitivity to apoptosis in cardiomyocytes. Hum Mutat. 2011;32(12):1481‐1491. 21898660 10.1002/humu.21603

[228] Hakui H , Kioka H , Miyashita Y , et al. Loss‐of‐function mutations in the co‐chaperone protein BAG5 cause dilated cardiomyopathy requiring heart transplantation. Sci Transl Med. 2022;14(628):eabf3274. 35044787 10.1126/scitranslmed.abf3274

[229] Weintraub RG , Semsarian C , Macdonald P . Dilated cardiomyopathy. Lancet. 2017;390(10092):400‐414. 28190577 10.1016/S0140-6736(16)31713-5

[230] Rupp S , Jux C . Advances in heart failure therapy in pediatric patients with dilated cardiomyopathy. Heart Fail Rev. 2018;23(4):555‐562. 29564593 10.1007/s10741-018-9692-1

[231] Verdonschot JAJ , Hazebroek MR , Ware JS , Prasad SK , Heymans SRB . Role of Targeted Therapy in Dilated Cardiomyopathy: The Challenging Road Toward a Personalized Approach. J Am Heart Assoc. 2019;8(11):e012514. 31433726 10.1161/JAHA.119.012514 PMC6585365

[232] Zhan DY , Morimoto S , Du CK , et al. Therapeutic effect of {beta}‐adrenoceptor blockers using a mouse model of dilated cardiomyopathy with a troponin mutation. Cardiovasc Res. 2009;84(1):64‐71. 19477965 10.1093/cvr/cvp168

[233] Li B , Guo Y , Zhan Y , et al. Cardiac Overexpression of XIN Prevents Dilated Cardiomyopathy Caused by TNNT2 ΔK210 Mutation. Front Cell Dev Biol. 2021;9:691749. 34222259 10.3389/fcell.2021.691749 PMC8247596

[234] Migliore L , Galvagni F , Pierantozzi E , Sorrentino V , Rossi D . Allele‐specific silencing by RNAi of R92Q and R173W mutations in cardiac troponin T. Exp Biol Med (Maywood). 2022;247(10):805‐814. 35067102 10.1177/15353702211072453 PMC9160939

[235] Lee JM , Nobumori C , Tu Y , et al. Modulation of LMNA splicing as a strategy to treat prelamin A diseases. J Clin Invest. 2016;126(4):1592‐1602. 26999604 10.1172/JCI85908 PMC4811112

[236] Santiago‐Fernández O , Osorio FG , Quesada V , et al. Development of a CRISPR/Cas9‐based therapy for Hutchinson‐Gilford progeria syndrome. Nat Med. 2019;25(3):423‐426. 30778239 10.1038/s41591-018-0338-6 PMC6546610

[237] Beyret E , Liao HK , Yamamoto M , et al. Single‐dose CRISPR‐Cas9 therapy extends lifespan of mice with Hutchinson‐Gilford progeria syndrome. Nat Med. 2019;25(3):419‐422. 30778240 10.1038/s41591-019-0343-4 PMC6541418

[238] Lee YK , Lau YM , Cai ZJ , et al. Modeling Treatment Response for Lamin A/C Related Dilated Cardiomyopathy in Human Induced Pluripotent Stem Cells. J Am Heart Assoc. 2017;6(8):e005677. 28754655 10.1161/JAHA.117.005677 PMC5586427

[239] Herman DS , Lam L , Taylor MR , et al. Truncations of titin causing dilated cardiomyopathy. N Engl J Med. 2012;366(7):619‐628. 22335739 10.1056/NEJMoa1110186 PMC3660031

[240] Granzier HL , Labeit S . The giant protein titin: a major player in myocardial mechanics, signaling, and disease. Circ Res. 2004;94(3):284‐295. 14976139 10.1161/01.RES.0000117769.88862.F8

[241] Musa H , Meek S , Gautel M , Peddie D , Smith AJ , Peckham M . Targeted homozygous deletion of M‐band titin in cardiomyocytes prevents sarcomere formation. J Cell Sci. 2006;119(Pt 20):4322‐4331. 17038546 10.1242/jcs.03198

[242] Norton N , Li D , Rampersaud E , et al. Exome sequencing and genome‐wide linkage analysis in 17 families illustrate the complex contribution of TTN truncating variants to dilated cardiomyopathy. Circ Cardiovasc Genet. 2013;6(2):144‐153. 23418287 10.1161/CIRCGENETICS.111.000062 PMC3815606

[243] Davis J , Davis LC , Correll RN , et al. A Tension‐Based Model Distinguishes Hypertrophic versus Dilated Cardiomyopathy. Cell. 2016;165(5):1147‐1159. 27114035 10.1016/j.cell.2016.04.002 PMC4874838

[244] Romano R , Ghahremani S , Zimmerman T , et al. Reading Frame Repair of TTN Truncation Variants Restores Titin Quantity and Functions. Circulation. 2022;145(3):194‐205. 34905694 10.1161/CIRCULATIONAHA.120.049997 PMC8766920

[245] Kaneko M , Hashikami K , Yamamoto S , Matsumoto H , Nishimoto T . Phospholamban Ablation Using CRISPR/Cas9 System Improves Mortality in a Murine Heart Failure Model. PLoS One. 2016;11(12):e0168486. 27992596 10.1371/journal.pone.0168486 PMC5161475

[246] Stillitano F , Turnbull IC , Karakikes I , et al. Genomic correction of familial cardiomyopathy in human engineered cardiac tissues. Eur Heart J. 2016;37(43):3282‐3284. 27450564 10.1093/eurheartj/ehw307 PMC6425468

[247] Hoshijima M , Ikeda Y , Iwanaga Y , et al. Chronic suppression of heart‐failure progression by a pseudophosphorylated mutant of phospholamban via in vivo cardiac rAAV gene delivery. Nat Med. 2002;8(8):864‐871. 12134142 10.1038/nm739

[248] Grote Beverborg N , Später D , Knöll R , et al. Phospholamban antisense oligonucleotides improve cardiac function in murine cardiomyopathy. Nat Commun. 2021;12(1):5180. 34462437 10.1038/s41467-021-25439-0 PMC8405807

[249] Maddatu TP , Garvey SM , Schroeder DG , et al. Dilated cardiomyopathy in the nmd mouse: transgenic rescue and QTLs that improve cardiac function and survival. Hum Mol Genet. 2005;14(21):3179‐3189. 16174646 10.1093/hmg/ddi349 PMC1350304

[250] Maddatu TP , Garvey SM , Schroeder DG , Hampton TG , Cox GA . Transgenic rescue of neurogenic atrophy in the nmd mouse reveals a role for Ighmbp2 in dilated cardiomyopathy. Hum Mol Genet. 2004;13(11):1105‐1115. 15069027 10.1093/hmg/ddh129 PMC1350377

[251] Hikoso S , Ikeda Y , Yamaguchi O , et al. Progression of heart failure was suppressed by inhibition of apoptosis signal‐regulating kinase 1 via transcoronary gene transfer. J Am Coll Cardiol. 2007;50(5):453‐462. 17662399 10.1016/j.jacc.2007.03.053

[252] Zentilin L , Puligadda U , Lionetti V , et al. Cardiomyocyte VEGFR‐1 activation by VEGF‐B induces compensatory hypertrophy and preserves cardiac function after myocardial infarction. Faseb j. 2010;24(5):1467‐1478. 20019242 10.1096/fj.09-143180

[253] Pepe M , Mamdani M , Zentilin L , et al. Intramyocardial VEGF‐B167 gene delivery delays the progression towards congestive failure in dogs with pacing‐induced dilated cardiomyopathy. Circ Res. 2010;106(12):1893‐1903. 20431055 10.1161/CIRCRESAHA.110.220855 PMC4879815

[254] Bry M , Kivelä R , Leppänen VM , Alitalo K . Vascular endothelial growth factor‐B in physiology and disease. Physiol Rev. 2014;94(3):779‐794. 24987005 10.1152/physrev.00028.2013

[255] Farzaneh Behelgardi M, Zahri S , Mashayekhi F , Mansouri K , Asghari SM . A peptide mimicking the binding sites of VEGF‐A and VEGF‐B inhibits VEGFR‐1/‐2 driven angiogenesis, tumor growth and metastasis. Sci Rep. 2018;8(1):17924. 30560942 10.1038/s41598-018-36394-0 PMC6298961

[256] Takemura G , Kanoh M , Minatoguchi S , Fujiwara H . Cardiomyocyte apoptosis in the failing heart–a critical review from definition and classification of cell death. Int J Cardiol. 2013;167(6):2373‐2386. 23498286 10.1016/j.ijcard.2013.01.163

[257] Nishida K , Yamaguchi O , Otsu K . Crosstalk between autophagy and apoptosis in heart disease. Circ Res. 2008;103(4):343‐351. 18703786 10.1161/CIRCRESAHA.108.175448

[258] Woitek F , Zentilin L , Hoffman NE , et al. Intracoronary Cytoprotective Gene Therapy: A Study of VEGF‐B167 in a Pre‐Clinical Animal Model of Dilated Cardiomyopathy. J Am Coll Cardiol. 2015;66(2):139‐153. 26160630 10.1016/j.jacc.2015.04.071 PMC4499859

[259] Kyrychenko S , Kyrychenko V , Badr MA , Ikeda Y , Sadoshima J , Shirokova N . Pivotal role of miR‐448 in the development of ROS‐induced cardiomyopathy. Cardiovasc Res. 2015;108(3):324‐334. 26503985 10.1093/cvr/cvv238 PMC4648202

[260] Tharp CA , Haywood ME , Sbaizero O , Taylor MRG , Mestroni L . The Giant Protein Titin's Role in Cardiomyopathy: Genetic, Transcriptional, and Post‐translational Modifications of TTN and Their Contribution to Cardiac Disease. Front Physiol. 2019;10:1436. 31849696 10.3389/fphys.2019.01436 PMC6892752

[261] Quattrocelli M , Crippa S , Montecchiani C , et al. Long‐term miR‐669a therapy alleviates chronic dilated cardiomyopathy in dystrophic mice. J Am Heart Assoc. 2013;2(4):e000284. 23963759 10.1161/JAHA.113.000284 PMC3828786

[262] Pankuweit S . Lamin A/C mutations in patients with dilated cardiomyopathy. Eur Heart J. 2018;39(10):861‐863. 29165585 10.1093/eurheartj/ehx650

[263] Jansweijer JA , Nieuwhof K , Russo F , et al. Truncating titin mutations are associated with a mild and treatable form of dilated cardiomyopathy. Eur J Heart Fail. 2017;19(4):512‐521. 27813223 10.1002/ejhf.673

[264] Sébillon P , Bouchier C , Bidot LD , et al. Expanding the phenotype of LMNA mutations in dilated cardiomyopathy and functional consequences of these mutations. J Med Genet. 2003;40(8):560‐567. 12920062 10.1136/jmg.40.8.560 PMC1735561

[265] Hasselberg NE , Haland TF , Saberniak J , et al. Lamin A/C cardiomyopathy: young onset, high penetrance, and frequent need for heart transplantation. Eur Heart J. 2018;39(10):853‐860. 29095976 10.1093/eurheartj/ehx596 PMC5939624

[266] Li D , Morales A , Gonzalez‐Quintana J , et al. Identification of novel mutations in RBM20 in patients with dilated cardiomyopathy. Clin Transl Sci. 2010;3(3):90‐97. 20590677 10.1111/j.1752-8062.2010.00198.x PMC2898174

[267] Refaat MM , Lubitz SA , Makino S , et al. Genetic variation in the alternative splicing regulator RBM20 is associated with dilated cardiomyopathy. Heart Rhythm. 2012;9(3):390‐396. 22004663 10.1016/j.hrthm.2011.10.016 PMC3516872

[268] Yadav S , Sitbon YH , Kazmierczak K , Szczesna‐Cordary D . Hereditary heart disease: pathophysiology, clinical presentation, and animal models of HCM, RCM, and DCM associated with mutations in cardiac myosin light chains. Pflugers Arch. 2019;471(5):683‐699. 30706179 10.1007/s00424-019-02257-4 PMC6476665

[269] Ditaranto R , Caponetti AG , Ferrara V , et al. Pediatric Restrictive Cardiomyopathies. Front Pediatr. 2021;9:745365. 35145940 10.3389/fped.2021.745365 PMC8822222

[270] Kostareva A , Kiselev A , Gudkova A , et al. Genetic Spectrum of Idiopathic Restrictive Cardiomyopathy Uncovered by Next‐Generation Sequencing. PLoS One. 2016;11(9):e0163362. 27662471 10.1371/journal.pone.0163362 PMC5035084

[271] Muchtar E , Blauwet LA , Gertz MA . Restrictive Cardiomyopathy: Genetics, Pathogenesis, Clinical Manifestations, Diagnosis, and Therapy. Circ Res. 2017;121(7):819‐837. 28912185 10.1161/CIRCRESAHA.117.310982

[272] Gallego‐Delgado M , Delgado JF , Brossa‐Loidi V , et al. Idiopathic Restrictive Cardiomyopathy Is Primarily a Genetic Disease. J Am Coll Cardiol. 2016;67(25):3021‐3023. 27339502 10.1016/j.jacc.2016.04.024

[273] Brodehl A , Ferrier RA , Hamilton SJ , et al. Mutations in FLNC are Associated with Familial Restrictive Cardiomyopathy. Hum Mutat. 2016;37(3):269‐279. 26666891 10.1002/humu.22942

[274] Kaski JP , Syrris P , Burch M , et al. Idiopathic restrictive cardiomyopathy in children is caused by mutations in cardiac sarcomere protein genes. Heart. 2008;94(11):1478‐1484. 18467357 10.1136/hrt.2007.134684

[275] Gambarin FI , Tagliani M , Arbustini E . Pure restrictive cardiomyopathy associated with cardiac troponin I gene mutation: mismatch between the lack of hypertrophy and the presence of disarray. Heart. 2008;94(10):1257. 18801787 10.1136/hrt.2008.154203

[276] Chen Y , Yang S , Li J , et al. Pediatric restrictive cardiomyopathy due to a heterozygous mutation of the TNNI3 gene. J Biomed Res. 2014;28(1):59‐63. 24474965 10.7555/JBR.28.20120105 PMC3904176

[277] Parvatiyar MS , Pinto JR , Dweck D , Potter JD . Cardiac troponin mutations and restrictive cardiomyopathy. J Biomed Biotechnol. 2010;2010:350706. 20617149 10.1155/2010/350706 PMC2896668

[278] Mogensen J , Kubo T , Duque M , et al. Idiopathic restrictive cardiomyopathy is part of the clinical expression of cardiac troponin I mutations. J Clin Invest. 2003;111(2):209‐216. 12531876 10.1172/JCI16336 PMC151864

[279] Kostareva A , Gudkova A , Sjöberg G , et al. Deletion in TNNI3 gene is associated with restrictive cardiomyopathy. Int J Cardiol. 2009;131(3):410‐412. 18006163 10.1016/j.ijcard.2007.07.108

[280] Menon SC , Michels VV , Pellikka PA , et al. Cardiac troponin T mutation in familial cardiomyopathy with variable remodeling and restrictive physiology. Clin Genet. 2008;74(5):445‐454. 18651846 10.1111/j.1399-0004.2008.01062.x PMC2575134

[281] Peddy SB , Vricella LA , Crosson JE , et al. Infantile restrictive cardiomyopathy resulting from a mutation in the cardiac troponin T gene. Pediatrics. 2006;117(5):1830‐1833. 16651346 10.1542/peds.2005-2301

[282] Rai TS , Ahmad S , Ahluwalia TS , et al. Genetic and clinical profile of Indian patients of idiopathic restrictive cardiomyopathy with and without hypertrophy. Mol Cell Biochem. 2009;331(1‐2):187‐192. 19449150 10.1007/s11010-009-0157-7

[283] Peled Y , Gramlich M , Yoskovitz G , et al. Titin mutation in familial restrictive cardiomyopathy. Int J Cardiol. 2014;171(1):24‐30. 24315344 10.1016/j.ijcard.2013.11.037

[284] Ploski R , Rydzanicz M , Ksiazczyk TM , et al. Evidence for troponin C (TNNC1) as a gene for autosomal recessive restrictive cardiomyopathy with fatal outcome in infancy. Am J Med Genet A. 2016;170(12):3241‐3248. 27604170 10.1002/ajmg.a.37860

[285] Zaleta‐Rivera K , Dainis A , Ribeiro AJS , et al. Allele‐Specific Silencing Ameliorates Restrictive Cardiomyopathy Attributable to a Human Myosin Regulatory Light Chain Mutation. Circulation. 2019;140(9):765‐778. 31315475 10.1161/CIRCULATIONAHA.118.036965 PMC13307499

[286] Towbin JA , Lorts A , Jefferies JL . Left ventricular non‐compaction cardiomyopathy. Lancet. 2015;386(9995):813‐825. 25865865 10.1016/S0140-6736(14)61282-4

[287] Dong X , Fan P , Tian T , et al. Recent advancements in the molecular genetics of left ventricular noncompaction cardiomyopathy. Clin Chim Acta. 2017;465:40‐44. 27989498 10.1016/j.cca.2016.12.013

[288] Lin Y , Huang J , Zhu Z , et al. Overlap phenotypes of the left ventricular noncompaction and hypertrophic cardiomyopathy with complex arrhythmias and heart failure induced by the novel truncated DSC2 mutation. Orphanet J Rare Dis. 2021;16(1):496. 34819141 10.1186/s13023-021-02112-9 PMC8611834

[289] Milano A , Vermeer AM , Lodder EM , et al. HCN4 mutations in multiple families with bradycardia and left ventricular noncompaction cardiomyopathy. J Am Coll Cardiol. 2014;64(8):745‐756. 25145517 10.1016/j.jacc.2014.05.045

[290] Hirono K , Hata Y , Miyao N , et al. Increased Burden of Ion Channel Gene Variants Is Related to Distinct Phenotypes in Pediatric Patients With Left Ventricular Noncompaction. Circ Genom Precis Med. 2020;13(4):e002940. 32600061 10.1161/CIRCGEN.119.002940

[291] Luxán G , Casanova JC , Martínez‐Poveda B , et al. Mutations in the NOTCH pathway regulator MIB1 cause left ventricular noncompaction cardiomyopathy. Nat Med. 2013;19(2):193‐201. 23314057 10.1038/nm.3046

[292] Kulikova O , Brodehl A , Kiseleva A , et al. The Desmin (DES) Mutation p.A337P Is Associated with Left‐Ventricular Non‐Compaction Cardiomyopathy. Genes (Basel). 2021;12(1):121. 33478057 10.3390/genes12010121 PMC7835827

[293] Zhang J , Han X , Lu Q , Feng Y , Ma A , Wang T . Left ventricular non‐compaction cardiomyopathy associated with the PRKAG2 mutation. BMC Med Genomics. 2022;15(1):214. 36221081 10.1186/s12920-022-01361-2 PMC9552423

[294] Kolokotronis K , Kühnisch J , Klopocki E , et al. Biallelic mutation in MYH7 and MYBPC3 leads to severe cardiomyopathy with left ventricular noncompaction phenotype. Hum Mutat. 2019;40(8):1101‐1114. 30924982 10.1002/humu.23757

[295] Kodo K , Ong SG , Jahanbani F , et al. iPSC‐derived cardiomyocytes reveal abnormal TGF‐β signalling in left ventricular non‐compaction cardiomyopathy. Nat Cell Biol. 2016;18(10):1031‐1042. 27642787 10.1038/ncb3411 PMC5042877

[296] Li D , Wang C . Advances in symptomatic therapy for left ventricular non‐compaction in children. Front Pediatr. 2023;11:1147362. 37215603 10.3389/fped.2023.1147362 PMC10192632

[297] Stacey RB , Caine AJ, Jr. , Hundley WG . Evaluation and management of left ventricular noncompaction cardiomyopathy. Curr Heart Fail Rep. 2015;12(1):61‐67. 25399629 10.1007/s11897-014-0237-1

[298] Arad M , Maron BJ , Gorham JM , et al. Glycogen storage diseases presenting as hypertrophic cardiomyopathy. N Engl J Med. 2005;352(4):362‐372. 15673802 10.1056/NEJMoa033349

[299] Hedberg‐Oldfors C , Oldfors A . Polyglucosan storage myopathies. Mol Aspects Med. 2015;46:85‐100. 26278982 10.1016/j.mam.2015.08.006

[300] Eduardo BS . Too much sugar leaves a sour taste: A cardiac disease caused by excess glycogen deposit. EBioMedicine. 2020;55:102764. 32339940 10.1016/j.ebiom.2020.102764 PMC7186573

[301] Yavari A , Sarma D , Sternick EB . Human γ2‐AMPK Mutations. Methods Mol Biol. 2018;1732:581‐619. 29480501 10.1007/978-1-4939-7598-3_37

[302] Arad M , Benson DW , Perez‐Atayde AR , et al. Constitutively active AMP kinase mutations cause glycogen storage disease mimicking hypertrophic cardiomyopathy. J Clin Invest. 2002;109(3):357‐362. 11827995 10.1172/JCI14571 PMC150860

[303] Laforêt P , Richard P , Said MA , et al. A new mutation in PRKAG2 gene causing hypertrophic cardiomyopathy with conduction system disease and muscular glycogenosis. Neuromuscul Disord. 2006;16(3):178‐182. 16487706 10.1016/j.nmd.2005.12.004

[304] Lopez‐Sainz A , Dominguez F , Lopes LR , et al. Clinical Features and Natural History of PRKAG2 Variant Cardiac Glycogenosis. J Am Coll Cardiol. 2020;76(2):186‐197. 32646569 10.1016/j.jacc.2020.05.029

[305] Thevenon J , Laurent G , Ader F , et al. High prevalence of arrhythmic and myocardial complications in patients with cardiac glycogenosis due to PRKAG2 mutations. Europace. 2017;19(4):651‐659. 28431061 10.1093/europace/euw067

[306] Xie C , Zhang YP , Song L , et al. Genome editing with CRISPR/Cas9 in postnatal mice corrects PRKAG2 cardiac syndrome. Cell Res. 2016;26(10):1099‐1111. 27573176 10.1038/cr.2016.101 PMC5113300

[307] Alcalai R , Arad M , Wakimoto H , et al. LAMP2 Cardiomyopathy: Consequences of Impaired Autophagy in the Heart. J Am Heart Assoc. 2021;10(17):e018829. 34459252 10.1161/JAHA.120.018829 PMC8649277

[308] Eskelinen EL . Roles of LAMP‐1 and LAMP‐2 in lysosome biogenesis and autophagy. Mol Aspects Med. 2006;27(5‐6):495‐502. 16973206 10.1016/j.mam.2006.08.005

[309] Stypmann J , Janssen PM , Prestle J , et al. LAMP‐2 deficient mice show depressed cardiac contractile function without significant changes in calcium handling. Basic Res Cardiol. 2006;101(4):281‐291. 16604439 10.1007/s00395-006-0591-6

[310] Dvornikov AV , Wang M , Yang J , et al. Phenotyping an adult zebrafish lamp2 cardiomyopathy model identifies mTOR inhibition as a candidate therapy. J Mol Cell Cardiol. 2019;133:199‐208. 31228518 10.1016/j.yjmcc.2019.06.013 PMC6705397

[311] Rossano J , Lin K , Epstein S , et al. Safety Profile Of The First Pediatric Cardiomyopathy Gene Therapy Trial: RP‐A501 (AAV9:LAMP2B) For Danon Disease. Journal of Cardiac Failure. 2023;29(4):554.

[312] Colella P , Mingozzi F . Gene Therapy for Pompe Disease: The Time is now. Hum Gene Ther. 2019;30(10):1245‐1262. 31298581 10.1089/hum.2019.109

[313] Pauly DF , Johns DC , Matelis LA , Lawrence JH , Byrne BJ , Kessler PD . Complete correction of acid alpha‐glucosidase deficiency in Pompe disease fibroblasts in vitro, and lysosomally targeted expression in neonatal rat cardiac and skeletal muscle. Gene Ther. 1998;5(4):473‐480. 9614571 10.1038/sj.gt.3300609

[314] Mah C , Pacak CA , Cresawn KO , et al. Physiological correction of Pompe disease by systemic delivery of adeno‐associated virus serotype 1 vectors. Mol Ther. 2007;15(3):501‐507. 17245350 10.1038/sj.mt.6300100

[315] Keeler AM , Zieger M , Todeasa SH , et al. Systemic Delivery of AAVB1‐GAA Clears Glycogen and Prolongs Survival in a Mouse Model of Pompe Disease. Hum Gene Ther. 2019;30(1):57‐68. 29901418 10.1089/hum.2018.016 PMC6343199

[316] Stok M , de Boer H , Huston MW , et al. Lentiviral Hematopoietic Stem Cell Gene Therapy Corrects Murine Pompe Disease. Mol Ther Methods Clin Dev. 2020;17:1014‐1025. 32462050 10.1016/j.omtm.2020.04.023 PMC7240064

[317] Liang Q , Catalano F , Vlaar EC , et al. IGF2‐tagging of GAA promotes full correction of murine Pompe disease at a clinically relevant dosage of lentiviral gene therapy. Mol Ther Methods Clin Dev. 2022;27:109‐130. 36284764 10.1016/j.omtm.2022.09.010 PMC9573825

[318] Meyers DE , Basha HI , Koenig MK . Mitochondrial cardiomyopathy: pathophysiology, diagnosis, and management. Tex Heart Inst J. 2013;40(4):385‐394. 24082366 PMC3783139

[319] Wang G , McCain ML , Yang L , et al. Modeling the mitochondrial cardiomyopathy of Barth syndrome with induced pluripotent stem cell and heart‐on‐chip technologies. Nat Med. 2014;20(6):616‐623. 24813252 10.1038/nm.3545 PMC4172922

[320] Zegallai HM , Hatch GM . Barth syndrome: cardiolipin, cellular pathophysiology, management, and novel therapeutic targets. Mol Cell Biochem. 2021;476(3):1605‐1629. 33415565 10.1007/s11010-020-04021-0

[321] Suzuki‐Hatano S , Saha M , Rizzo SA , et al. AAV‐Mediated TAZ Gene Replacement Restores Mitochondrial and Cardioskeletal Function in Barth Syndrome. Hum Gene Ther. 2019;30(2):139‐154. 30070157 10.1089/hum.2018.020 PMC6383582

[322] Yue P , Zhang Y , Liu L , et al. Yap1 modulates cardiomyocyte hypertrophy via impaired mitochondrial biogenesis in response to chronic mechanical stress overload. Theranostics. 2022;12(16):7009‐7031. 36276651 10.7150/thno.74563 PMC9576622

[323] Salvarani N , Crasto S , Miragoli M , et al. The K219T‐Lamin mutation induces conduction defects through epigenetic inhibition of SCN5A in human cardiac laminopathy. Nat Commun. 2019;10(1):2267. 31118417 10.1038/s41467-019-09929-w PMC6531493

[324] Gerbino A , Bottillo I , Milano S , et al. Functional Characterization of a Novel Truncating Mutation in Lamin A/C Gene in a Family with a Severe Cardiomyopathy with Conduction Defects. Cell Physiol Biochem. 2017;44(4):1559‐1577. 29197877 10.1159/000485651

[325] Sun Y , Guo C , Chen Z , et al. Non‐cell autonomous cardiomyocyte regulation complicates gene supplementation therapy for LMNA cardiomyopathy. bioRxiv . 2023:2023.07.18.549413. 10.1016/j.jacbts.2024.06.004 PMC11604484 39619139

[326] Lee J , Termglinchan V , Diecke S , et al. Activation of PDGF pathway links LMNA mutation to dilated cardiomyopathy. Nature. 2019;572(7769):335‐340. 31316208 10.1038/s41586-019-1406-x PMC6779479

[327] Chen SN , Lombardi R , Karmouch J , et al. DNA Damage Response/TP53 Pathway Is Activated and Contributes to the Pathogenesis of Dilated Cardiomyopathy Associated With LMNA (Lamin A/C) Mutations. Circ Res. 2019;124(6):856‐873. 30696354 10.1161/CIRCRESAHA.118.314238 PMC6460911

[328] Muchir A , Worman HJ . Targeting Mitogen‐Activated Protein Kinase Signaling in Mouse Models of Cardiomyopathy Caused by Lamin A/C Gene Mutations. Methods Enzymol. 2016;568:557‐580. 26795484 10.1016/bs.mie.2015.07.028 PMC4878678

[329] Choi JC , Muchir A , Wu W , et al. Temsirolimus activates autophagy and ameliorates cardiomyopathy caused by lamin A/C gene mutation. Sci Transl Med. 2012;4(144):144ra102. 10.1126/scitranslmed.3003875 PMC3700376 22837537

[330] Chatzifrangkeskou M , Le Dour C , Wu W , et al. ERK1/2 directly acts on CTGF/CCN2 expression to mediate myocardial fibrosis in cardiomyopathy caused by mutations in the lamin A/C gene. Hum Mol Genet. 2016;25(11):2220‐2233. 27131347 10.1093/hmg/ddw090 PMC5081054

[331] Wu W , Muchir A , Shan J , Bonne G , Worman HJ . Mitogen‐activated protein kinase inhibitors improve heart function and prevent fibrosis in cardiomyopathy caused by mutation in lamin A/C gene. Circulation. 2011;123(1):53‐61. 21173351 10.1161/CIRCULATIONAHA.110.970673 PMC3061281

[332] Tan CY , Wong JX , Chan PS , et al. Yin Yang 1 Suppresses Dilated Cardiomyopathy and Cardiac Fibrosis Through Regulation of Bmp7 and Ctgf. Circ Res. 2019;125(9):834‐846. 31495264 10.1161/CIRCRESAHA.119.314794 PMC7336364

[333] Brugada J , Campuzano O , Arbelo E , Sarquella‐Brugada G , Brugada R . Present Status of Brugada Syndrome: JACC State‐of‐the‐Art Review. J Am Coll Cardiol. 2018;72(9):1046‐1059. 30139433 10.1016/j.jacc.2018.06.037

[334] Zaklyazminskaya E , Dzemeshkevich S . The role of mutations in the SCN5A gene in cardiomyopathies. Biochim Biophys Acta. 2016;1863(7 Pt B):1799‐805. 26916278 10.1016/j.bbamcr.2016.02.014

[335] Brugada R , Campuzano O , Sarquella‐Brugada G , Brugada P , Brugada J , Hong K . Brugada Syndrome. In: Adam MP , Everman DB , Mirzaa GM , et al, eds. *GeneReviews(®)*. University of Washington, Seattle. Copyright © 1993–2023, GeneReviews is a registered trademark of the University of Washington, University of Washington, Seattle, Seattle. All rights reserved.; 1993. 20301690

[336] Wilde AAM , Amin AS . Clinical Spectrum of SCN5A Mutations: Long QT Syndrome, Brugada Syndrome, and Cardiomyopathy. JACC Clin Electrophysiol. 2018;4(5):569‐579. 29798782 10.1016/j.jacep.2018.03.006

[337] Hu D , Barajas‐Martínez H , Pfeiffer R , et al. Mutations in SCN10A are responsible for a large fraction of cases of Brugada syndrome. J Am Coll Cardiol. 2014;64(1):66‐79. 24998131 10.1016/j.jacc.2014.04.032 PMC4116276

[338] Ciconte G , Monasky MM , Santinelli V , et al. Brugada syndrome genetics is associated with phenotype severity. Eur Heart J. 2021;42(11):1082‐1090. 33221895 10.1093/eurheartj/ehaa942 PMC7955973

[339] Liantonio A , Bertini M , Mele A , et al. Brugada Syndrome: More than a Monogenic Channelopathy. Biomedicines. 2023;11(8):2297. 37626795 10.3390/biomedicines11082297 PMC10452102

[340] Curcio A , Santarpia G , Indolfi C . The Brugada Syndrome ‐ From Gene to Therapy. Circ J. 2017;81(3):290‐297. 28070060 10.1253/circj.CJ-16-0971

[341] Liang P , Sallam K , Wu H , et al. Patient‐Specific and Genome‐Edited Induced Pluripotent Stem Cell‐Derived Cardiomyocytes Elucidate Single‐Cell Phenotype of Brugada Syndrome. J Am Coll Cardiol. 2016;68(19):2086‐2096. 27810048 10.1016/j.jacc.2016.07.779 PMC5373649

[342] Teng S , Huang J , Gao Z , et al. Readthrough of SCN5A Nonsense Mutations p.R1623X and p.S1812X Questions Gene‐therapy in Brugada Syndrome. Curr Gene Ther. 2017;17(1):50‐58. 28552050 10.2174/1566523217666170529074758

[343] Yu G , Chakrabarti S , Tischenko M , et al. Gene therapy targeting protein trafficking regulator MOG1 in mouse models of Brugada syndrome, arrhythmias, and mild cardiomyopathy. Sci Transl Med. 2022;14(648):eabf3136. 35675436 10.1126/scitranslmed.abf3136

[344] Chakrabarti S , Wu X , Yang Z , et al. MOG1 rescues defective trafficking of Na(v)1.5 mutations in Brugada syndrome and sick sinus syndrome. Circ Arrhythm Electrophysiol. 2013;6(2):392‐401. 23420830 10.1161/CIRCEP.111.000206 PMC3633223

[345] Schwartz PJ , Ackerman MJ , George AL, Jr. , Wilde AAM . Impact of genetics on the clinical management of channelopathies. J Am Coll Cardiol. 2013;62(3):169‐180. 23684683 10.1016/j.jacc.2013.04.044 PMC3710520

[346] Li W , Yin L , Shen C , Hu K , Ge J , Sun A . SCN5A Variants: Association With Cardiac Disorders. Front Physiol. 2018;9:1372. 30364184 10.3389/fphys.2018.01372 PMC6191725

[347] Zhao Z , Zang X , Niu K , et al. Impacts of gene variants on drug effects‐the foundation of genotype‐guided pharmacologic therapy for long QT syndrome and short QT syndrome. EBioMedicine. 2024;103:105108. 38653189 10.1016/j.ebiom.2024.105108 PMC11041837

[348] Kim M , Sager PT , Tester DJ , et al. SGK1 inhibition attenuates the action potential duration in reengineered heart cell models of drug‐induced QT prolongation. Heart Rhythm. 2023;20(4):589‐595. 36610526 10.1016/j.hrthm.2022.12.036

[349] Bezzerides VJ , Zhang A , Xiao L , et al. Inhibition of serum and glucocorticoid regulated kinase‐1 as novel therapy for cardiac arrhythmia disorders. Sci Rep. 2017;7(1):346. 28336914 10.1038/s41598-017-00413-3 PMC5428512

[350] Philippaert K , Kalyaanamoorthy S , Fatehi M , et al. Cardiac Late Sodium Channel Current Is a Molecular Target for the Sodium/Glucose Cotransporter 2 Inhibitor Empagliflozin. Circulation. 2021;143(22):2188‐2204. 33832341 10.1161/CIRCULATIONAHA.121.053350 PMC8154177

[351] Dotzler SM , Kim CSJ , Gendron WAC , et al. Suppression‐Replacement KCNQ1 Gene Therapy for Type 1 Long QT Syndrome. Circulation. 2021;143(14):1411‐1425. 33504163 10.1161/CIRCULATIONAHA.120.051836

[352] Bjerregaard P . Diagnosis and management of short QT syndrome. Heart Rhythm. 2018;15(8):1261‐1267. 29501667 10.1016/j.hrthm.2018.02.034

[353] Rudic B , Schimpf R , Borggrefe M . Short QT Syndrome ‐ Review of Diagnosis and Treatment. Arrhythm Electrophysiol Rev. 2014;3(2):76‐79. 26835070 10.15420/aer.2014.3.2.76 PMC4711567

[354] Bjerregaard P , Jahangir A , Gussak I . Targeted therapy for short QT syndrome. Expert Opin Ther Targets. 2006;10(3):393‐400. 16706679 10.1517/14728222.10.3.393

[355] Hancox JC , Whittaker DG , Du C , Stuart AG , Zhang H . Emerging therapeutic targets in the short QT syndrome. Expert Opin Ther Targets. 2018;22(5):439‐451. 29697308 10.1080/14728222.2018.1470621

[356] Zheng J , Huang E , Tang S , et al. A case‐control study of sudden unexplained nocturnal death syndrome in the southern Chinese Han population. Am J Forensic Med Pathol. 2015;36(1):39‐43. 25621881 10.1097/PAF.0000000000000135

[357] Lehnart SE , Ackerman MJ , Benson DW, Jr. , et al. Inherited arrhythmias: a National Heart, Lung, and Blood Institute and Office of Rare Diseases workshop consensus report about the diagnosis, phenotyping, molecular mechanisms, and therapeutic approaches for primary cardiomyopathies of gene mutations affecting ion channel function. Circulation. 2007;116(20):2325‐2345. 17998470 10.1161/CIRCULATIONAHA.107.711689

[358] Ackerman MJ , Priori SG , Willems S , et al. HRS/EHRA expert consensus statement on the state of genetic testing for the channelopathies and cardiomyopathies this document was developed as a partnership between the Heart Rhythm Society (HRS) and the European Heart Rhythm Association (EHRA). Heart Rhythm. 2011;8(8):1308‐1339. 21787999 10.1016/j.hrthm.2011.05.020

[359] Senapati A , Sperry BW , Grodin JL , et al. Prognostic implication of relative regional strain ratio in cardiac amyloidosis. Heart. 2016;102(10):748‐754. 26830665 10.1136/heartjnl-2015-308657

[360] Sperry BW , Tang WHW . Amyloid heart disease: genetics translated into disease‐modifying therapy. Heart. 2017;103(11):812‐817. 28255101 10.1136/heartjnl-2016-309914

[361] Sperry BW , Vranian MN , Hachamovitch R , et al. Subtype‐Specific Interactions and Prognosis in Cardiac Amyloidosis. J Am Heart Assoc. 2016;5(3):e002877. 27013539 10.1161/JAHA.115.002877 PMC4943263

[362] Maurer MS , Hanna M , Grogan M , et al. Genotype and Phenotype of Transthyretin Cardiac Amyloidosis: THAOS (Transthyretin Amyloid Outcome Survey). J Am Coll Cardiol. 2016;68(2):161‐172. 27386769 10.1016/j.jacc.2016.03.596 PMC4940135

[363] Mankad AK , Sesay I , Shah KB . Light‐chain cardiac amyloidosis. Curr Probl Cancer. 2017;41(2):144‐156. 28117074 10.1016/j.currproblcancer.2016.11.004

[364] Gandhi UH , Cornell RF , Lakshman A , et al. Outcomes of patients with multiple myeloma refractory to CD38‐targeted monoclonal antibody therapy. Leukemia. 2019;33(9):2266‐2275. 30858549 10.1038/s41375-019-0435-7 PMC6820050

[365] Lachmann HJ , Booth DR , Booth SE , et al. Misdiagnosis of hereditary amyloidosis as AL (primary) amyloidosis. N Engl J Med. 2002;346(23):1786‐1791. 12050338 10.1056/NEJMoa013354

[366] Planté‐Bordeneuve V , Said G . Familial amyloid polyneuropathy. Lancet Neurol. 2011;10(12):1086‐1097. 22094129 10.1016/S1474-4422(11)70246-0

[367] Suhr OB , Lindqvist P , Olofsson BO , Waldenström A , Backman C . Myocardial hypertrophy and function are related to age at onset in familial amyloidotic polyneuropathy. Amyloid. 2006;13(3):154‐159. 17062381 10.1080/13506120600876849

[368] Buxbaum J , Alexander A , Koziol J , Tagoe C , Fox E , Kitzman D . Significance of the amyloidogenic transthyretin Val 122 Ile allele in African Americans in the Arteriosclerosis Risk in Communities (ARIC) and Cardiovascular Health (CHS) Studies. Am Heart J. 2010;159(5):864‐870. 20435197 10.1016/j.ahj.2010.02.006 PMC2865207

[369] Dungu JN , Papadopoulou SA , Wykes K , et al. Afro‐Caribbean Heart Failure in the United Kingdom: Cause, Outcomes, and ATTR V122I Cardiac Amyloidosis. Circ Heart Fail. 2016;9(9):e003352. 27618855 10.1161/CIRCHEARTFAILURE.116.003352

[370] Sattianayagam PT , Hahn AF , Whelan CJ , et al. Cardiac phenotype and clinical outcome of familial amyloid polyneuropathy associated with transthyretin alanine 60 variant. Eur Heart J. 2012;33(9):1120‐1127. 21992998 10.1093/eurheartj/ehr383

[371] Nehashi T , Oikawa M , Amami K , et al. Sporadic Cardiac Amyloidosis by Amyloidogenic Transthyretin V122I Variant. Int Heart J. 2019;60(6):1441‐1443. 31666456 10.1536/ihj.19-134

[372] Nakase T , Yamashita T , Matsuo Y , et al. Hereditary ATTR Amyloidosis with Cardiomyopathy Caused by the Novel Variant Transthyretin Y114S (p.Y134S). Intern Med. 2019;58(18):2695‐2698. 31178489 10.2169/internalmedicine.2456-18 PMC6794161

[373] Bauer R , Dikow N , Brauer A , et al. The “Wagshurst study”: p.Val40Ile transthyretin gene variant causes late‐onset cardiomyopathy. Amyloid. 2014;21(4):267‐275. 25291558 10.3109/13506129.2014.967846

[374] Papathanasiou M , Carpinteiro A , Kersting D , et al. Rare variant (p.Ser43Asn) of familial transthyretin amyloidosis associated with isolated cardiac phenotype: A case series with literature review. Mol Genet Genomic Med. 2021;9(12):e1581. 33345470 10.1002/mgg3.1581 PMC8683619

[375] Kittleson MM , Maurer MS , Ambardekar AV , et al. Cardiac Amyloidosis: Evolving Diagnosis and Management: A Scientific Statement From the American Heart Association. Circulation. 2020;142(1):e7‐e22. 32476490 10.1161/CIR.0000000000000792

[376] Sekijima Y . Transthyretin (ATTR) amyloidosis: clinical spectrum, molecular pathogenesis and disease‐modifying treatments. J Neurol Neurosurg Psychiatry. 2015;86(9):1036‐1043. 25604431 10.1136/jnnp-2014-308724

[377] Butler JS , Chan A , Costelha S , et al. Preclinical evaluation of RNAi as a treatment for transthyretin‐mediated amyloidosis. Amyloid. 2016;23(2):109‐118. 27033334 10.3109/13506129.2016.1160882 PMC4898164

[378] Mathew V , Wang AK . Inotersen: new promise for the treatment of hereditary transthyretin amyloidosis. Drug Des Devel Ther. 2019;13:1515‐1525. 10.2147/DDDT.S162913 PMC6507904 31118583

[379] Benson MD , Kluve‐Beckerman B , Zeldenrust SR , et al. Targeted suppression of an amyloidogenic transthyretin with antisense oligonucleotides. Muscle Nerve. 2006;33(5):609‐618. 16421881 10.1002/mus.20503

[380] Benson MD , Dasgupta NR , Rissing SM , Smith J , Feigenbaum H . Safety and efficacy of a TTR specific antisense oligonucleotide in patients with transthyretin amyloid cardiomyopathy. Amyloid. 2017;24(4):219‐225. 28906150 10.1080/13506129.2017.1374946

[381] Gillmore JD , Gane E , Taubel J , et al. CRISPR‐Cas9 In Vivo Gene Editing for Transthyretin Amyloidosis. N Engl J Med. 2021;385(6):493‐502. 34215024 10.1056/NEJMoa2107454

[382] Mercuri E , Bönnemann CG , Muntoni F . Muscular dystrophies. Lancet. 2019;394(10213):2025‐2038. 31789220 10.1016/S0140-6736(19)32910-1

[383] Wong TWY , Ahmed A , Yang G , et al. A novel mouse model of Duchenne muscular dystrophy carrying a multi‐exonic Dmd deletion exhibits progressive muscular dystrophy and early‐onset cardiomyopathy. Dis Model Mech. 2020;13(9):dmm045369. 32988972 10.1242/dmm.045369 PMC7522028

[384] Yue Y , Binalsheikh IM , Leach SB , Domeier TL , Duan D . Prospect of gene therapy for cardiomyopathy in hereditary muscular dystrophy. Expert Opin Orphan Drugs. 2016;4(2):169‐183. 27340611 10.1517/21678707.2016.1124039 PMC4914135

[385] Duan D . Micro‐Dystrophin Gene Therapy Goes Systemic in Duchenne Muscular Dystrophy Patients. Hum Gene Ther. 2018;29(7):733‐736. 29463117 10.1089/hum.2018.012 PMC6066190

[386] Duan D . Systemic AAV Micro‐dystrophin Gene Therapy for Duchenne Muscular Dystrophy. Mol Ther. 2018;26(10):2337‐2356. 30093306 10.1016/j.ymthe.2018.07.011 PMC6171037

[387] Bostick B , Yue Y , Lai Y , Long C , Li D , Duan D . Adeno‐associated virus serotype‐9 microdystrophin gene therapy ameliorates electrocardiographic abnormalities in mdx mice. Hum Gene Ther. 2008;19(8):851‐856. 18666839 10.1089/hum.2008.058 PMC2888653

[388] Bostick B , Shin JH , Yue Y , Wasala NB , Lai Y , Duan D . AAV micro‐dystrophin gene therapy alleviates stress‐induced cardiac death but not myocardial fibrosis in >21‐m‐old mdx mice, an end‐stage model of Duchenne muscular dystrophy cardiomyopathy. J Mol Cell Cardiol. 2012;53(2):217‐222. 22587991 10.1016/j.yjmcc.2012.05.002 PMC3389274

[389] Bauer R , Enns H , Jungmann A , et al. Various effects of AAV9‐mediated βARKct gene therapy on the heart in dystrophin‐deficient (mdx) mice and δ‐sarcoglycan‐deficient (Sgcd‐/‐) mice. Neuromuscul Disord. 2019;29(3):231‐241. 30782477 10.1016/j.nmd.2018.12.006

[390] Xu R , Jia Y , Zygmunt DA , Martin PT . rAAVrh74.MCK.GALGT2 Protects against Loss of Hemodynamic Function in the Aging mdx Mouse Heart. Mol Ther. 2019;27(3):636‐649. 30711447 10.1016/j.ymthe.2019.01.005 PMC6403484

[391] El Refaey M , Xu L , Gao Y , et al. In Vivo Genome Editing Restores Dystrophin Expression and Cardiac Function in Dystrophic Mice. Circ Res. 2017;121(8):923‐929. 28790199 10.1161/CIRCRESAHA.117.310996 PMC5623072

[392] Amoasii L , Hildyard JCW , Li H , et al. Gene editing restores dystrophin expression in a canine model of Duchenne muscular dystrophy. Science. 2018;362(6410):86‐91. 30166439 10.1126/science.aau1549 PMC6205228

[393] Xu L , Lau YS , Gao Y , Li H , Han R . Life‐Long AAV‐Mediated CRISPR Genome Editing in Dystrophic Heart Improves Cardiomyopathy without Causing Serious Lesions in mdx Mice. Mol Ther. 2019;27(8):1407‐1414. 31129119 10.1016/j.ymthe.2019.05.001 PMC6697345

[394] Hakim CH , Wasala NB , Nelson CE , et al. AAV CRISPR editing rescues cardiac and muscle function for 18 months in dystrophic mice. JCI Insight. 2018;3(23):e124297. 30518686 10.1172/jci.insight.124297 PMC6328021

[395] Long C , Amoasii L , Mireault AA , et al. Postnatal genome editing partially restores dystrophin expression in a mouse model of muscular dystrophy. Science. 2016;351(6271):400‐403. 26721683 10.1126/science.aad5725 PMC4760628

[396] Nelson CE , Hakim CH , Ousterout DG , et al. In vivo genome editing improves muscle function in a mouse model of Duchenne muscular dystrophy. Science. 2016;351(6271):403‐407. 26721684 10.1126/science.aad5143 PMC4883596

[397] Tabebordbar M , Zhu K , Cheng JKW , et al. In vivo gene editing in dystrophic mouse muscle and muscle stem cells. Science. 2016;351(6271):407‐411. 26721686 10.1126/science.aad5177 PMC4924477

[398] Wang JZ , Wu P , Shi ZM , Xu YL , Liu ZJ . The AAV‐mediated and RNA‐guided CRISPR/Cas9 system for gene therapy of DMD and BMD. Brain Dev. 2017;39(7):547‐556. 28390761 10.1016/j.braindev.2017.03.024

[399] Zhang Y , Long C , Li H , et al. CRISPR‐Cpf1 correction of muscular dystrophy mutations in human cardiomyocytes and mice. Sci Adv. 2017;3(4):e1602814. 28439558 10.1126/sciadv.1602814 PMC5389745

[400] Goyenvalle A , Griffith G , Babbs A , et al. Functional correction in mouse models of muscular dystrophy using exon‐skipping tricyclo‐DNA oligomers. Nat Med. 2015;21(3):270‐275. 25642938 10.1038/nm.3765

[401] Rodino‐Klapac LR . MicroRNA based treatment of cardiomyopathy: not all dystrophies are created equal. J Am Heart Assoc. 2013;2(4):e000384. 23969225 10.1161/JAHA.113.000384 PMC3828818

[402] Iwata Y , Matsumura T . Blockade of TRPV2 is a Novel Therapy for Cardiomyopathy in Muscular Dystrophy. Int J Mol Sci. 2019;20(16):3844. 31394715 10.3390/ijms20163844 PMC6720432

[403] Benjamin EJ , Muntner P , Alonso A , et al. Heart Disease and Stroke Statistics‐2019 Update: A Report From the American Heart Association. Circulation. 2019;139(10):e56‐e528. 30700139 10.1161/CIR.0000000000000659

[404] Iqbal J , Zhang YJ , Holmes DR , et al. Optimal medical therapy improves clinical outcomes in patients undergoing revascularization with percutaneous coronary intervention or coronary artery bypass grafting: insights from the Synergy Between Percutaneous Coronary Intervention with TAXUS and Cardiac Surgery (SYNTAX) trial at the 5‐year follow‐up. Circulation. 2015;131(14):1269‐1277. 25847979 10.1161/CIRCULATIONAHA.114.013042

[405] Hinkel R , Trenkwalder T , Kupatt C . Gene therapy for ischemic heart disease. Expert Opin Biol Ther. 2011;11(6):723‐737. 21434842 10.1517/14712598.2011.570749

[406] Pastena P , Frye JT , Ho C , Goldschmidt ME , Kalogeropoulos AP . Ischemic cardiomyopathy: epidemiology, pathophysiology, outcomes, and therapeutic options. Heart Fail Rev. 2024;29(1):287‐299. 38103139 10.1007/s10741-023-10377-4

[407] Ye L , Haider H , Jiang S , et al. Angiopoietin‐1 for myocardial angiogenesis: a comparison between delivery strategies. Eur J Heart Fail. 2007;9(5):458‐465. 17188570 10.1016/j.ejheart.2006.10.022

[408] Abdelwahid E , Kalvelyte A , Stulpinas A , de Carvalho KA , Guarita‐Souza LC , Foldes G . Stem cell death and survival in heart regeneration and repair. Apoptosis. 2016;21(3):252‐268. 26687129 10.1007/s10495-015-1203-4 PMC5200890

[409] Marotta P , Cianflone E , Aquila I , et al. Combining cell and gene therapy to advance cardiac regeneration. Expert Opin Biol Ther. 2018;18(4):409‐423. 29347847 10.1080/14712598.2018.1430762

[410] Laakkonen JP , Lähteenvuo J , Jauhiainen S , Heikura T , Ylä‐Herttuala S . Beyond endothelial cells: Vascular endothelial growth factors in heart, vascular anomalies and placenta. Vascul Pharmacol. 2019;112:91‐101. 30342234 10.1016/j.vph.2018.10.005

[411] Bates DO , Beazley‐Long N , Benest AV , et al. Physiological Role of Vascular Endothelial Growth Factors as Homeostatic Regulators. Compr Physiol. 2018;8(3):955‐979. 29978898 10.1002/cphy.c170015

[412] Koch S , Tugues S , Li X , Gualandi L , Claesson‐Welsh L . Signal transduction by vascular endothelial growth factor receptors. Biochem J. 2011;437(2):169‐183. 21711246 10.1042/BJ20110301

[413] Mason D , Chen YZ , Krishnan HV , Sant S . Cardiac gene therapy: Recent advances and future directions. J Control Release. 2015;215:101‐111. 26254712 10.1016/j.jconrel.2015.08.001

[414] Hedman M , Hartikainen J , Syvänne M , et al. Safety and feasibility of catheter‐based local intracoronary vascular endothelial growth factor gene transfer in the prevention of postangioplasty and in‐stent restenosis and in the treatment of chronic myocardial ischemia: phase II results of the Kuopio Angiogenesis Trial (KAT). Circulation. 2003;107(21):2677‐2683. 12742981 10.1161/01.CIR.0000070540.80780.92

[415] Hedman M , Muona K , Hedman A , et al. Eight‐year safety follow‐up of coronary artery disease patients after local intracoronary VEGF gene transfer. Gene Ther. 2009;16(5):629‐634. 19212427 10.1038/gt.2009.4

[416] Stewart DJ , Hilton JD , Arnold JM , et al. Angiogenic gene therapy in patients with nonrevascularizable ischemic heart disease: a phase 2 randomized, controlled trial of AdVEGF(121) (AdVEGF121) versus maximum medical treatment. Gene Ther. 2006;13(21):1503‐1511. 16791287 10.1038/sj.gt.3302802

[417] Becher B , Tugues S , Greter M . GM‐CSF: From Growth Factor to Central Mediator of Tissue Inflammation. Immunity. 2016;45(5):963‐973. 27851925 10.1016/j.immuni.2016.10.026

[418] Anzai A , Choi JL , He S , et al. The infarcted myocardium solicits GM‐CSF for the detrimental oversupply of inflammatory leukocytes. J Exp Med. 2017;214(11):3293‐3310. 28978634 10.1084/jem.20170689 PMC5679174

[419] Seiler C , Pohl T , Wustmann K , et al. Promotion of collateral growth by granulocyte‐macrophage colony‐stimulating factor in patients with coronary artery disease: a randomized, double‐blind, placebo‐controlled study. Circulation. 2001;104(17):2012‐2017. 11673338 10.1161/hc4201.097835

[420] Parissis JT , Adamopoulos S , Venetsanou K , et al. Plasma profiles of circulating granulocyte‐macrophage colony‐stimulating factor and soluble cellular adhesion molecules in acute myocardial infarction. Contribution to post‐infarction left ventricular dysfunction. Eur Cytokine Netw. 2004;15(2):139‐144. 15319174

[421] Maekawa Y , Anzai T , Yoshikawa T , et al. Effect of granulocyte‐macrophage colony‐stimulating factor inducer on left ventricular remodeling after acute myocardial infarction. J Am Coll Cardiol. 2004;44(7):1510‐1520. 15464336 10.1016/j.jacc.2004.05.083

[422] Hadas Y , Katz MG , Bridges CR , Zangi L . Modified mRNA as a therapeutic tool to induce cardiac regeneration in ischemic heart disease. Wiley Interdiscip Rev Syst Biol Med. 2017;9(1):e1367. 10.1002/wsbm.1367 PMC5880206 27911047

[423] Kurisu S , Kihara Y . Tako‐tsubo cardiomyopathy: clinical presentation and underlying mechanism. J Cardiol. 2012;60(6):429‐437. 23078863 10.1016/j.jjcc.2012.06.015

[424] Sestini S , Coppola A , Dona M , et al. Rethinking Tako‐tsubo Cardiomyopathy: The Contribution of Myocardial Pathology and Molecular Imaging. Curr Radiopharm. 2023;16(4):253‐268. 37190802 10.2174/1874471016666230515142106

[425] Ware JS , Li J , Mazaika E , et al. Shared Genetic Predisposition in Peripartum and Dilated Cardiomyopathies. N Engl J Med. 2016;374(3):233‐241. 26735901 10.1056/NEJMoa1505517 PMC4797319

[426] Sliwa K , Bauersachs J , Arany Z , Spracklen TF , Hilfiker‐Kleiner D . Peripartum cardiomyopathy: from genetics to management. Eur Heart J. 2021;42(32):3094‐3102. 34322694 10.1093/eurheartj/ehab458

[427] Regitz‐Zagrosek V , Roos‐Hesselink JW , Bauersachs J , et al. 2018 ESC Guidelines for the management of cardiovascular diseases during pregnancy. Eur Heart J. 2018;39(34):3165‐3241. 30165544 10.1093/eurheartj/ehy340

[428] Kim DY , Kim SH , Ryu KH . Tachycardia induced Cardiomyopathy. Korean Circ J. 2019;49(9):808‐817. 31456374 10.4070/kcj.2019.0199 PMC6713829

[429] Pollack A , Kontorovich AR , Fuster V , Dec GW . Viral myocarditis–diagnosis, treatment options, and current controversies. Nat Rev Cardiol. 2015;12(11):670‐680. 26194549 10.1038/nrcardio.2015.108

[430] Robinson J , Hartling L , Vandermeer B , Sebastianski M , Klassen TP . Intravenous immunoglobulin for presumed viral myocarditis in children and adults. Cochrane Database Syst Rev. 2020;8(8):Cd004370. 32835416 10.1002/14651858.CD004370.pub4 PMC8210245

[431] Krueger GR , Ablashi DV . Human herpesvirus‐6: a short review of its biological behavior. Intervirology. 2003;46(5):257‐269. 14555846 10.1159/000073205

[432] Sharma A , Marceau C , Hamaguchi R , et al. Human induced pluripotent stem cell‐derived cardiomyocytes as an in vitro model for coxsackievirus B3‐induced myocarditis and antiviral drug screening platform. Circ Res. 2014;115(6):556‐566. 25015077 10.1161/CIRCRESAHA.115.303810 PMC4149868

[433] Katz MG , Fargnoli AS , Kendle AP , Hajjar RJ , Bridges CR . Gene Therapy in Cardiac Surgery: Clinical Trials, Challenges, and Perspectives. Ann Thorac Surg. 2016;101(6):2407‐2416. 26801060 10.1016/j.athoracsur.2015.12.004 PMC4987708

[434] Lu J , Zhang F , Kay MA . A mini‐intronic plasmid (MIP): a novel robust transgene expression vector in vivo and in vitro. Mol Ther. 2013;21(5):954‐963. 23459514 10.1038/mt.2013.33 PMC3666631

[435] Tan A , Rajadas J , Seifalian AM . Exosomes as nano‐theranostic delivery platforms for gene therapy. Adv Drug Deliv Rev. 2013;65(3):357‐367. 22820532 10.1016/j.addr.2012.06.014

[436] Darband SG , Mirza‐Aghazadeh‐Attari M , Kaviani M , et al. Exosomes: natural nanoparticles as bio shuttles for RNAi delivery. J Control Release. 2018;289:158‐170. 30290245 10.1016/j.jconrel.2018.10.001

[437] Zamani P , Fereydouni N , Butler AE , Navashenaq JG , Sahebkar A . The therapeutic and diagnostic role of exosomes in cardiovascular diseases. Trends Cardiovasc Med. 2019;29(6):313‐323. 30385010 10.1016/j.tcm.2018.10.010

[438] Trindade F , Leite‐Moreira A , Ferreira‐Martins J , Ferreira R , Falcão‐Pires I , Vitorino R . Towards the standardization of stem cell therapy studies for ischemic heart diseases: Bridging the gap between animal models and the clinical setting. Int J Cardiol. 2017;228:465‐480. 27870978 10.1016/j.ijcard.2016.11.236

[439] Naftali‐Shani N , Molotski N , Nevo‐Caspi Y , et al. Modeling Peripartum Cardiomyopathy With Human Induced Pluripotent Stem Cells Reveals Distinctive Abnormal Function of Cardiomyocytes. Circulation. 2018;138(23):2721‐2723. 30571272 10.1161/CIRCULATIONAHA.118.035950

[440] Han L , Li Y , Tchao J , et al. Study familial hypertrophic cardiomyopathy using patient‐specific induced pluripotent stem cells. Cardiovasc Res. 2014;104(2):258‐269. 25209314 10.1093/cvr/cvu205 PMC4217687

[441] Arbustini E , Favalli V , Narula N , Serio A , Grasso M . Left Ventricular Noncompaction: A Distinct Genetic Cardiomyopathy? J Am Coll Cardiol. 2016;68(9):949‐966. 27561770 10.1016/j.jacc.2016.05.096

[442] Paszkowska A , Piekutowska‐Abramczuk D , Ciara E , et al. Clinical Presentation of Left Ventricular Noncompaction Cardiomyopathy and Bradycardia in Three Families Carrying HCN4 Pathogenic Variants. Genes (Basel). 2022;13(3):477. 35328031 10.3390/genes13030477 PMC8949387

[443] Nozaki Y , Kato Y , Uike K , et al. Co‐Phenotype of Left Ventricular Non‐Compaction Cardiomyopathy and Atypical Catecholaminergic Polymorphic Ventricular Tachycardia in Association With R169Q, a Ryanodine Receptor Type 2 Missense Mutation. Circ J. 2020;84(2):226‐234. 31875585 10.1253/circj.CJ-19-0720

[444] Yuan C‐C , Kazmierczak K , Liang J , et al. Hypercontractile mutant of ventricular myosin essential light chain leads to disruption of sarcomeric structure and function and results in restrictive cardiomyopathy in mice. Cardiovasc Res. 2017;113(10):1124‐1136. 28371863 10.1093/cvr/cvx060 PMC5852631

